# Characterization of Promiscuous Binding of Phosphor Ligands to Breast-Cancer-Gene 1 (BRCA1) C-Terminal (BRCT): Molecular Dynamics, Free Energy, Entropy and Inhibitor Design

**DOI:** 10.1371/journal.pcbi.1005057

**Published:** 2016-08-25

**Authors:** Wanli You, Yu-ming M. Huang, Smitha Kizhake, Amarnath Natarajan, Chia-en A. Chang

**Affiliations:** 1 Department of Chemistry, University of California, Riverside, Riverside, California, United States of America; 2 Eppley Institute for Research in Cancer and Allied Diseases, University of Nebraska Medical Center, Omaha, Nebraska, United States of America; US Army Medical Research and Materiel Command, UNITED STATES

## Abstract

Inhibition of the protein-protein interaction (PPI) mediated by breast-cancer-gene 1 C-terminal (BRCT) is an attractive strategy to sensitize breast and ovarian cancers to chemotherapeutic agents that induce DNA damage. Such inhibitors could also be used for studies to understand the role of this PPI in DNA damage response. However, design of BRCT inhibitors is challenging because of the inherent flexibility associated with this domain. Several studies identified short phosphopeptides as tight BRCT binders. Here we investigated the thermodynamic properties of 18 phosphopeptides or peptide with phosphate mimic and three compounds with phosphate groups binding to BRCT to understand promiscuous molecular recognition and guide inhibitor design. We performed molecular dynamics (MD) simulations to investigate the interactions between inhibitors and BRCT and their dynamic behavior in the free and bound states. MD simulations revealed the key role of loops in altering the shape and size of the binding site to fit various ligands. The mining minima (M2) method was used for calculating binding free energy to explore the driving forces and the fine balance between configuration entropy loss and enthalpy gain. We designed a rigidified ligand, which showed unfavorable experimental binding affinity due to weakened enthalpy. This was because it lacked the ability to rearrange itself upon binding. Investigation of another phosphate group containing compound, C1, suggested that the entropy loss can be reduced by preventing significant narrowing of the energy well and introducing multiple new compound conformations in the bound states. From our computations, we designed an analog of C1 that introduced new intermolecular interactions to strengthen attractions while maintaining small entropic penalty. This study shows that flexible compounds do not always encounter larger entropy penalty, compared with other more rigid binders, and highlights a new strategy for inhibitor design.

## Author Summary

Promiscuous proteins are commonly observed in biological systems, such as modular domains that recognize phosphopeptides during signal transduction. The use of phosphopeptides and compounds with phosphate groups as inhibitors to protein–protein interactions have attracted increasing interest for years. By using atomistic molecular dynamics simulations, we are able to perform detailed analyses of the dihedral space to explore protein fluctuation upon ligand binding to better understand promiscuous molecular recognition. Free energy calculation can further provide insights into the mechanism of binding, including both enthalpic and entropic contributions for molecular recognition, which assist in inhibitor design. Our calculation results show that pre-rigidifying a ligand is not always advantageous, suggesting the challenge in retaining optimized intermolecular interactions in pre-rigidified ligand. Instead, certain flexible ligands with multiple binding conformations can reduce entropic penalty, and therefore improves binding affinity. According to our computations, we can introduce new intermolecular interactions to flexible ligand to strengthen attractions while maintaining small entropic penalty by retaining its plasticity in the bound conformation. The study might cast light on a new general strategy for designing inhibitors targeting promiscuous modular domains and protein–protein interactions.

## Introduction

The tandem ~100-amino acid repeats of breast-cancer-gene 1 (BRCA1) C-terminal (BRCT) are known to bind to phosphorylated proteins which are important for a number of tumor suppressor functions, which include, DNA repair, cell-cycle checkpoint, and transcription regulation [1–4]. The BRCT repeats recognize and bind phosphorylated protein partners such as CCDC98/Abraxas, BACH1 and CtIP in response to DNA damage [5–10]. Mutations in the BRCT domain of BRCA1 predispose women to breast and ovarian cancers [11]. A recent study showed that inhibitors of BRCT(BRCA1)–phosphoprotein interface can be combined with DNA damaging agents as a viable therapeutic strategy for non-BRCA mutation carriers [12]. The same binding interface on BRCT(BRCA1) promiscuously interacts with various phosphoproteins and short phosphopeptides containing the pSer-X-X-Phe sequence, where X denotes any residue [5–10]. Several modular domains, such as SH3, SH2, FHA, WW, Polo-box and PDZ, are also known to interact with multiple proteins through a consensus recognition sequence [13–18]. Here, we investigated the promiscuous recognition of the BRCT(BRCA1) domain to better understand the mechanism that drives diverse ligands to bind to the same binding site. Our studies will provide insights into molecular detection, inhibitor discovery, and the search for binding partners.

**Fig 1:**
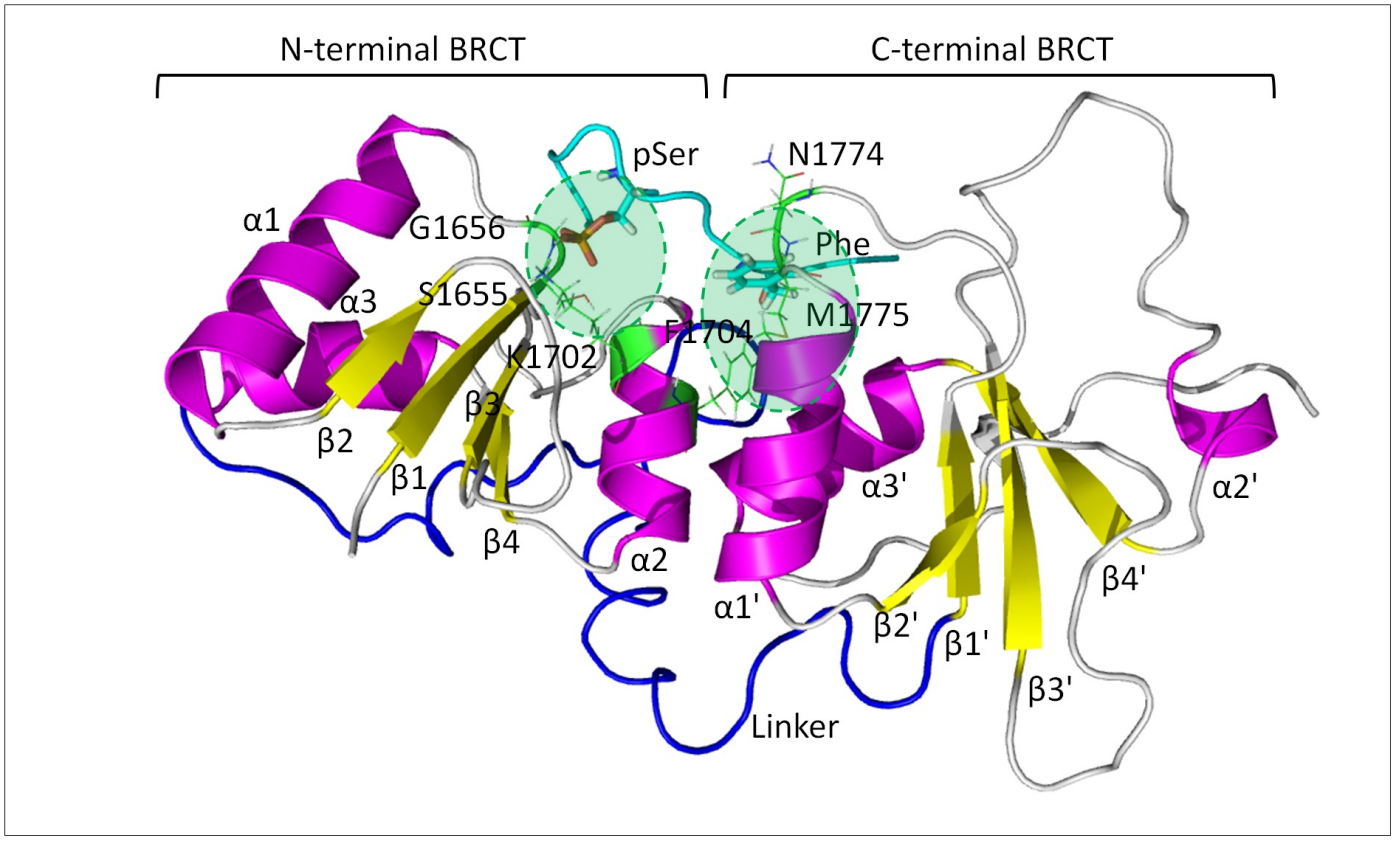
Breast-cancer-gene 1 (BRCA1) C-terminal (BRCT) binding with a phosphoserine (pSer) peptide. pSer forms hydrogen bonds with S1655, G1656 and K1702, and the P+3 Phe locates in the hydrophobic packet formed by M1775, N1774 and F1704. The two points of contact (pSer and P+3 Phe) shown in all of our calculations are highlighted by green circles.

The BRCT(BRCA1) domain is a tandem repeat; each N-terminal BRCT and C-terminal BRCT contain 90–100 residues with a central four-stranded β sheet (β1-β4 and β1ʹ-β4ʹ) and three α-helices (α1-α3 and α1ʹ-α3ʹ). The BRCT–pSXXF interaction is anchored via a two-point binding mode: a hydrophilic contact made by the phosphoserine (pS) residue formed by N-terminal BRCT and a hydrophobic binding pocket from C-terminal BRCT for the phenylalanine (F) residue (Fig 1). The two-point binding scheme is also conserved for compounds with phosphate groups via a phosphate group and a hydrophobic ring group. Unlike most classical pharmaceutical targets such as enzymes with very defined binding cavity, the mostly solvent-exposed and plastic binding pockets such as the phosphoprotein binding interface of BRCT(BRCA1) were considered un-druggable years ago [19–21].

The phosphopeptides are successful inhibitors of protein–protein interactions (PPI) [22–24]. Recently, many new PPI inhibitors have been developed for the BRCT domain, which include a number of short pSXXF tetra-phosphopeptides [12, 24–26] and new phosphopeptide analogs with phosphate groups [27, 28]. Although challenging to design, the demand for inhibitors of PPI has steadily increased [29, 30]. Significant progress has been made in developing inhibitors targeting PPIs, and the development of effective therapeutics from PPI inhibitors will be improved by both experimental and computational approaches.

Recent advances in computer modeling have provided powerful tools to study peptide-domains binding and protein dynamics. Molecular dynamics (MD), Brownian dynamics simulations, and molecular docking have been used to investigate BRCT dynamics, interactions between inhibitors and BRCT, and the ligand association processes [25, 27, 31, 32]. Bioinformatics tools were used to assess the functional impact and likelihood of pathogenicity of variants in the BRCT domain [33, 34]. The promiscuous recognition of BRCT also makes it convenient to investigate the relationship between binding entropy and enthalpy changes. In addition to BRCT, other modular domains serve as good model systems for inspecting promiscuous recognition and the paradox associated with changes in entropy and enthalpy upon ligand binding that targets PPIs by computational methods [35–43].

This study aimed to further understand ligand–BRCT binding and provide strategies for designing inhibitors of PPIs. We selected several tetrapeptides and compounds with phosphate groups to computationally evaluate their driving forces to bind to BRCT(BRCA1). We performed MD simulations and detailed analysis of MD trajectories to examine the approaches BRCT uses to achieve promiscuous binding and the interaction energy of the ligand-BRCT. The MD simulations illustrated the molecular flexibility in the free and bound states for BRCT(BRCA1) and ligands. We analyzed loop movements and the population of dihedral rotations of backbone and side-chains. Conformations from MD simulations were used as initial structures for thorough conformational search and free energy calculations with the M2 method, to reveal the contribution of configuration entropy and enthalpy to ligand binding affinities. We focused on how to optimize the balance between enthalpy gain and entropy loss. Using an accepted practice in ligand design, we synthesized a ligand that incorporates a benzene ring to possibly constrain its conformation. Upon ligand binding, changes of each energy term, conformations, rotameric state, and configurational entropy were evaluated by both MD and M2 tools; and the findings were used to suggest new inhibitors.

## Materials and Methods

### Molecular systems

Table 1 lists 14 short peptides (P1–P14, among which P11 contains a phosphate mimic and others contain phosphorylated amino acids) [24], one compound (C1) [27], one new compound (N1), one designed compound (D1) and 4 long phosphopeptides (L1–L4) that bind to the BRCT domain: pS and pT is phosphorylated amino acid serine and threonine, respectively, and γcE is γ-carboxyglutamate, which is chosen to mimic pS interaction as a non-phosphorylated peptide binder.

**Table 1:** Ligand library of BRCT used for binding affinity exploration and study of flexibility of binding site.

**Tetrapeptides**			
**No.**	**Sequence**	**IC**_**50**_ **(μM)** ^a^	**ΔΔG**_**exp**_ **(kcal/mol)**
P1	Ac-**pS**PT**F**-COOH	1.0±0.2	0
P2	Ac-**pS**PV**F**-COOH	1.6±0.3	0.28
P3	Ac-**pS**PV**F**-CONH_2_	3.2±0.8	0.69
P4	Ac-**pS**PT**F**-CONH_2_	4.6±0.9	0.91
P5	Ac-**pS**PI**F**-CONH_2_	7.1±1.4	1.17
P6	Ac-**pS**PT**Y**-CONH_2_	14.9±2.8	1.61
P7	Ac-**pS**AT**F**-CONH_2_	15.0±1.7	1.61
P8	Ac-**pS**PL**F**-CONH_2_	18.4±1.8	1.74
P9	Ac-**pS**PS**F**-CONH_2_	30.1±7.2	2.03
P10	Ac-**pS**PA**F**-CONH_2_	35.0±7.9	2.12
P11	Ac-**γcE**PT**F**-CONH_2_	52.8±1.6	2.36
P12	Ac-**pS**AA**F**-CONH_2_	98.4±23.1	2.74
P13	Ac-**pS**PP**F**-CONH_2_	>250	>3.29
P14	Ac-**pT**PT**F**-CONH_2_	>250	>3.29
**Compounds**			
**No.**	**Structure**	**IC**_**50**_ **(μM)**	**ΔΔG**_**exp**_ **(kcal/mol)**
C1	See Fig 2	0.31	-0.70
N1	See Fig 2	>250	>3.29
D1	See Fig 2	N/A	N/A
**Long peptides**			
**No.**	**Sequence**	**K**_**d**_ **(μM)** ^b^	**ΔG**_**exp**_ **(kcal/mol)**
L1	ISRST**pS**PT**F**NKQ	0.9	-8.30
L2	PTRVS**pS**PV**F**GA	3.7	-7.46
L3	AAYDI**pS**QV**F**PFA	0.4	-8.78
L4	PQ**pS**PT**F**PEAG	5.2	-7.25

The major binding residues, pSer and Phe (P+3), are in bold. The relative binding free energy for ligand *X* (*X* = P2–P14, C1, N1) to ligand P1 is approximated using the half maximal inhibitory concentration IC50 as ΔΔG_exp_ = RT ln IC50(*X*)/IC50(P1) based on equation Δ*G* = *RT* ln *K*_*d*_ *= RT* ln(IC50 + 0.5*C*_*enzyme*_) ≈ *RT* ln IC50 [44, 45]. Binding free energies for L1–L4 are calculated through equation ΔG_exp_ = RT ln (*K*_*d*_).

^a^ IC50 values of P1–P14 were taken from ref [24]. IC50 values of C1 was taken from ref [27].

^b^ K_d_ values of L1–L4 were taken from ref [46], ref [47], ref [48] and ref [49], respectively.

**Fig 2:**
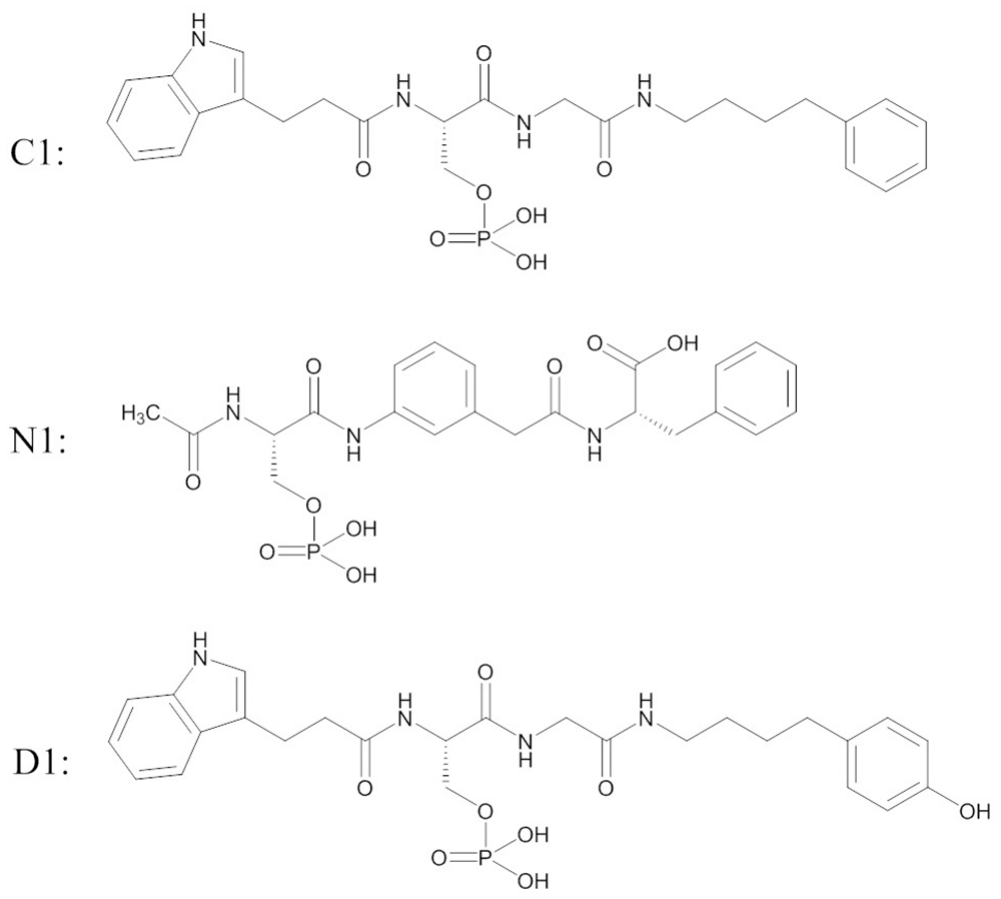
Structures of C1, N1 and D1 that bind to the BRCT domain.

### MD simulations

We ran MD simulations on BRCT-ligand complexes, free ligands and free protein, and the PDB IDs used as initial structures to perform MD simulations were listed in S1 Table. The initial bound conformation of all tetrapeptides was generated by superimposing the backbone atoms of -pSXXF- within phosphorylated BACH1 peptide ISRSTpSPTFNKQ in the C-terminal domain of the BRCA1 protein (PDB code 1T29) [46]. Besides 1T29, we included the other three BRCT domain structures in complex with long phosphopeptides from CtIP, ACC1 proteins and library screening, with PDB IDs 1Y98 (PTRVSpSPVFGA), 3COJ (PQpSPTFPEAG) and 1T2V (AAYDIpSQVFPFA), respectively, for promiscuous molecular recognition study [47–49]. The initial structure of the bound conformation of C1, N1 and D1, where no available crystal structures, were from docking with Autodock tools 1.5.6 [50, 51] and then further checked manually by ensuring important interactions hold. Notably, Autodock was used for only the three ligands that did not have co-crystal structures with BRCT. The docking method used the Lamarckian genetic algorithm, which fixed the protein and allowed the ligand to move around in the docking box. The partial charges of ligands were calculated by using the Vcharge program [52]. The Autodock scoring function is a subset of the AMBER force field that treats molecules using the united atom model. Autogrid version 4.0 was used to create affinity grids with 0.375 Å spacing in 19.5 x 11.25 x 11.25 Å^3^ space at binding site. The final docking result was obtained by 10 runs of simulation with 2.5 million rounds of energy evaluation in each run. Ligand conformations with the lowest docked energies and reasonable conformation (pSer forms hydrogen bonds with S1655, G1656 and K1702, and the P+3 Phe locates in the hydrophobic packet formed by M1775, N1774 and F1704) were further analyzed. We selected two initial conformations with similar low energy computed by Autodock for ligands C1 and D1, and N1 has one initial conformation (S1 Fig).

We performed MD simulations on an apo BRCT domain, 21 complexes, and 21 free ligands to study the dynamic nature of a given system. The standard simulation package, Amber14 [53] with the Amber 99SB force field [54–57], was used. For pSer and pThr, we used the force field reported by Homeyer *et al* [58]. Amber atom types were manually assigned to non-standard amino acid and functional groups of the ligands C1, N1 and D1. Each system was set up as follows. First, we minimized the hydrogen, side-chain and whole system for 500, 5 000 and 5 000 steps, respectively; then the systems were solvated in a rectangular box of a 12-Å explicit TIP3P water model by the tleap program in Amber14. Each system contains about 50 000 atoms. Counter ions Na^+^ were added to keep the whole system neutral, and particle mesh Ewald was used to consider long-range electrostatic interactions [59]. Before equilibration, we ran energy minimization of 10 000 and 20 000 steps for the waters and system, respectively; next, we ran equilibrium of solvent molecules for 40 ps. Then the systems were gradually heated from 250 K for 20 ps, 275 K for 20 ps, to 300 K for 160 ps. We saved a frame every 1 ps with a time step of 2 fs in the isothermic−isobaric (NPT) ensemble. The Langevin thermostat with a damping constant of 2 ps^−1^ was used to maintain a temperature of 300 K, and the hybrid Noseé−Hoover Langevin piston method was used to control the pressure at 1 atm. We also used the SHAKE procedure to constrain hydrogen atoms during MD simulations [60]. Finally, all production runs were performed for 100 ns at 300 K. To ensure that all simulations reached stable energy fluctuations, we considered only trajectories during 20−100 ns for post-analysis.

### M2 method

The second-generation mining minima method, M2, calculates the standard free energy of binding by computing the free energy of the free BRCT (*G°*_*BRCT*_), ligand (*G°*_*ligand*_), and ligand-BRCT complex (*G°*_*comp*_).

$$\Delta G^{o}=G_{Comp}^{o}-G_{BRCT}^{o}-G_{Ligand}^{o}$$ (1)

M2 uses the classical formulation of the partition function for calculating free energy *G°*.

$$G^{o}\approx -RT\text{ln}\left(\frac{8\pi^{2}}{c^{o}}\sum_{i}Z_{i}\right)$$ (2)

$$Z_{i}=\int_{well\;i}e^{-\beta \left(U\left(r\right)+W\left(r\right)\right)}dr\_int$$ (3)

where U is potential energy, W is the solvation free energy and Z_i_ is the local configuration integral from distinct energy wells. The external degrees of freedom were integrated out and C° provides a correction to the standard state, and *r_int* indicates the variables of the internal bond-angle-torsion coordinates. Formally, the configuration integral must be determined over all spaces along the remaining internal degrees of freedom. M2 approximates this configuration integral by using the concept of considering local energy minima only [61, 62]. Therefore, the M2 approach replaces the configurational integral over all spaces with a sum over separate local configurational integrals (Z_i_) associated with the low energy minima of the system. Determining Z_i_ allows for the probability to be associated with each energy well, which in turn, allows for determining a Boltzmann averaged energy <U+W>, which is then subtracted from the total free energy to give the system configurational entropy, useful when analyzing and interpreting predicted binding affinities.

$$-TS_{config}^{o}=G^{o}-<U+W>$$ (4)

Note that the configurational entropy *S°*_*config*_ includes both a conformational part, which reflects the number of energy wells (conformations), and a vibrational part, which reflects the average width of the energy wells. The solvent entropy is included in the solvation free energy, W. Therefore, the computed configurational entropy changes cannot be directly compared with experimentally measured entropy changes, which contain both configurational and solvent entropy.

**Fig 3:**
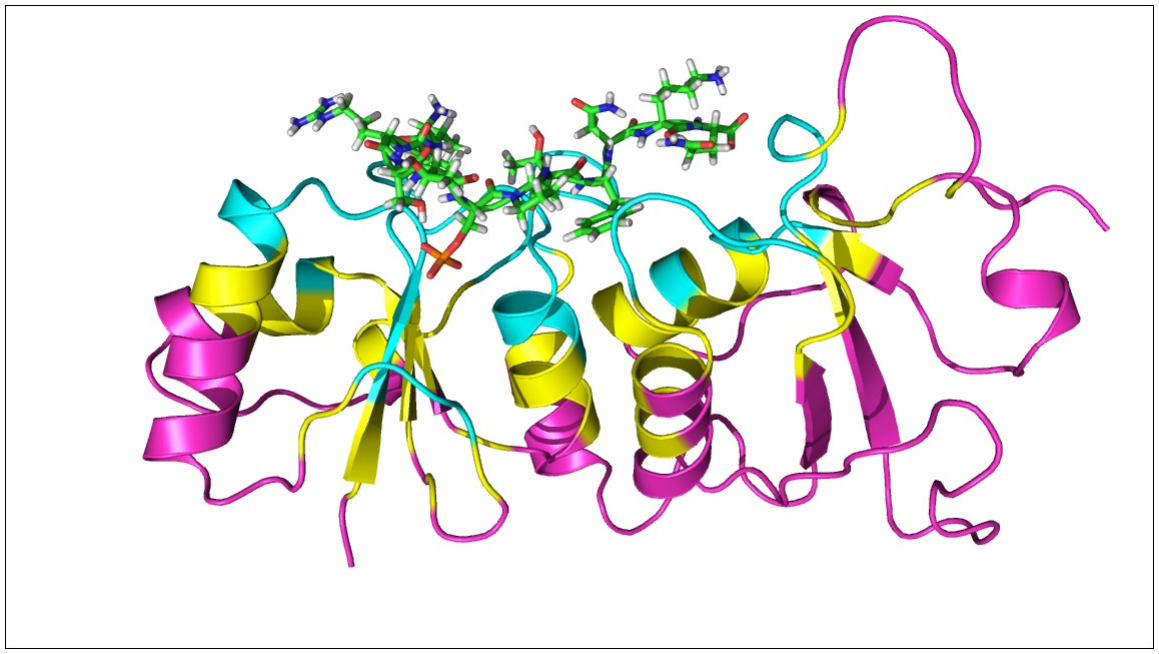
BRCT domain with ligand L1. Ligand L1 is shown in green licorice structure. Only residues within 7 Å of the ligand (live set, labeled in blue) is set flexible in M2 calculations. The rigid set (real set, labeled in yellow) contains the residues within 5 Å of the flexible set; other atoms outside the real set (labeled in pink) were not considered during M2 calculations. Notably, the computed entropy and enthalpy terms from M2 consider the contribution of BRCT (live set) and the ligand.

In brief, M2 contains two parts: 1) an aggressive conformational search for distinct low-energy wells, with repeats detected and removed; and 2) an enhanced harmonic approximation for computing the configuration integral Z_i_ of each well i. Each distinct conformation is energy minimized, first by conjugate gradient method and then by Newton-Raphson method. Both parts involve the Hessian matrix with respect to bond-angle-torsion coordinates, and our harmonic approximation accounts for anharmonicity of eigenvectors of the Hessian matrix with eigenvalues < 2 kcal/mol/Å or 2 kcal/mol/rad. The correlation between different degrees of freedom (e.g., multiple dihedrals may rotate in concert or move with ligand translation/rotation) is captured in the Hessian matrix. We used the VM2 package for the calculation [63–65] and performed three iterations for each ligand and 3 to 10 iterations for the free BRCT and the complexes until the cumulated free energy converged (S2 Fig). To reduce the computational cost, only parts of BRCT were flexible, called the "live set" (Fig 3), which are residues within 7 Å of a long peptide ISRSTpSPTFNKQ in complex with BRCT (PDB code 1T29). The rigid set, called the "real set", contained the residues within 5 Å of the live set. Other atoms not included in these two sets were not considered during the M2 calculations. All ligands were completely flexible and can freely translate and rotate within the binding site, and the same rigid and flexible parts of BRCT were applied to all systems.

### Post-MD analysis: Identifying rotamer states and MM/PBSA calculations

To compare the conformational changes of a molecular system between its free and bound states, we analyzed the selected ligand and BRCT dihedral angles during MD simulations and M2 calculations. Dihedral angles were measured by using T-analyst [66], which can detect the angle population to find discontinuity in a dihedral distribution such as one energy well splitting into two wells near -180° and +180°. A shifted angle by adding or subtracting 360° is then applied to illustrate proper rotamer states. The population of each dihedral was then plotted by using Matlab with a histogram of 144 bins ranging from -360° to +360° to ensure coverage of all rotamer states after angle shifting. When analyzing the rotameric states, because the analysis does not need more than 1000 data points [66], we used trajectories with a smaller file size that a frame was saved every 100 ps (1000 frames) for each 100 ns MD run.

We used the molecular mechanics/Poisson-Boltzmann surface area (MM/PBSA)-type post-processing method to compute ligand-BRCT intermolecular interactions during MD simulations [67–76]. The interaction energy, Δ(U+W) associated with BRCT and a ligand is computed by Δ(U+W) = <E_complex_>—<E_bound BRCT_>—<E_bound ligand_>. The bracket <E> denotes the average energy computed from a given MD trajectory and the energy terms include a valence term (bond, angle and dihedral), van der Waals (U_VDW_), Coulombic (U_Coul_), solvation free energy computed by the Possion-Boltzmann equation (W_PB_) and by cavity/surface area (W_NP_). The dielectric constants of the interior and exterior protein were set to 1 and 80, respectively. The valence term was canceled because of the single trajectory approach.

### Ligand N1 synthesis, purification, and determination of IC50

#### Peptide synthesis

The peptide with the modified amino acid was synthesized by using standard Fmoc chemistry following previously reported methods [24, 77, 78]. The peptide was purified by preparative LC to >95% as assessed by HPLC and characterized by mass spectrometry (S3 Fig).

#### Protein expression and purification

The plasmid construct (pAM15, gift from Luc Gadraeu, University De Sherbrook) encoding six his-tagged BRCT domains of BRCA-1 (amino acids 1646–1859) was used to transform BL21(DE3) RIL (Stratagene). Protein expression was induced by 1mM IPTG and the recombinant protein was purified by nickel affinity chromatography (Qiagen). Homogeneity of the purified protein preparation was assessed by SDS-PAGE and concentration estimated by BCA method (Pierce).

#### Fluorescence polarization assay

The peptide was evaluated in a BRCT assay following previously reported methods [79–81], representative dose-response curves from our previous Fluorescence polarization assay study was shown in S4 Fig. It was carried out in a 384-well low volume corning plate. The polarization and fluorescence were measured on a Spectramax M5 (molecular devices) plate reader. The peptide was titrated into a mixture of BRCT(BRCA1) (1000 nM) and Fluorescently labeled peptide Flu-βA-pSPTF-CONH_2_ (100 nM) where βA is beta-alanine. The IC50 value was calculated by using SigmaPlot. Unfortunately, N1 peptide was inactive even at 1000 μM (1 mM) concentration and all we got was a flat line.

## Results and Discussion

We first applied MD simulations and post-MD analysis for the peptides (P1–P14, L1–L4) and compound C1 to study the fluctuations in various complexes, followed by more rigorous free energy calculations with the M2 method for short peptides (P1–P14) and compound C1 to illustrate detailed energetic and entropic changes upon ligand binding. The new ligand N1 based on consensus ideas that impose structure constraints, was examined experimentally and computationally. Based on our results, compound D1 was derived from the tight binder C1.

### Conformational flexibility of the molecular systems

One unique feature of promiscuous protein systems such as BRCT is to bind to various ligands with significantly different size and shape by using the same binding interface. BRCT needs to provide adequate conformational isomers to recognize these ligands, which involves both side-chain rotation and additional plasticity provided by the backbone. As what shown in crystal structures of BRCT, the relatively rigid alpha helix and beta sheets hold the overall geometry. The variety of side-chains of residues in loops (β3-α2 connection loop, β1'-α1' connection loop and linker between N-terminal and C-terminal) creates a binding surface for ligand recognition except for the reserved binding region for the phosphate group [46]. The backbone nitrogen of G1656 and side-chain of S1655 of the β1 sheet and K1702 of the α2 helix form at least three stable hydrogen bonds with the phosphate group and also orient a ligand in the binding site (Fig 1). Notably, the pocket reserved to bind the phosphate group is located between a structurally rigid region constructed by a helix and a sheet. In contrast, the hydrophobic pocket for the P+3 phenylalanine is built by M1775 and N1774 of the β1'-α1' connection loop and F1704 of the α2 helix, with the β1'-α1' connection loop providing a certain flexibility for peptide binding [48].

**Fig 4:**
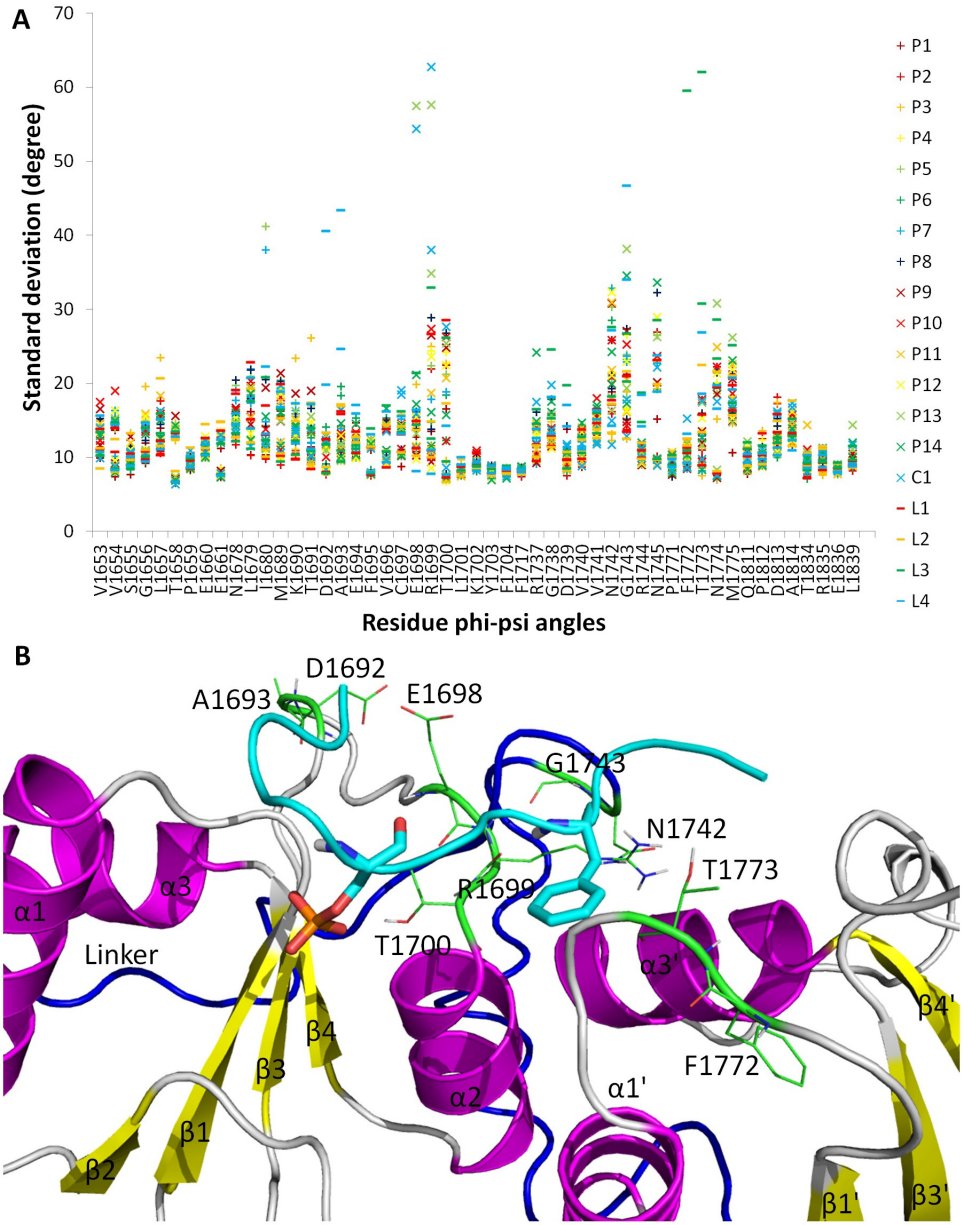
Flexibility of active site of BRCT. (A). Standard deviation of phi and psi angles of the residues of the receptor within 7 Å of ligands during MD simulations. Each residue has one column containing two standard deviation values for the phi angle and psi angle, respectively. (B). Flexible region of the active site. Flexible residues of the protein are shown in a green line representation. Ligand is shown as a blue tube with pSer and Phe (P+3) residues in licorice representation.

To study the flexible regions in the binding pocket of BRCT, we measured the root mean square fluctuation (RMSF) of C_α_ and the standard deviation of phi and psi angles of residues in the BRCT backbone within 7 Å of 18 peptides (P1–P14, L1–L4) and compound C1. The RMSF in S5 Fig shows that residues contacting with a ligand generally have smaller fluctuations and residues without contact with a ligand generally have larger fluctuations, Except for P13, where the middle two proline residues of tetrapeptides do not form optimized contacts with BRCT. Although RMSF plot suggested that residues contacting with a ligand have small fluctuations in the Cartesian space, the standard deviation of phi and psi angles in Fig 4(A) shows that the backbone dihedral angle can still rotate considerably. As illustrated in Fig 4(B), the most flexible region in the center part of the binding pocket, which directly contacts with the middle two residues of a pSer-X-X-Phe peptide and middle atoms of compound C1. Utilizing the flexible loop region allows for the polar residues E1698 and R1699 of the β3-α2 connection loop to form a hydrogen bond with backbone atoms of the phosphopeptides and also accommodate ligands with different shapes. For example, the standard deviations for E1698, R1699 and T1700 were especially large when BRCT bound to P13 and C1, followed by concerted motions of N1742 and G1743 in the linker region. Although P13 still can fit into the binding cavity, the two proline residues limit the arrangement of both molecules to optimize the intermolecular interactions. In contrast, C1 was flexible and adopted multiple bound conformations to strengthen its binding affinity, as discussed in the following sections. For the long peptides, F1772, T1773 from the β1'-α1' connection loop and D1692, A1693 from the β3-α2 connection loop fluctuate to adjust the size of the binding cavity. The size change of binding site agrees with our previous molecular dynamics study, where the size of cavity can be characterized by two angles E1698-A1752-E1836 and S1655-A1752-N1774, which can have difference of 10° upon binding of different peptides (S6 Fig) [25]. In summary, BRCT uses the power of loops to alter the shape and size of the binding site to fit various ligands, combined with a rigid region designed to form stable hydrogen bonds with the phosphate group.

### Ligand binding modes and intermolecular interactions computed by MM/PBSA calculations

**Fig 5:**
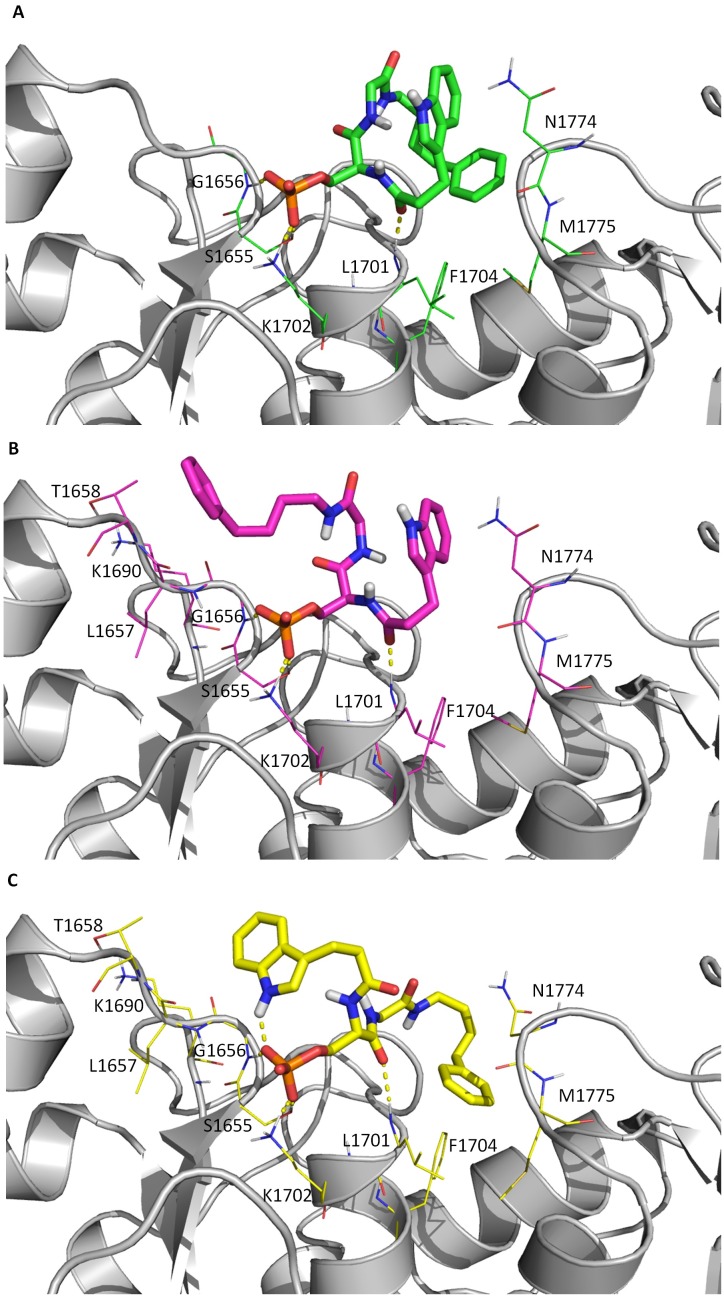
(A, B, C) Three distinct bound conformations of C1 from M2 calculation. Residues of BRCT are shown in line representation and ligand is shown in licorice representation, hydrogen bonds are drawn in dash lines (free energies of A, B and C bound conformations are -1461.16, -1457.04 and -1453.72 kcal/mol, respectively).

Because the BRCT domain has a highly adaptable binding pocket, we hypothesized that some ligands may feature diverse binding modes. We therefore examined the ligand binding modes and the rotamer of each rotatable bond for every ligand to discover their differences between the free and bound states. For all peptides P1–P14, only one major bound conformation was observed: pSer forms hydrogen bonds with S1655, G1656 and K1702 and the P+3 Phe locates in the hydrophobic packet (Fig 1). Interestingly, compound C1 can establish multiple bound conformations in the binding site by fitting either a benzene ring into the hydrophobic pocket and an indole ring into a cluster of residues G1656, L1657, T1658 of the β1-α1 connection loop and K1690 of the β3-α2 connection loop, and vice versa (Fig 5(C) and 5(B)). C1 can also bind to BRCT with its folded form, whereby two rings form a T-shape stacking interaction (Fig 5(A)).

**Fig 6:**
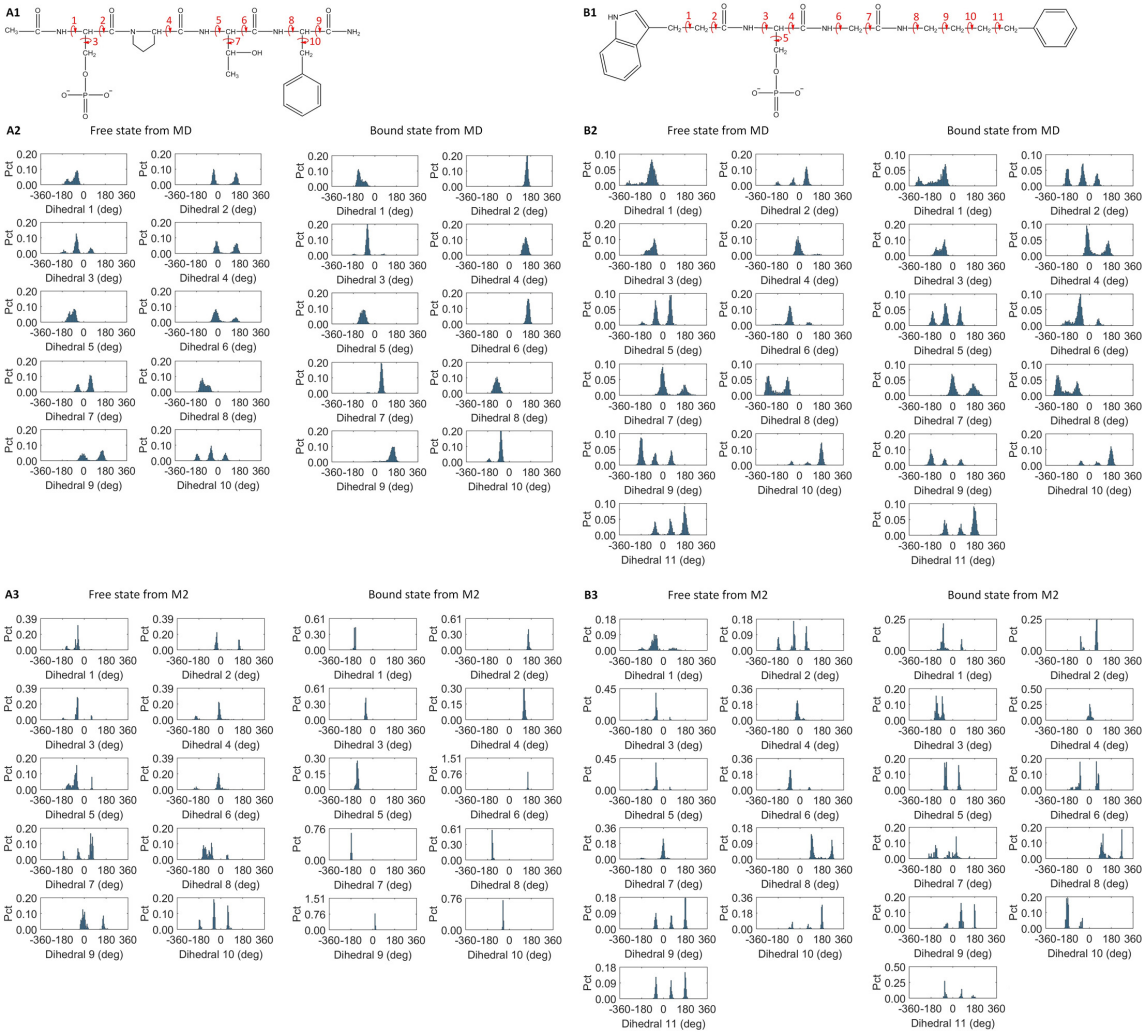
The rotameric states of selected rotatable bonds of P4 and C1 in both free and bound states. (A1), (B1). Selected rotatable bonds of ligand P4 and C1 structures, respectively. (A2), (B2). The dihedral angle distribution from 1000 frames collected during 100-ns MD simulations of P4 and C1, respectively. (A3), (B3). The dihedral angle distribution for distinct energy minima found by M2 calculations of P4 and C1, respectively.

Fig 6 illustrates the rotameric states of selected rotatable bonds of P4 and C1 in their free and bound states. All peptides show the same trend as in the histogram plots of P4, with most rotatable bonds becoming more rigid and losing rotameric states in their bound state (S7 Fig). However, compound C1 does not lose rotamers in the bound state, and a few dihedrals are even more flexible in the bound form. BRCT does not reduce the number of rotamers after binding to C1 either, which differs from the bound states with other peptides (S8 Fig). MM/PBSA calculations suggested that the intermolecular interactions between all the peptides/ligands and BRCT are about the same, which agrees with experiments finding that ΔΔG_exp_ is within 3 kcal/mol (Table 2).

**Table 2:** BRCT domain−ligand Interaction Energy (kcal/mol) of P1–14 and C1, N1 and D1 calculated by molecular mechanics/Poisson-Boltzmann surface area (MM/PBSA).

No.	ΔΔG_exp_	Δ(U+W)	ΔU_VDW_	ΔW_NP_	ΔE_NP_	ΔU_Coul_	ΔW_PB_	ΔE_polar_
P1	0.00	-3.19±0.72^a^	-32.7±0.5	20.8±0.1	-11.9±0.5	-84.6±3.7	93.3±3.67	8.73±0.86
P2	0.28	-2.24±0.66	-31.9±0.5	20.6±0.1	-11.3±0.5	-76.4±3.3	85.4±3.28	9.06±0.56
P3	0.69	-3.48±0.34	-31.0±0.3	20.6±0.1	-10.4±0.4	-160±2	167±2	6.91±0.42
P4	0.91	-4.68±0.50	-32.8±0.2	21.3±0.1	-11.5±0.2	-160±2	167±1	6.79±0.42
P5	1.17	-2.45±0.58	-35.0±0.8	21.5±0.3	-13.4±0.6	-151±3	162±2	11.0±0.5
P6	1.61	-1.53±0.56	-31.3±0.4	21.4±0.2	-9.83±0.25	-163±2	171±2	8.30±0.75
P7	1.61	-2.36±0.72	-28.8±0.8	19.6±0.2	-9.17±0.68	-152±3	159±2	6.81±0.58
P8	1.74	-4.86±0.56	-33.4±0.5	19.9±0.3	-13.5±0.5	-139±2	148±2	8.64±0.38
P9	2.03	-0.53±0.66	-31.5±0.7	20.4±0.1	-11.1±0.6	-150±4	161±3	10.6±1.0
P10	2.12	-1.06±1.35	-30.5±0.5	20.0±0.2	-10.4±0.4	-142±12	151±11	9.38±1.37
P11	2.36	-0.50±0.85	-32.3±0.4	21.6±0.1	-10.6±0.3	-122±3	132±3	10.1±0.7
P12	2.74	-3.15±0.46	-30.9±0.4	19.8±0.2	-11.0±0.3	-152±2	160±2	7.89±0.48
P13	3.29	-3.05±0.41	-29.3±0.2	18.5±0.1	-10.8±0.2	-123±2	130±2	7.74±0.50
P14	3.29	0.39±0.99	-29.4±0.6	20.8±0.2	-8.61±0.43	-149±2	158±2	9.00±1.14
C1	-0.70	-1.59±0.66	-18.9±1.7	15.3±0.7	-3.55±0.99	-134±3	136±4	1.96±1.02
N1	3.29	-3.05±0.70	-29.8±0.5	18.6±0.2	-11.2±0.5	-59.4±5.4	67.6±4.9	8.12±0.68
D1	N/A	0.21±1.82	-33.0±1.8	21.0±0.7	-12.0±1.2	-121±9	133±7	12.2±2.7

The binding interaction energy was computed by ΔE_cal_ = E_complex_-E_free protein_-E_free ligand_. Decomposed interaction energy, E_cal_, from our calculations includes Lennard-Jones energy <U_VDW_>, nonpolar solvation free energy <W_NP_>, Coulombic energy <U_Cou_l>, and PB solvation free energy <W_PB_>. <E_np_> represents the sum of <U_VDW_> and <W_NP_>; <E_ploar_> represents the sum of <U_Coul_> and <W_PB_>, bonded terms <U_val_> are zero due to energy cancelling out and therefore not listed here.

^*a*^ The statistical error was estimated on the basis of the deviation between block averages [82].

**Fig 7:**
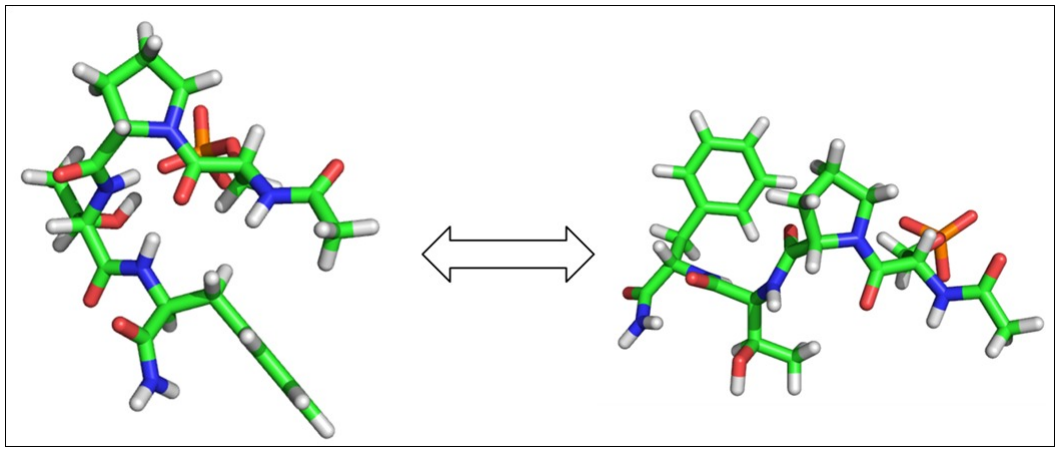
Conformational change of P4 between bent and stretched in free ligand state.

To understand why or why not a ligand loses the rotamers after binding, we clustered conformations of the free peptides and ligands and compared them with those in the bound complexes. For the peptides and C1, they generally have two distinct conformations in the free state, folded and extended, which except for P13 (Ac-pSPPF-NH_2_), can switch back and forth in MD simulations of free ligands (Fig 7). However, the bound peptides are locked to only the extended form, which results in reduced rotamers in side-chains and also backbone φ and ψ angles (Figs 6 and S7). To test the robustness of MD simulation on rotameric states analysis, we ran and analyzed another MD run with different initial conformations for several ligands. The simulated rotameric states are nearly identical to the other MD, showing that multiple rotameric states in free states reduce to single rotameric state in bound state (S9 Fig). For C1, both folded and extended forms are observable in the bound states; free energy calculations with M2 further revealed that all these distinct ligand conformations are stable energy minima (Fig 5).

### Binding free energies with M2 method

To gain insights into the mechanism of binding, we needed thorough sampling and accurate ligand binding free energy calculations that included both enthalpic and configurational entropic contributions for molecular recognition. Although MM/PBSA calculations provide valuable information for intermolecular interactions, our calculations based on 100-ns MD simulations may have missed some important conformations, and contributions from changing configurational entropy and molecular conformations are neglected in Table 2. In addition, because of different non-polar solvation models and use of a real set in M2 for energy calculations (Fig 3), the values of non-polar and polar interaction energies, ΔE_NP_ and ΔE_Polar_, from MM/PBSA and M2 cannot be compared directly. We therefore computed ligand-binding free energy with the M2 method, which involved an aggressive conformational search engine to locate local energy minima and a rigorous modified harmonic approximation approach to compute free energy for each minimum found.

**Fig 8:**
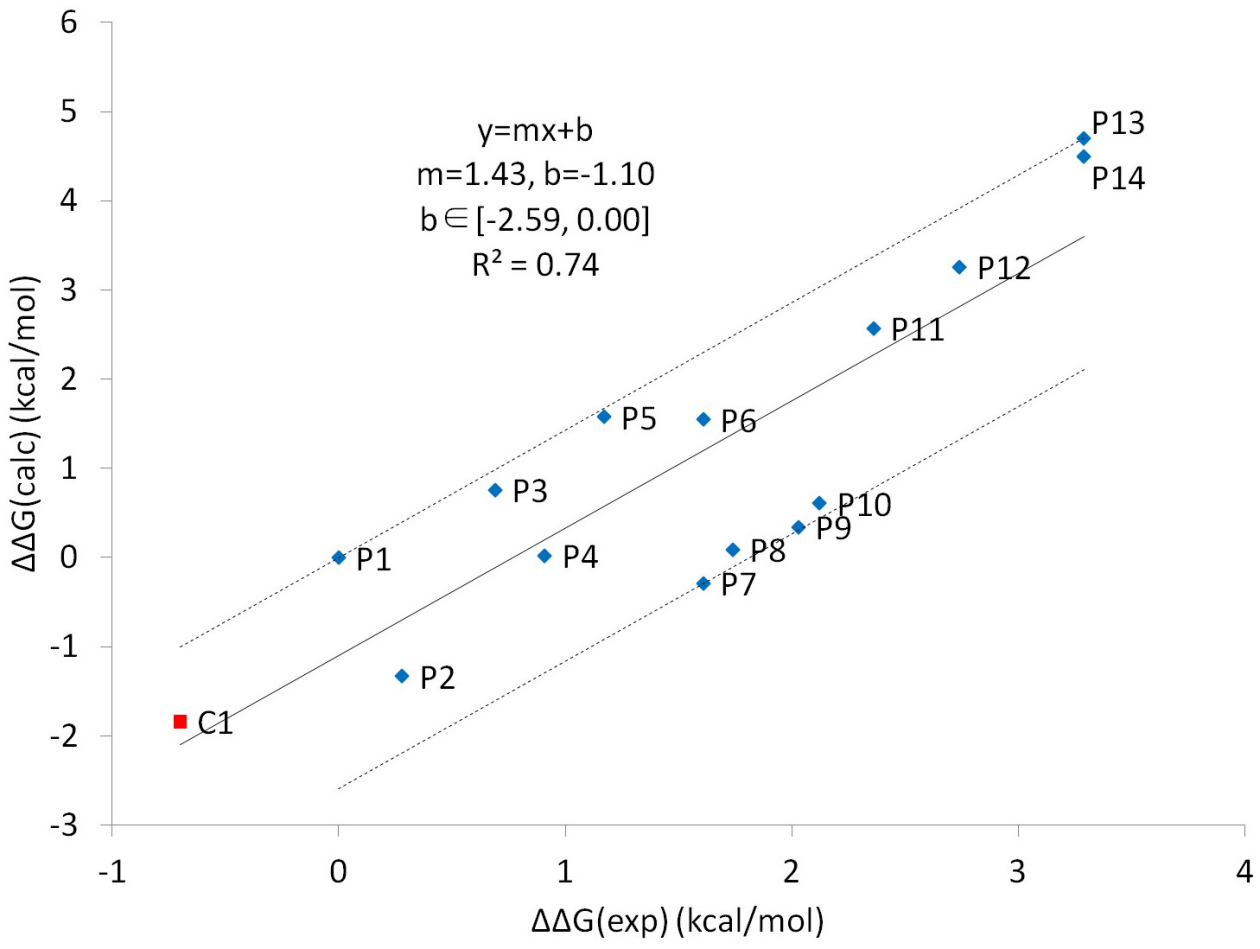
Calculated versus experimental relative binding free energies ΔΔG (kcal/mol) for P1–P14 and C1.

Table 3 and Fig 8 show that the computed related binding free energy, ΔΔG_calc_, was in good agreement with experimental values, which validated the method as well. Because M2 uses accumulated energy which is different from dynamics-based method, it does not have fluctuated energy. Eq 2 shows that when a low energy minimum is found by M2 conformational search and added to the accumulated energy, the computed free energy G° drops. Search and computation continue until the accumulated free energy is converged. Here we calculated error interval for y-intercept of linear regression line in Fig 8 [83–85]. Part of the variance comes from experimental noise, which is typically about 0.3–0.5 kcal/mol for accurate binding free energy measurements [86]. If the binding free energies of two ligands are measured independently in experiments, then experimental relative binding free energies between the two ligands would have error around 0.4–0.7 kcal/mol. Therefore, the errors for free energy calculation method versus experimental data can only be larger than experimental noise of 0.4–0.7 kcal/mol, indicated by the range of y-intercept of linear regression line (~3 kcal/mol), and experimental noise is expected to be a at least 13% of the total observed error. With agreement of early studies on ligand–protein binding, the strong Coulombic attraction is largely compensated by the solvation free energy, and the vdW attraction is the major driving force for ligand binding [32, 64]. Moreover, peptides with large non-polar residues at the P+2 position, such as P2, P3, P5, P8, P10 and P12, generally have stronger vdW interaction (Table 3). Although M2 revealed more bound conformations for the complex from various combinations of side-chain rotations, the major binding mode of BRCT-pSXXF is the same as that obtained by MD sampling, whereby the phosphate group forms hydrogen bonds with S1655, G1656 and K1702, and P+3 Phe or Tyr locates in the hydrophobic pocket (S10 Fig). M2 also revealed more conformations for free ligands, including the folded and extended forms, and their computed conformational free energies are similar. Therefore, the folded and extended conformations may have similar population in the free ligands.

**Table 3:** Binding free energy, average binding potential energy, and solvation free energy (kcal/mol) of P1–14, C1, N1 and D1 calculated by M2.

No.	ΔΔG_exp_	ΔG_cal_	ΔΔG_calc_	Δ(U+W)	-TΔS	ΔU_Val_	ΔU_VDW_	ΔW_NP_	ΔE_NP_	ΔU_Coul_	ΔW_PB_	ΔE_polar_
P1	0.00	-10.5	0.00	-42.4	31.9	5.08	-33.2	-4.40	-37.6	-291	281	-9.90
P2	0.28	-11.9	-1.33	-42.3	30.4	-0.80	-37.6	-4.35	-42.0	-274	275	0.49
P3	0.69	-9.76	0.76	-39.1	29.3	0.98	-39.7	-4.28	-44.0	-245	249	3.93
P4	0.91	-10.5	0.02	-37.9	27.4	0.20	-34.9	-4.03	-38.9	-242	243	0.84
P5	1.17	-8.94	1.58	-37.8	28.9	-1.28	-38.6	-4.22	-42.8	-238	244	6.32
P6	1.61	-8.97	1.55	-38.7	29.7	-1.10	-31.8	-4.02	-35.8	-243	241	-1.82
P7	1.61	-10.8	-0.29	-37.6	26.8	-3.61	-29.7	-3.80	-33.5	-249	249	-0.53
P8	1.74	-10.4	0.09	-37.5	27.1	0.48	-37.4	-4.11	-41.5	-239	242	3.61
P9	2.03	-10.2	0.34	-37.7	27.5	0.80	-38.3	-3.98	-42.2	-234	238	3.73
P10	2.12	-9.91	0.61	-39.5	29.6	-2.30	-37.5	-4.08	-41.6	-227	231	4.44
P11	2.36	-7.95	2.57	-35.7	27.8	-1.62	-29.0	-4.03	-33.0	-248	247	-1.05
P12	2.74	-7.26	3.26	-35.3	28.0	-2.09	-36.3	-3.69	-40.0	-217	224	6.84
P13	3.29	-5.82	4.70	-34.6	28.8	-2.62	-35.7	-4.22	-40.0	-231	239	8.01
P14	3.29	-6.02	4.50	-36.3	30.3	-5.33	-38.6	-4.21	-42.8	-227	239	11.8
C1	-0.70	-12.4	-1.84	-38.0	25.6	-4.17	-30.9	-3.91	-34.9	-212	213	1.04
N1	3.29	-7.60	2.90	-37.7	30.1	-0.74	-33.4	-4.41	-37.9	-270	271	0.886
D1	N/A	-14.4	-3.84	-40.9	26.5	-2.70	-30.6	-3.92	-34.5	-219	215	-3.69

The binding free energy was computed by ΔG_cal_ = G_complex_-G_free protein_-G_free ligand_. Each decomposed energy is obtained by ΔE_cal_ = E_complex_-E_free protein_-E_free ligand_. Decomposed free energy, G_cal_, from our calculations includes the average potential energy <U+W>, configurational entropy -TS, bonded terms <U_val_>, Lennard-Jones energy <U_VDW_>, nonpolar solvation free energy <W_NP_>, Coulombic energy <U_Cou_l>, and PB solvation free energy <W_PB_>. <E_np_> represents the sum of <U_VDW_> and <W_NP_>; <E_ploar_> represents the sum of <U_Coul_> and <W_PB_>.

**Fig 9:**
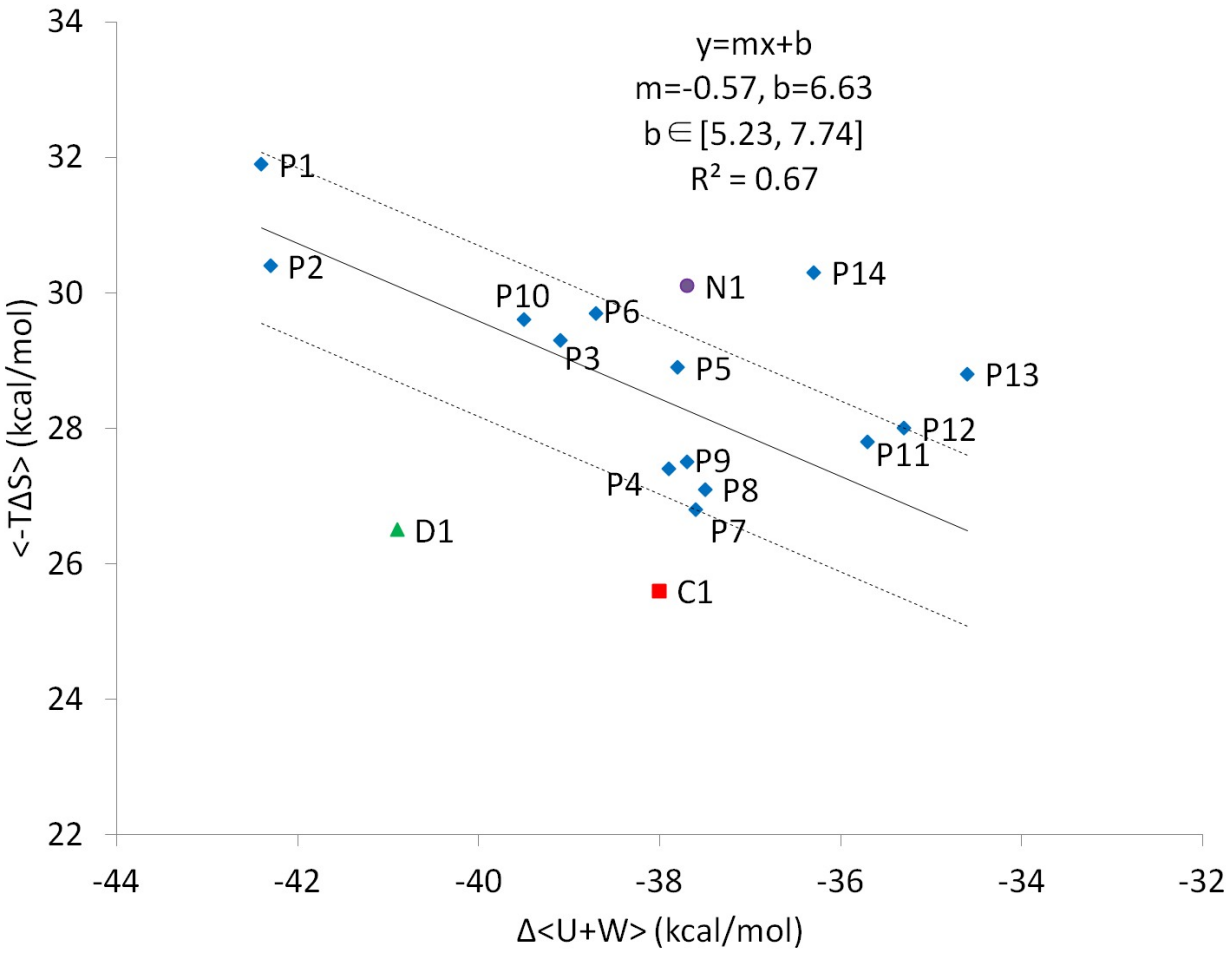
Computed configurational entropy contribution, <-*T*Δ*S*> and energy contribution, Δ*<U+W>*, for P1–14 and C1, N1 and D1. <-*T*Δ*S*> vs Δ*<U+W>* is plotted using tight peptide binders P1–P12.

It is not surprising to observe the enthalpy Δ<U+W> and configuration entropy–TΔ<S> compensation for tight binders; however, the outlier C1 is particularly of interest (Fig 9). Although C1 forms a moderate enthalpy attraction with BRCT, ~ -38 kcal/mol, which is similar to that for peptides P4–P9, with the remarkable ~2–4 kcal/mol small configuration entropy loss, C1 outperforms other peptides (Table 3). Compared with peptides, some rotamers of BRCT and C1 can gain new rotameric states rather than losing them, and the vibrational entropy loss is smaller than that for P4–P9, as seen from the change in width of M2 histogram peaks that correspond to the width of energy wells (Figs 6 and S7). Upon ligand binding, M2 histogram peaks for P1–P14 become narrower, whereas C1 has the same or even wider peaks. In S2 Table, we list the number of complex, ligand and protein conformations from M2 calculations. For example, M2 calculations generated 482 distinct conformations of free P1 within 10 RT of the most stable free conformation. Even if free P1 were equally stable in all 482 energy wells with only one bound conformation, the maximum change in conformational entropy would only be reduced by RTln 482 = ~3.7 kcal/mol, which is significantly smaller than the -TΔS values in Table 3. We may approximate vibrational entropy through -TΔS_vib_ = -TΔS_config_ + TΔS_conf_. S3 Table shows that C1 has much smaller vibrational entropy loss than peptides P1–P14. In sum, both conformational entropy and vibrational entropy are attributed to the smaller configuration entropy loss of C1. Interestingly, P7, with a small residue alanine in the P+1 position, has the second smallest entropic penalty in M2 results but not P13, which has two proline residues in the middle of the peptide. P13 managed to partially eliminate the folded conformations because of the geometric constraint proline residues; however, the entropy cost does not decrease substantially due to the big vibrational entropy loss (S7 Fig and S3 Table). Moreover, the restraint by the two prolines resulted in the incorrect orientation of ligand-bound conformations, which significantly weakens the polar attractions (S11 Fig).

### Inhibitor design: New strategy for promiscuous modular domains?

Two strategies are commonly used in ligand design for enhancing binding affinities: increasing intermolecular attractions and decreasing entropy loss upon binding. For example, new interactions between ligands and receptors, such as adding hydrogen bonds, can be introduced to increase enthalpic attractions [87–93]. The other way is via reducing the entropy cost by pre-rigidifying the ligand to its bound conformation [94, 95]. This pre-organization of the ligand to its bound conformation lessens the decrease in number of rotameric states, and thus affinity is increased primarily because of optimizing the entropic term.

Because the number of potential hydrogen bonds may already be maximized by the presence of the phosphate group, we used the latter strategy to pre-organize a ligand by introducing a benzene ring in the ligand backbone to limit its conformational flexibility. Having a benzene ring in the middle at a certain level prevents the ligand from bending and forming intra-molecular hydrogen bonds like other tetrapeptides do. A new ligand, N1, was synthesized (Fig 2) and its binding to BRCT was tested experimentally. Although the conformations were constrained to some degree to reduce conformational entropy penalty (S7 Fig), the loss from the vibrational part was not reduced enough. The conformational constraints by the benzene ring restricted the ligand rearrangement to optimize the polar and non-polar contacts to the protein, thereby resulting in weak binding (Table 3 and S12 Fig). N1 performed similar to P13, so over-rigidifying a ligand is not advantageous, which suggests the challenge in retaining optimized intermolecular interactions in pre-rigidifying a peptidomimic compound. Previous work in design of potent Cbl(TKB)-binding peptides drew the same conclusion [96]. Therefore, because of conformational flexibility at the binding interface of a modular domain, flexible ligands may be favorable.

Another strategy to lower entropy penalty, although less common, is by introducing a less rigid complex while the molecules bind. Because the strategies to further modify the short peptides to increase their bound conformations may be exhausted, compounds with phosphate groups are a better alternative. On the basis of our calculations and the structure of compound C1, we further modified it to enhance intermolecular attractions by the formation of additional hydrogen bonds between the ligand and BRCT. In the meantime, we kept the template structure intact to maintain its flexibility. We added one hydroxyl group to the para site of the benzene ring of C1, which can form hydrogen bonds with K1690 or N1774 with different bound conformations (S13 Fig). Therefore, designed compound D1 shows improved binding affinity, by 2 kcal/mol, with more negative Δ(U+W) as compared with C1 (Fig 9 and Table 3). As compared with C1, D1 has a stronger Coulombic interaction with BRCT because of the additional hydogen bonds (Table 3). Moreover, because D1 can also adopt mutiple distinct bound conformations, the entropy cost is minimal, as is found in C1. The enthalpy-entropy compensation plot shown in Fig 9 clearly indicates that D1 outperforms other peptides by both increasing intermolecular attraction and reducing entropic penalty.

In summary, designing a pre-rigidified ligand to reduce entropy cost can be tricky considering the potential loss of intermolecular attraction due to lack of proper rearrangement in the bound state. Fortunately, making a ligand more flexible and able to retain its plasticity in the bound conformation provides an effective strategy to reduce entropy cost, while the optimization of interactions between such a flexible ligand and a target protein can further improve binding affinity. Although for designing tight binders such as many drug-protein binding systems, pre-rigidified may still be the best strategy, our study points out a new direction for designing inhibitors targeting promiscuous modular domains and PPIs.

### Supporting Information available

Supplemental figures, data, results and detailed experimental method are provided in Supporting Information. All files, including the input and output files for MD simulations, post-analysis and M2 methods, MD trajectories, and results from various energy calculations are freely available upon request (email: chiaenc@ucr.edu).

## Supporting Information

S1 Table Sources of initial bound conformations of ligands for MD simulation. (DOCX)

S2 Table Numbers of complex, free ligand and protein conformations from M2 calculation. M2 uses a rigorous conformational search through dihedral distortion for new conformations. Molecular torsional modes are calculated via diagonalization of matrix of energy 2nd-derivatives transformed into internal coordinates with all bond and angle rows and columns removed. After a complete distortion along these modes, the whole system is energy minimized via a quasi-Newton geometry optimization to get new conformations. 1RT means numbers of conformations within 1RT above the global energy minimum.
(DOCX)

S3 Table Approximated conformational and vibrational entropy (kcal/mol) for P1–P14, C1, N1 and D1. The conformational entropy penalty is approximated through RTln *M* (*M* is the number of conformations within 10RT of most stable free ligand conformation from S2 Table). The vibrational entropy penalty was computed by -TΔS_vib_ = -TΔS_config_ + TΔS_conf_. ^a^ For C1 and D1, they have at least three distinct bound conformations (Figs 5 and S13), so the conformational entropy penalty of C1 and D1 is approximated through RTln (*M*/3).
(DOCX)

S1 Fig Two initial bound structures of C1 from docking. The trajectory that covers the conformations close to the three bound structures in M2 search (Fig 5) was further used for MM/PBSA calculation.
(TIF)

S2 Fig Convergence plots for cumulated free energy of complex BRCT and P1/P2. (TIF)

S3 Fig Mass spectrum of N1. (TIF)

S4 Fig Representative dose-response curves from an fluorescence polarization assay study that were used to determine the IC50 values shown in Table 1 (1 = P4; 3 = P11; 6 = P13; 7 = P10; 8 = P7). (TIF)

S5 Fig Flexibility of active site of BRCT. (A). Root mean square fluctuation (RMSF) of C_α_ of the residues of the receptor within 7 Å of ligands during MD simulations. (B). Flexible region of the active site. Flexible residues of the protein are shown in a green line representation. Ligand is shown as a blue tube with pSer and Phe (P+3) residues in licorice representation.
(TIF)

S6 Fig Angles E1698-A1752-E1836 and S1655-A1752-N1774 as indications of size change of BRCT binding site. (TIF)

S7 Fig Ligand P1–P3, P5–P14, N1 and D1 structures with selected rotatable bonds and dihedral angle distributions from MD and M2. (DOCX)

S8 Fig Comparison of the first side-chain dihedral angles of part of live set residues of C1 and P4-BRCT complex from MD and M2, respectively. (A1), (A2). The first side-chain dihedral angles of part of live set residues of C1 and P4-BRCT complexes from MD, respectively. (B1), (B2). The first side-chain dihedral angles of part of live set residues of C1 and P4-BRCT complexes from M2, respectively. The difference is highlighted by red circle.
(DOCX)

S9 Fig Representative robustness test of MD simulations on rotameric states analysis with P4. (TIF)

S10 Fig Superimposed most stable bound conformaitons of peptide (P1 to P14). The phosphate groups and phosphate mimic are anchored by S1655, G1656, K1702 and phenylalanine/tyrosine surrounded by F1704, N1774, M1775. Ligands are shown in licorice representation, residues of BRCT are shown in line representation.
(TIF)

S11 Fig Superimposed average bound conformation of P4 (blue) and P13 (yellow) during MD simulations. The bound conformation of P4 is represents the standard bound conformation of most phosphopeptides. Changes in the bound conformation start showing up right after the mutation at the P+2 position from threonine or valine to proline. In P13, in order to align phosphate group and benzene ring of phenylalanine, the whole backbone frame of the ligand has to move towards solvent to moderate the restrain from two rigid proline residues in the middle, which causes the improper fit of P13 in the cavity, resulting in increased enthalpy change and high entropy cost.
(TIF)

S12 Fig Superimposed most stable bound conformations of of P4 (blue) and N1 (pink) from M2 calculation. Ligands are shown in licorice representation, residues of BRCT are shown in line representation. Hydrogen bonds are drawn in dash lines.
(TIF)

S13 Fig (A, B, C) Three distinct bound conformations of D1 from M2 calculations. Residues of BRCT are shown in line mode and the ligand is shown in licorice mode, hydrogen bonds are drawn in dash lines (free energies of A, B and C bound conformations are -1476.72, -1476.28 and -1465.36 kcal/mol, respectively).
(TIF)
