# Supplementary material for: Characterization of Promiscuous Binding of Phosphor Ligands to Breast-Cancer-Gene 1 (BRCA1) C-Terminal (BRCT): Molecular Dynamics, Free Energy, Entropy and Inhibitor Design
Source: PLoS Comput Biol. 2016 Aug 25;12(8):e1005057. doi: 10.1371/journal.pcbi.1005057 (PMC4999267; doi:10.1371/journal.pcbi.1005057)
Supplement: S1 Table — (DOCX) [file pcbi.1005057.s001.docx]

**S1 Table. Sources of initial bound conformations of ligands for MD simulation.**

| L1 | Crystal structure 1T29 |
| --- | --- |
| L2 | Crystal structure 1Y98 |
| L3 | Crystal structure 1T2V |
| L4 | Crystal structure 3COJ |
| P1-P14 | pSXXF sequence superimposed to crystal structure 1T29 |
| C1, N1, D1 | Docked to crystal structure 1T29 |
