# Supplementary material for: Characterization of Promiscuous Binding of Phosphor Ligands to Breast-Cancer-Gene 1 (BRCA1) C-Terminal (BRCT): Molecular Dynamics, Free Energy, Entropy and Inhibitor Design
Source: PLoS Comput Biol. 2016 Aug 25;12(8):e1005057. doi: 10.1371/journal.pcbi.1005057 (PMC4999267; doi:10.1371/journal.pcbi.1005057)
Supplement: S2 Table — M2 uses a rigorous conformational search through dihedral distortion for new conformations. Molecular torsional modes are calculated via diagonalization of matrix of energy 2nd-derivatives transformed into internal coordinates with all bond and angle rows and columns removed. After a complete distortion along these modes, the whole system is energy minimized via a quasi-Newton geometry optimization to get new conformations. 1RT means numbers of conformations within 1RT above the global energy minimum. (DOCX) [file pcbi.1005057.s002.docx]

**S2 Table. Numbers of complex, free ligand and protein conformations from M2 calculation.**

| **No.** | Numbers of complex conformations in M2 | | | | | Numbers of free ligand conformations in M2 | | | | |
| --- | --- | --- | --- | --- | --- | --- | --- | --- | --- | --- |
|  | Total | 1RT | 3RT | 5RT | 10RT | Total | 1RT | 3RT | 5RT | 10RT |
| P1 | 864 | 3 | 28 | 54 | 181 | 824 | 3 | 61 | 171 | 482 |
| P2 | 1086 | 9 | 31 | 64 | 216 | 373 | 3 | 20 | 64 | 200 |
| P3 | 581 | 3 | 25 | 58 | 165 | 589 | 26 | 99 | 163 | 370 |
| P4 | 661 | 4 | 35 | 65 | 171 | 507 | 6 | 37 | 99 | 323 |
| P5 | 755 | 3 | 20 | 36 | 133 | 990 | 29 | 142 | 279 | 663 |
| P6 | 727 | 4 | 25 | 55 | 153 | 388 | 2 | 37 | 96 | 286 |
| P7 | 581 | 2 | 24 | 58 | 165 | 481 | 12 | 54 | 126 | 355 |
| P8 | 538 | 2 | 21 | 41 | 87 | 957 | 10 | 70 | 199 | 627 |
| P9 | 535 | 4 | 29 | 50 | 140 | 470 | 8 | 46 | 126 | 316 |
| P10 | 501 | 5 | 23 | 54 | 148 | 563 | 1 | 49 | 130 | 338 |
| P11 | 764 | 3 | 17 | 51 | 176 | 747 | 6 | 62 | 171 | 488 |
| P12 | 594 | 3 | 16 | 53 | 216 | 408 | 3 | 30 | 98 | 263 |
| P13 | 362 | 1 | 12 | 28 | 125 | 448 | 28 | 92 | 148 | 284 |
| P14 | 363 | 3 | 19 | 34 | 145 | 328 | 9 | 47 | 94 | 254 |
| C1 | 1992 | 5 | 41 | 80 | 301 | 1119 | 5 | 41 | 148 | 673 |
| N1 | 367 | 4 | 6 | 17 | 60 | 393 | 9 | 49 | 91 | 233 |
| D1 | 1472 | 2 | 18 | 51 | 229 | 660 | 2 | 23 | 90 | 386 |
| protein | 802 | 8 | 47 | 128 | 409 |  |  |  |  |  |

M2 uses a rigorous conformational search through dihedral distortion for new conformations. Molecular torsional modes are calculated via diagonalization of matrix of energy 2nd-derivatives transformed into internal coordinates with all bond and angle rows and columns removed. After a complete distortion along these modes, the whole system is energy minimized via a quasi-Newton geometry optimization to get new conformations. 1RT means numbers of conformations within 1RT above the global energy minimum.
