# Supplementary material for: Characterization of Promiscuous Binding of Phosphor Ligands to Breast-Cancer-Gene 1 (BRCA1) C-Terminal (BRCT): Molecular Dynamics, Free Energy, Entropy and Inhibitor Design
Source: PLoS Comput Biol. 2016 Aug 25;12(8):e1005057. doi: 10.1371/journal.pcbi.1005057 (PMC4999267; doi:10.1371/journal.pcbi.1005057)
Supplement: S3 Table — The conformational entropy penalty is approximated through RTln M (M is the number of conformations within 10RT of most stable free ligand conformation from S2 Table). The vibrational entropy penalty was computed by -TΔSvib = -TΔSconfig + TΔSconf. a For C1 and D1, they have at least three distinct bound conformations (Figs 5 and S13), so the conformational entropy penalty of C1 and D1 is approximated through RTln (M/3). (DOCX) [file pcbi.1005057.s003.docx]

**S3 Table. Approximated conformational and vibrational entropy (kcal/mol) for P1-P14, C1, N1 and D1.**

| **No.** | -TΔS_config_ | -TΔS_conf_ | - TΔS_vib_ |
| --- | --- | --- | --- |
| P1 | 30.1 | 3.68 | 26.5 |
| P2 | 28.6 | 3.16 | 25.5 |
| P3 | 27.5 | 3.53 | 24.0 |
| P4 | 25.6 | 3.44 | 22.2 |
| P5 | 27.0 | 3.87 | 23.2 |
| P6 | 28.0 | 3.37 | 24.6 |
| P7 | 25.0 | 3.50 | 21.5 |
| P8 | 25.2 | 3.84 | 21.4 |
| P9 | 25.7 | 3.43 | 22.3 |
| P10 | 27.8 | 3.47 | 24.3 |
| P11 | 25.9 | 3.69 | 22.2 |
| P12 | 26.2 | 3.32 | 22.9 |
| P13 | 27.0 | 3.37 | 23.6 |
| P14 | 28.5 | 3.30 | 25.2 |
| C1 | 23.8 | 3.88^a^ | 20.0 |
| N1 | 28.3 | 3.25 | 25.1 |
| D1 | 24.8 | 3.55^a^ | 21.2 |

The conformational entropy penalty is approximated through RTln *M* (*M* is the number of conformations within 10RT of most stable free ligand conformation from Table S2). The vibrational entropy penalty was computed by -T∆S_vib_=-T∆S_config_ + T∆S_conf_.

^a^ For C1 and D1, they have at least three distinct bound conformations (Fig 5 and S13), so the conformational entropy penalty of C1 and D1 is approximated through RTln (*M*/3).
