# Supplementary figures and images for: Characterization of Promiscuous Binding of Phosphor Ligands to Breast-Cancer-Gene 1 (BRCA1) C-Terminal (BRCT): Molecular Dynamics, Free Energy, Entropy and Inhibitor Design
Source: PLoS Comput Biol. 2016 Aug 25;12(8):e1005057. doi: 10.1371/journal.pcbi.1005057 (PMC4999267; doi:10.1371/journal.pcbi.1005057)

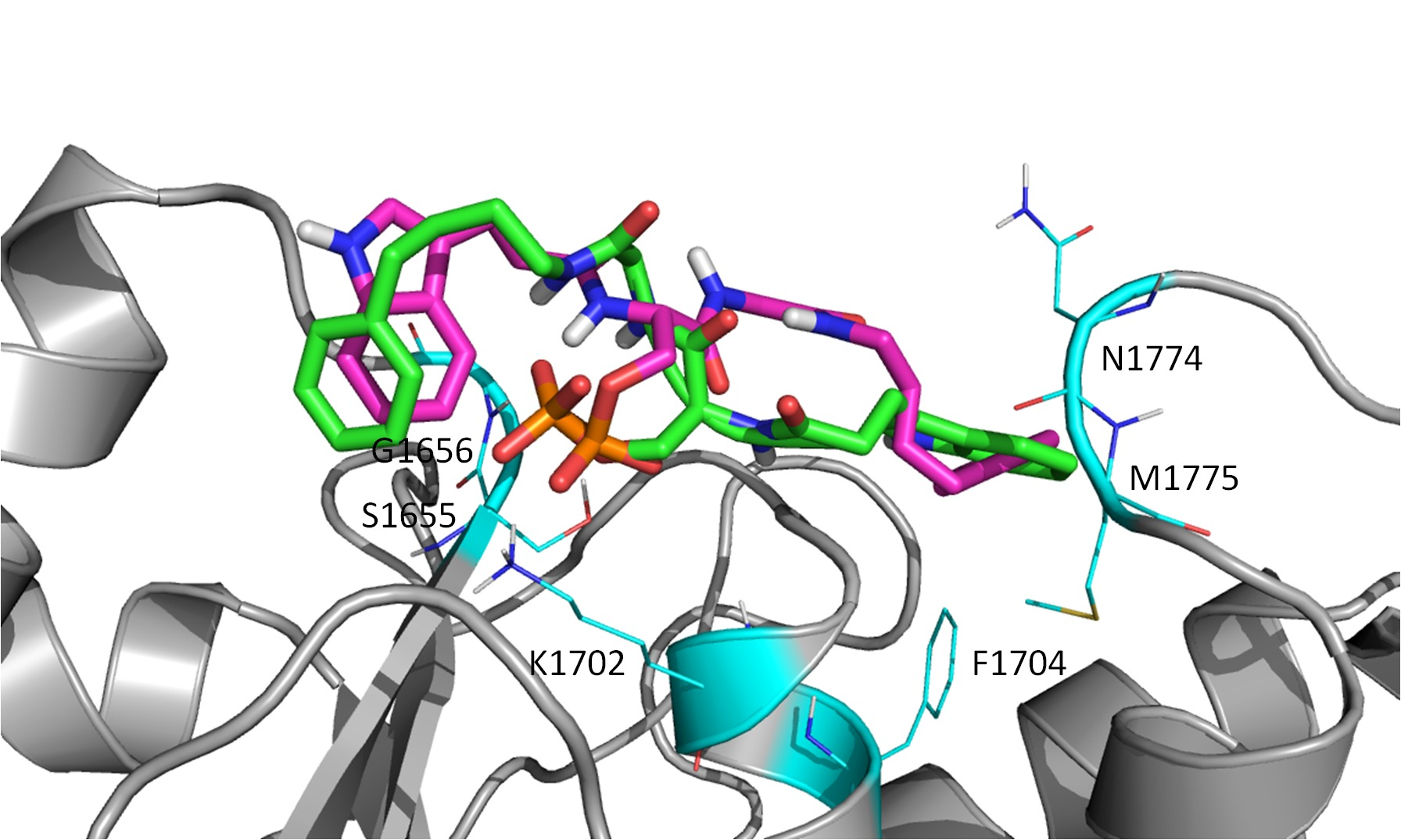

Supplement: S1 Fig — The trajectory that covers the conformations close to the three bound structures in M2 search (Fig 5) was further used for MM/PBSA calculation. (TIF) [file pcbi.1005057.s004.tif]

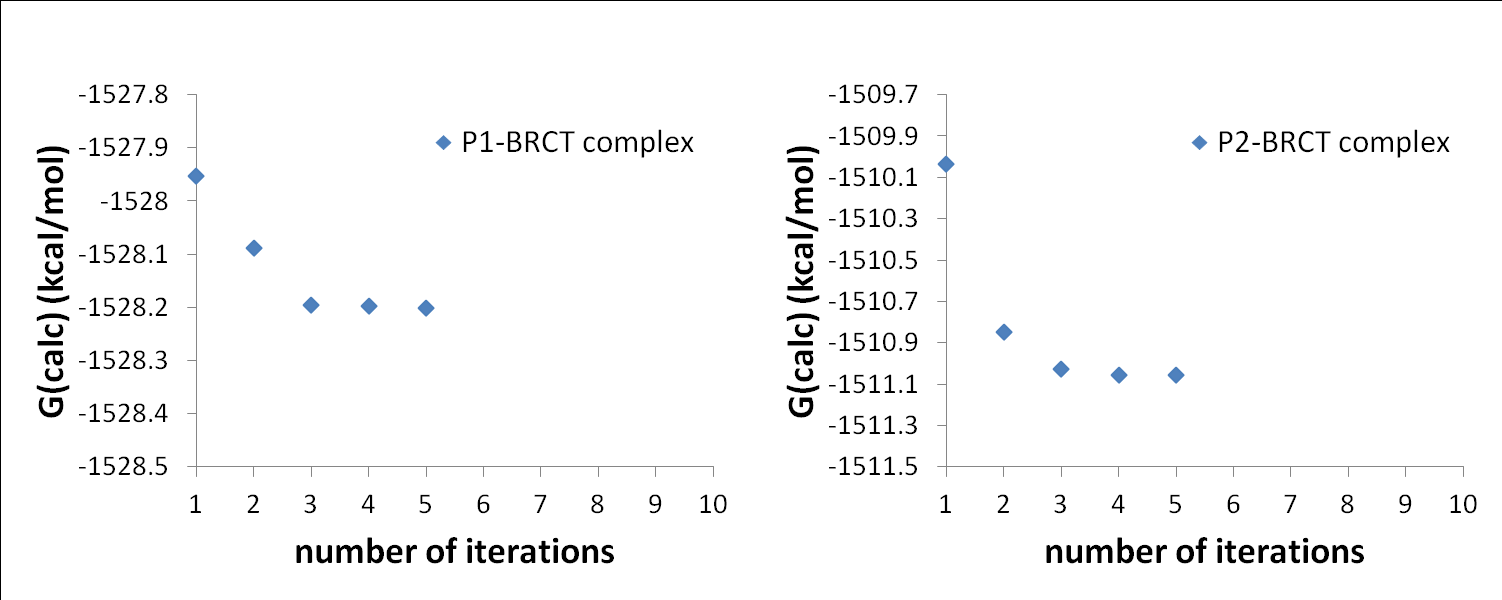

Supplement: S2 Fig — (TIF) [file pcbi.1005057.s005.tif]

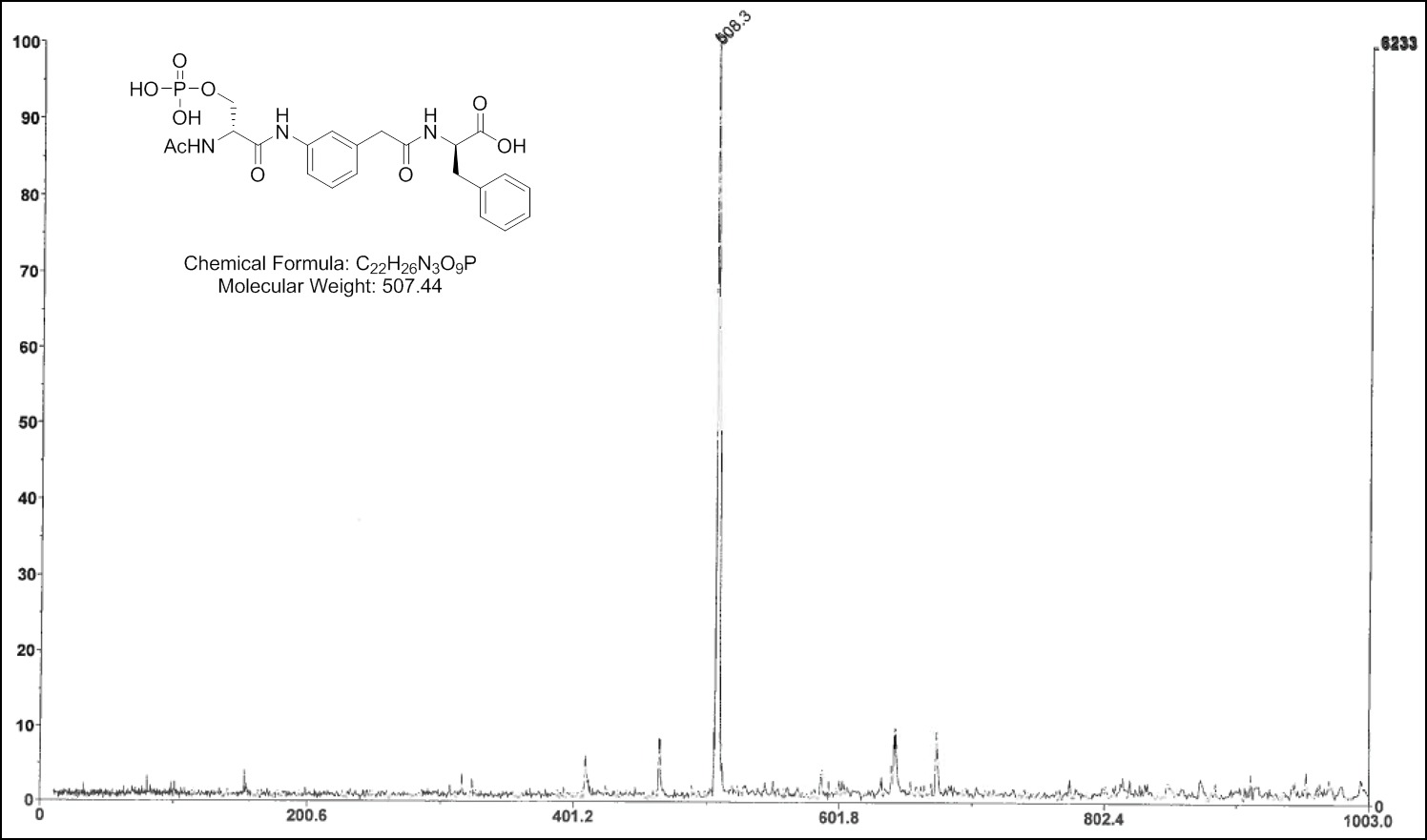

Supplement: S3 Fig — (TIF) [file pcbi.1005057.s006.tif]

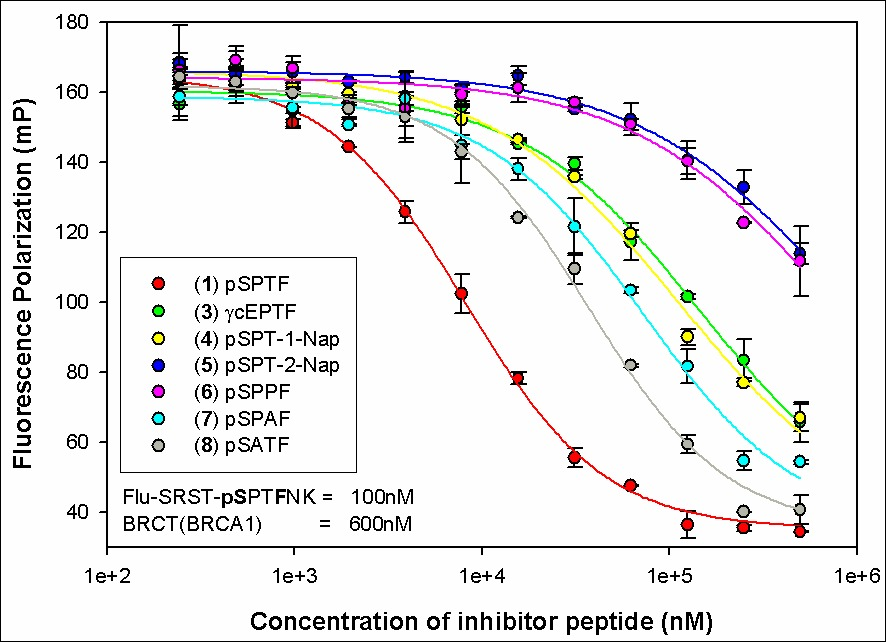

Supplement: S4 Fig — (TIF) [file pcbi.1005057.s007.tif]

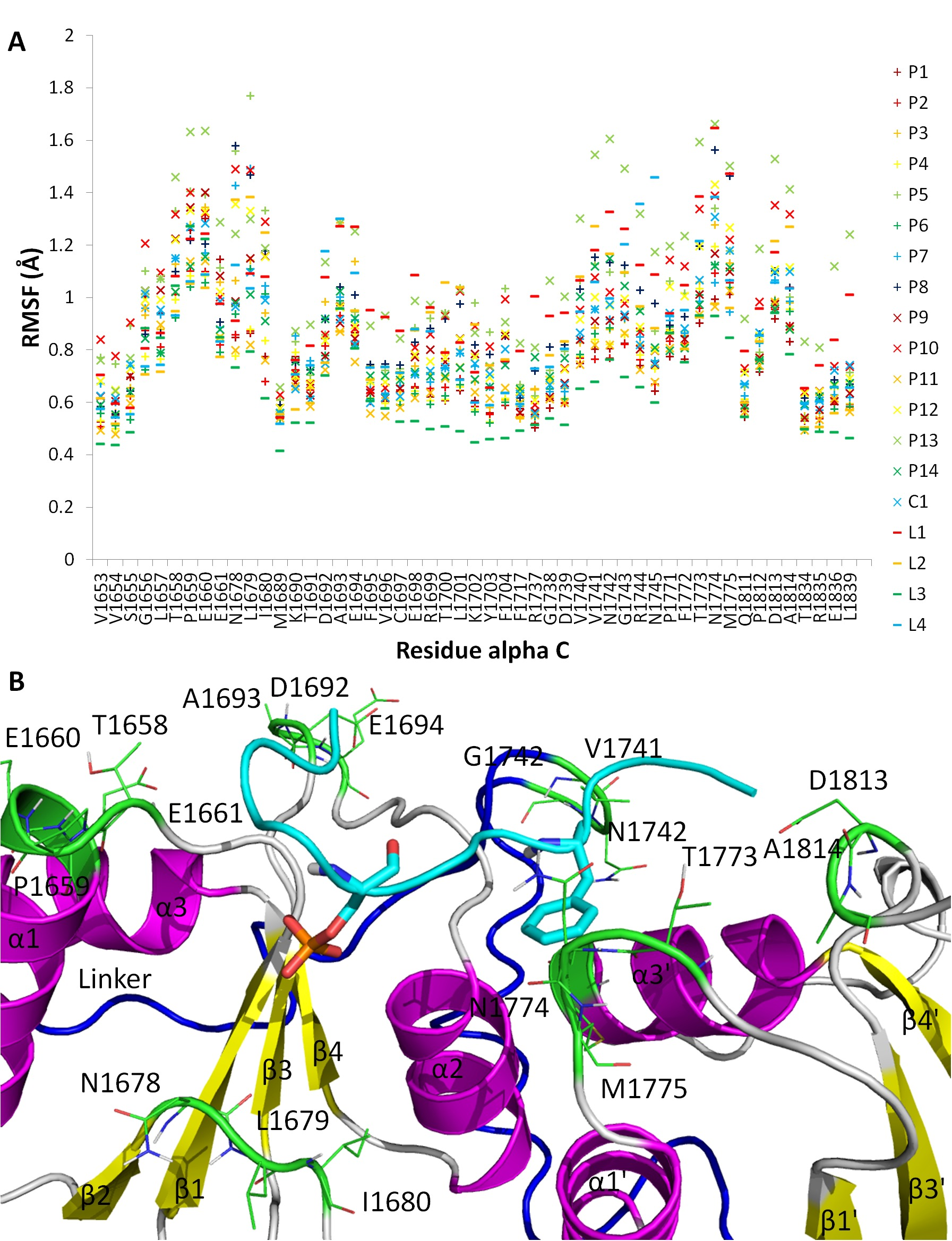

Supplement: S5 Fig — (A). Root mean square fluctuation (RMSF) of Cα of the residues of the receptor within 7 Å of ligands during MD simulations. (B). Flexible region of the active site. Flexible residues of the protein are shown in a green line representation. Ligand is shown as a blue tube with pSer and Phe (P+3) residues in licorice representation. (TIF) [file pcbi.1005057.s008.tif]

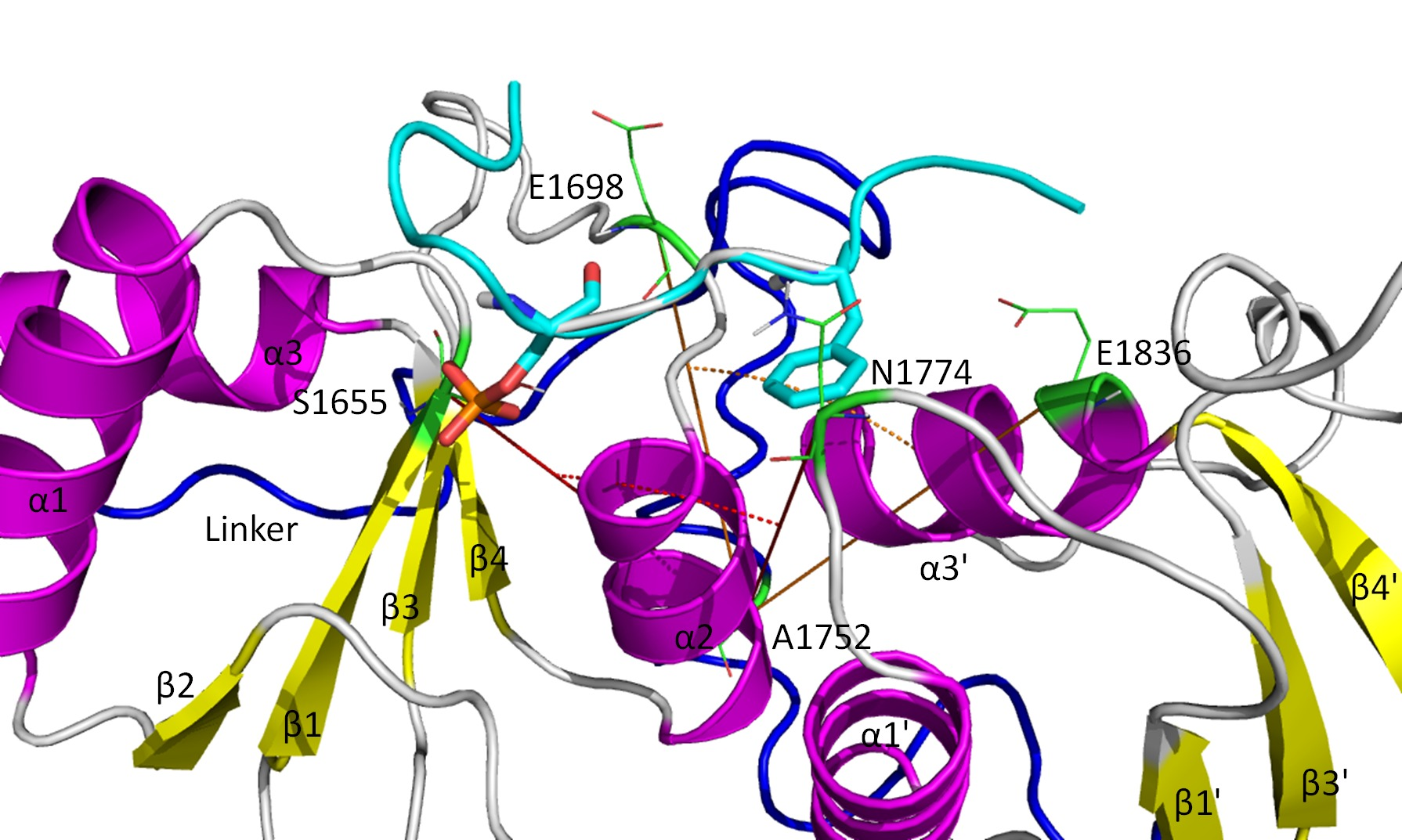

Supplement: S6 Fig — (TIF) [file pcbi.1005057.s009.tif]

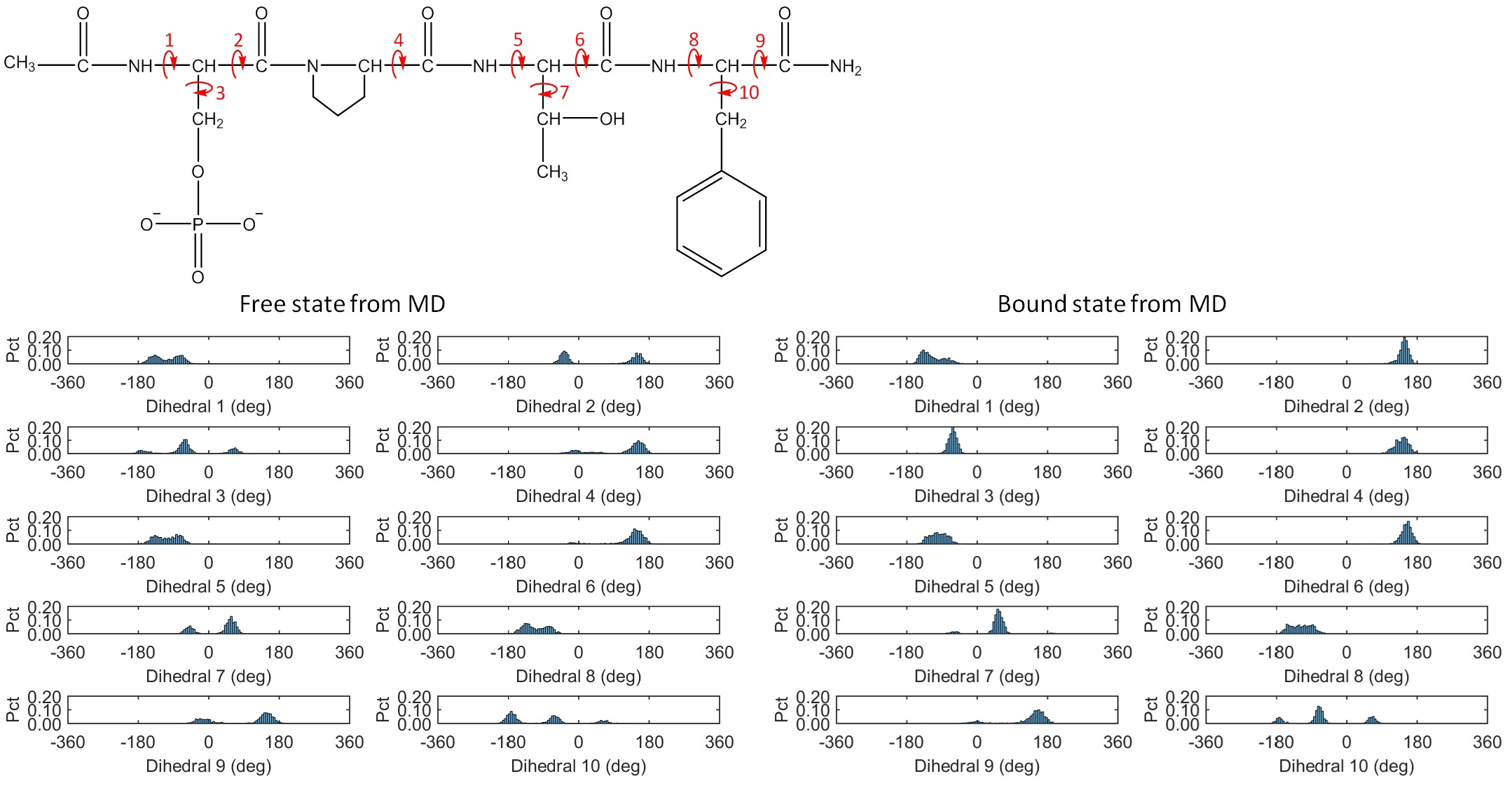

Supplement: S9 Fig — (TIF) [file pcbi.1005057.s012.tif]

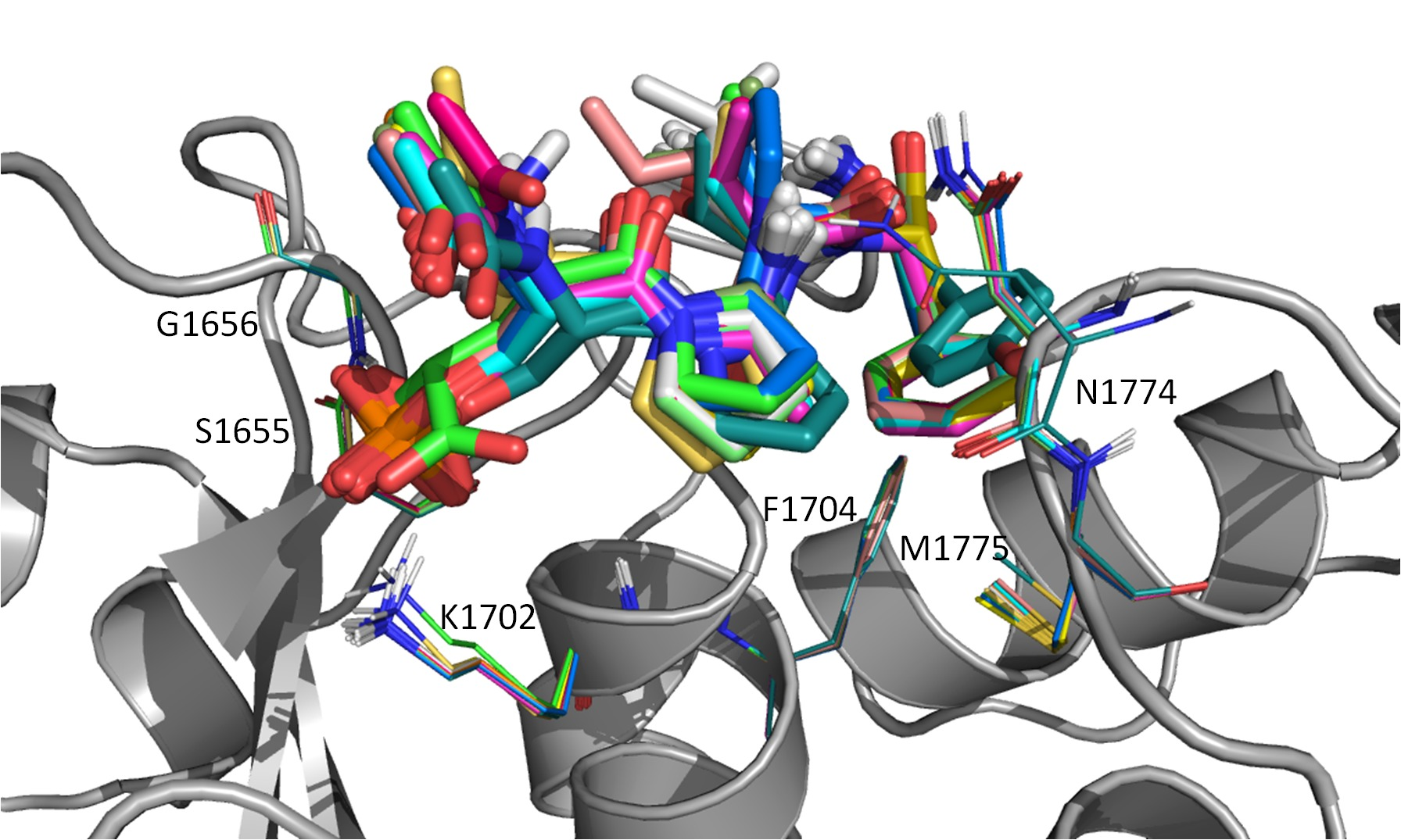

Supplement: S10 Fig — The phosphate groups and phosphate mimic are anchored by S1655, G1656, K1702 and phenylalanine/tyrosine surrounded by F1704, N1774, M1775. Ligands are shown in licorice representation, residues of BRCT are shown in line representation. (TIF) [file pcbi.1005057.s013.tif]

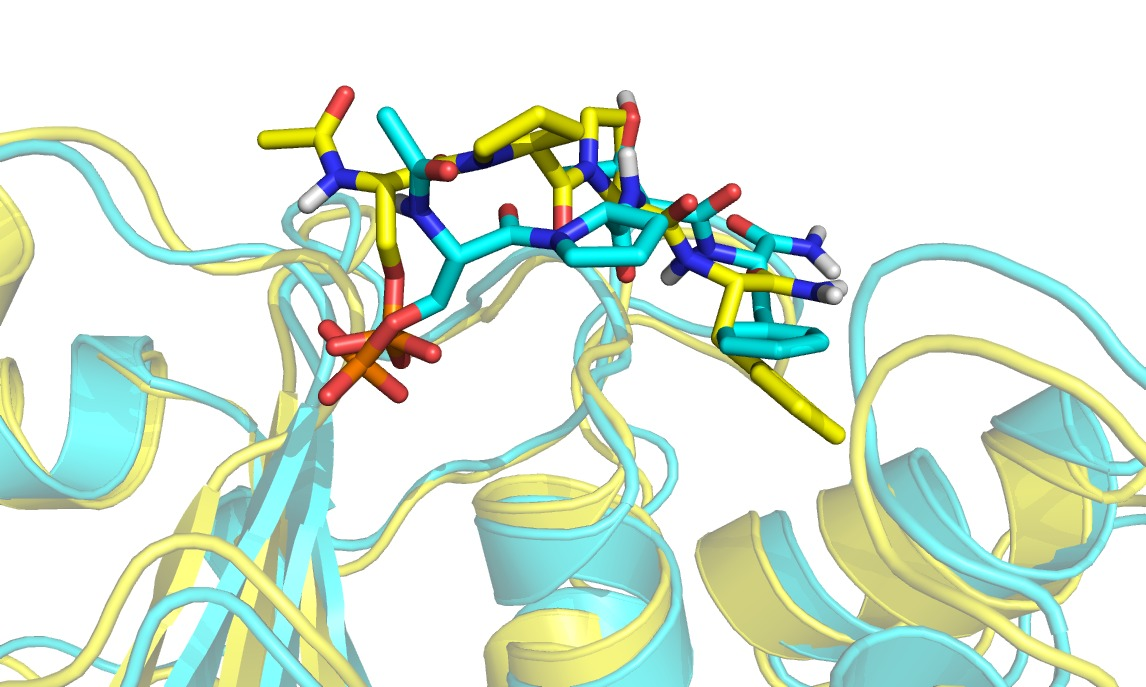

Supplement: S11 Fig — The bound conformation of P4 is represents the standard bound conformation of most phosphopeptides. Changes in the bound conformation start showing up right after the mutation at the P+2 position from threonine or valine to proline. In P13, in order to align phosphate group and benzene ring of phenylalanine, the whole backbone frame of the ligand has to move towards solvent to moderate the restrain from two rigid proline residues in the middle, which causes the improper fit of P13 in the cavity, resulting in increased enthalpy change and high entropy cost. (TIF) [file pcbi.1005057.s014.tif]

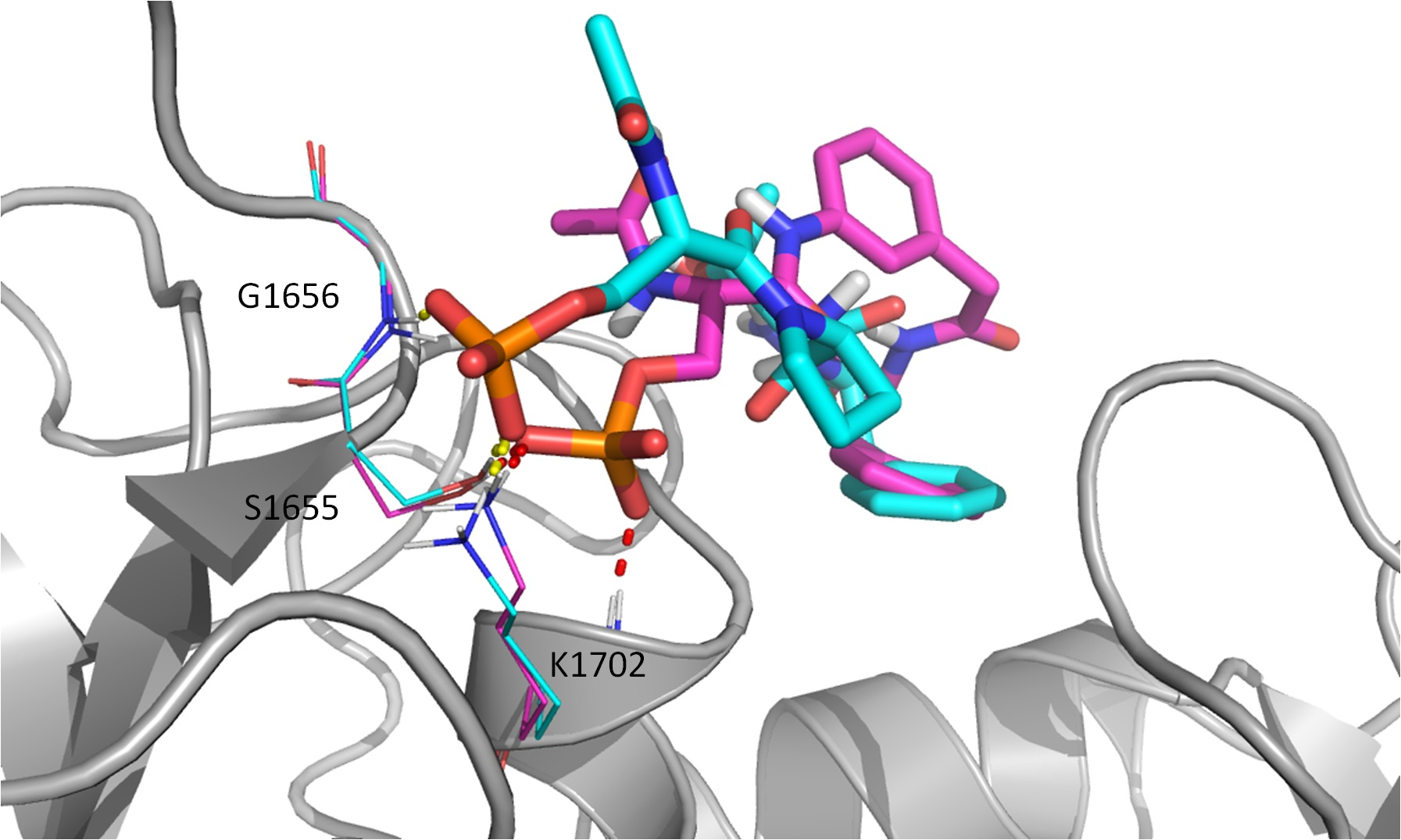

Supplement: S12 Fig — Ligands are shown in licorice representation, residues of BRCT are shown in line representation. Hydrogen bonds are drawn in dash lines. (TIF) [file pcbi.1005057.s015.tif]

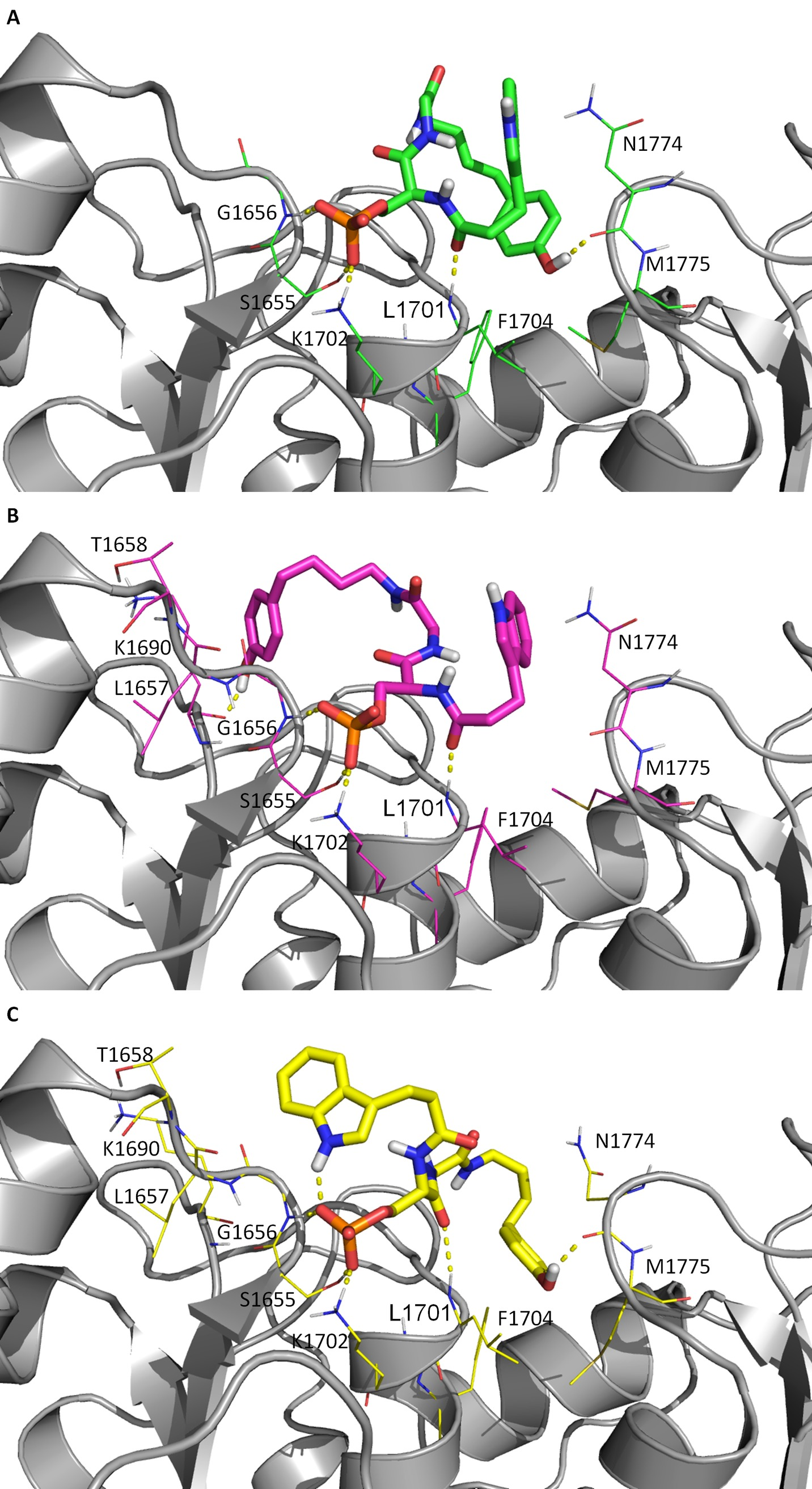

Supplement: S13 Fig — Residues of BRCT are shown in line mode and the ligand is shown in licorice mode, hydrogen bonds are drawn in dash lines (free energies of A, B and C bound conformations are -1476.72, -1476.28 and -1465.36 kcal/mol, respectively). (TIF) [file pcbi.1005057.s016.tif]
