# Supplementary material for: Characterization of Promiscuous Binding of Phosphor Ligands to Breast-Cancer-Gene 1 (BRCA1) C-Terminal (BRCT): Molecular Dynamics, Free Energy, Entropy and Inhibitor Design
Source: PLoS Comput Biol. 2016 Aug 25;12(8):e1005057. doi: 10.1371/journal.pcbi.1005057 (PMC4999267; doi:10.1371/journal.pcbi.1005057)

**S7 Fig**. Ligand P1-P3, P5-P14, N1 and D1 structures with selected rotatable bonds and dihedral angle distributions from MD and M2.

P1

free state from MD bound state from MD
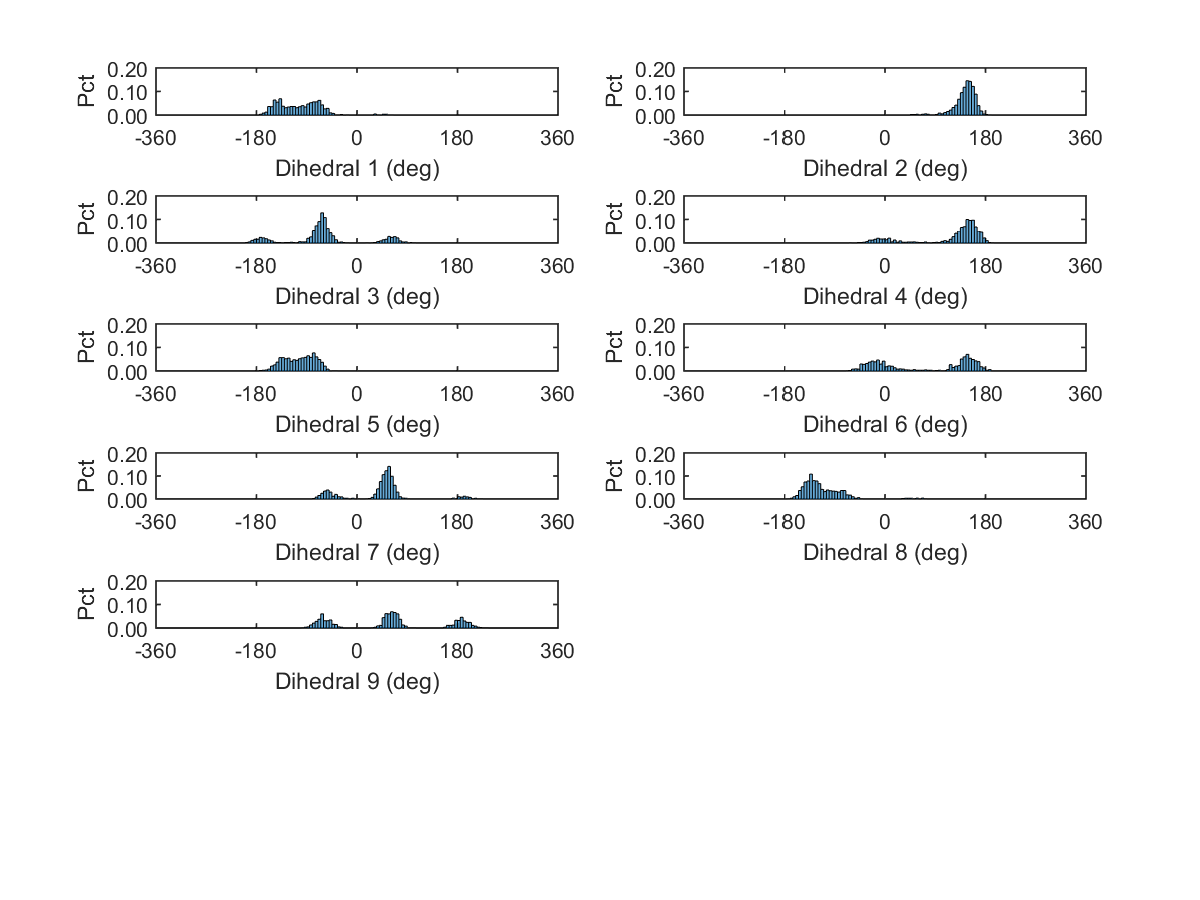

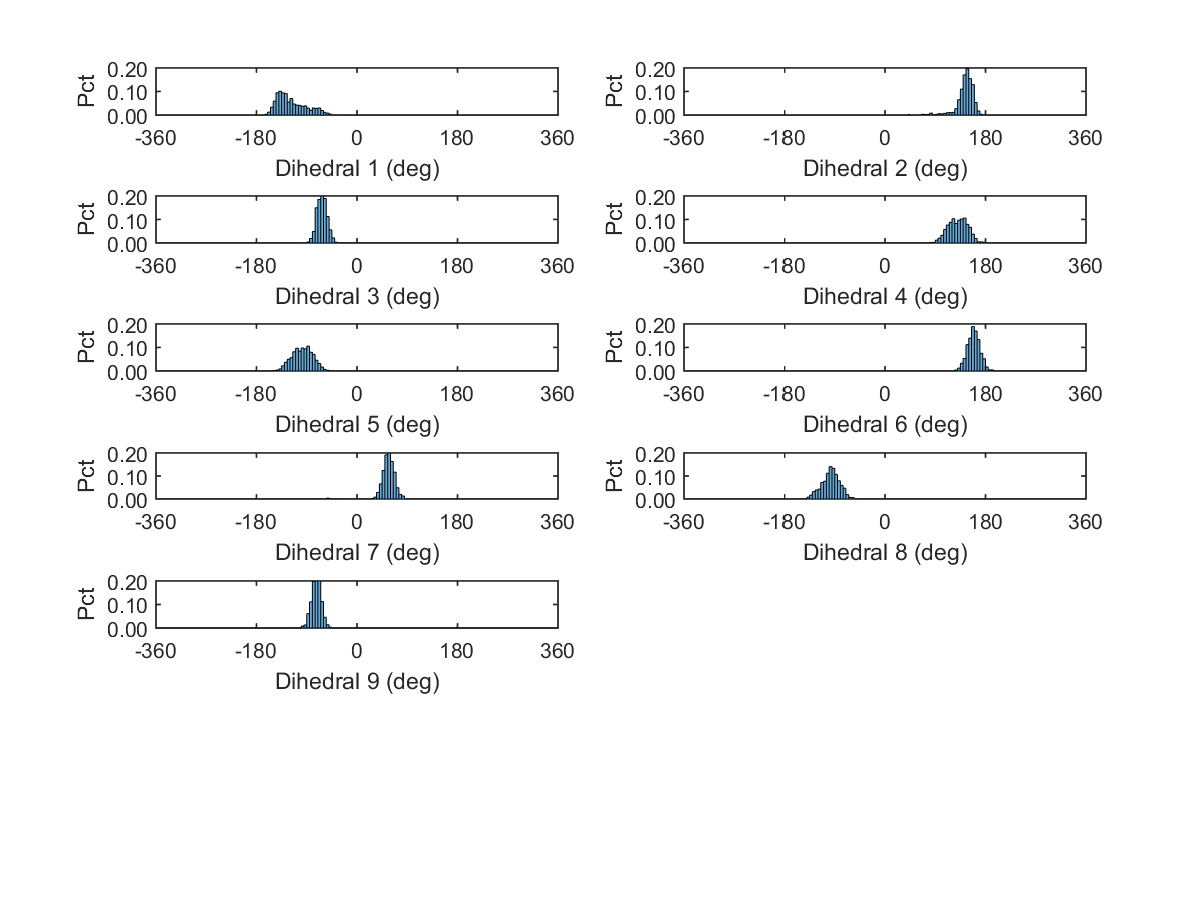


free state from M2 bound state from M2


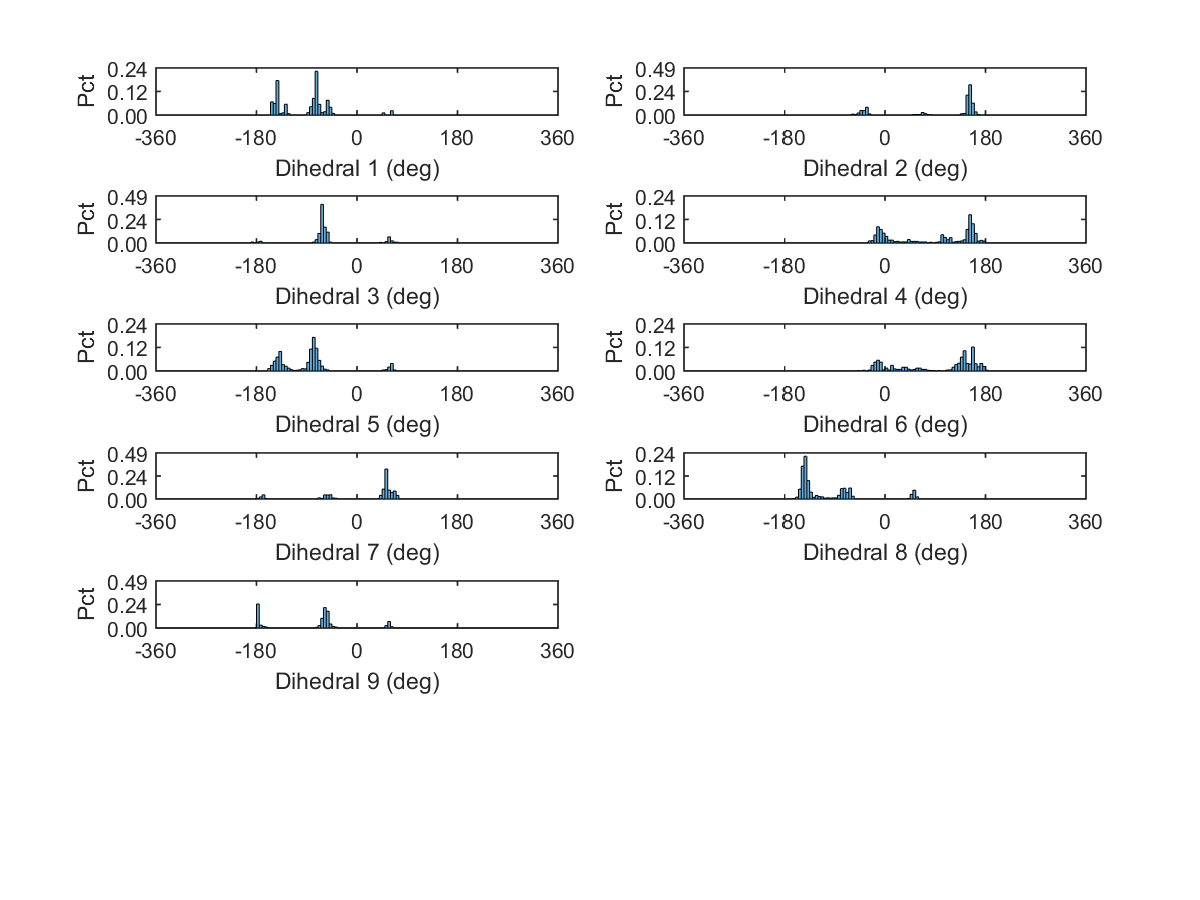

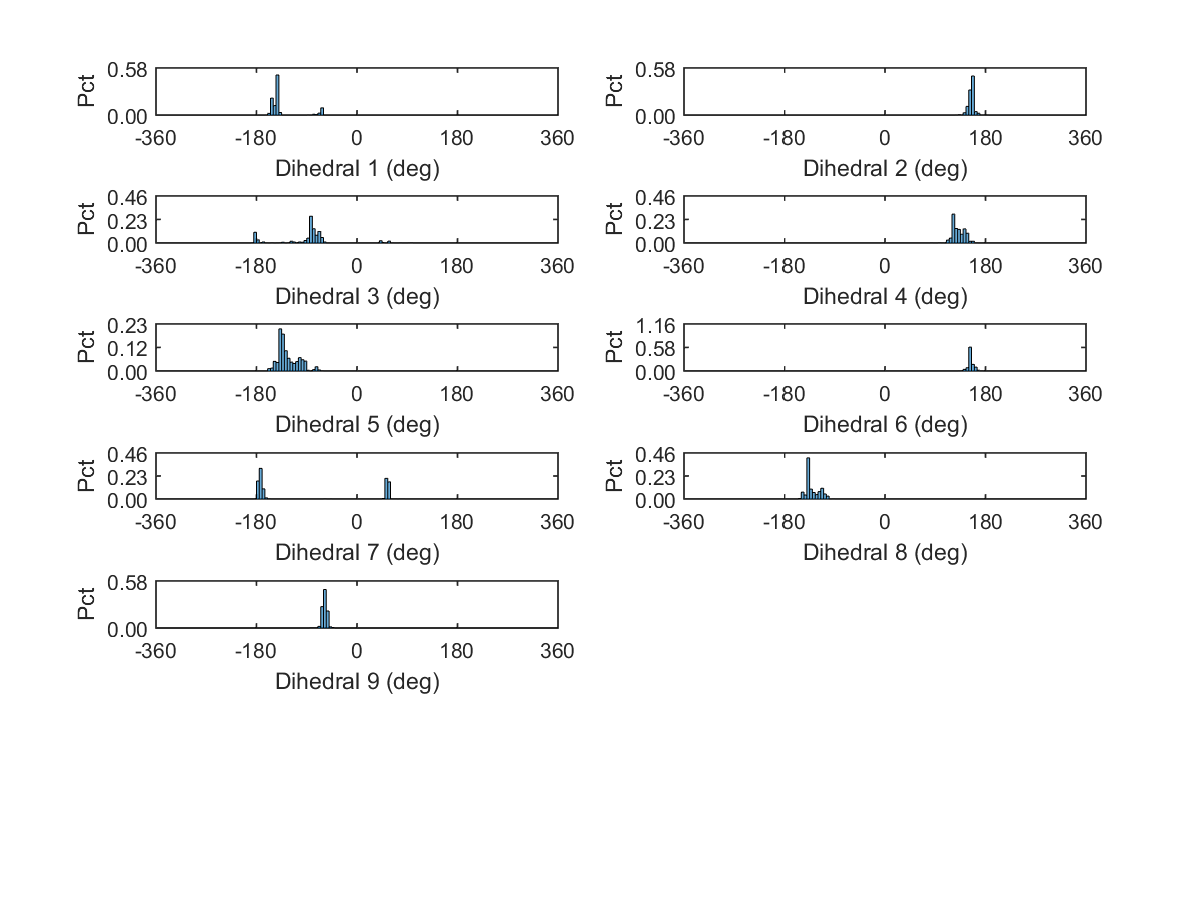


P2

free state from MD bound state from MD


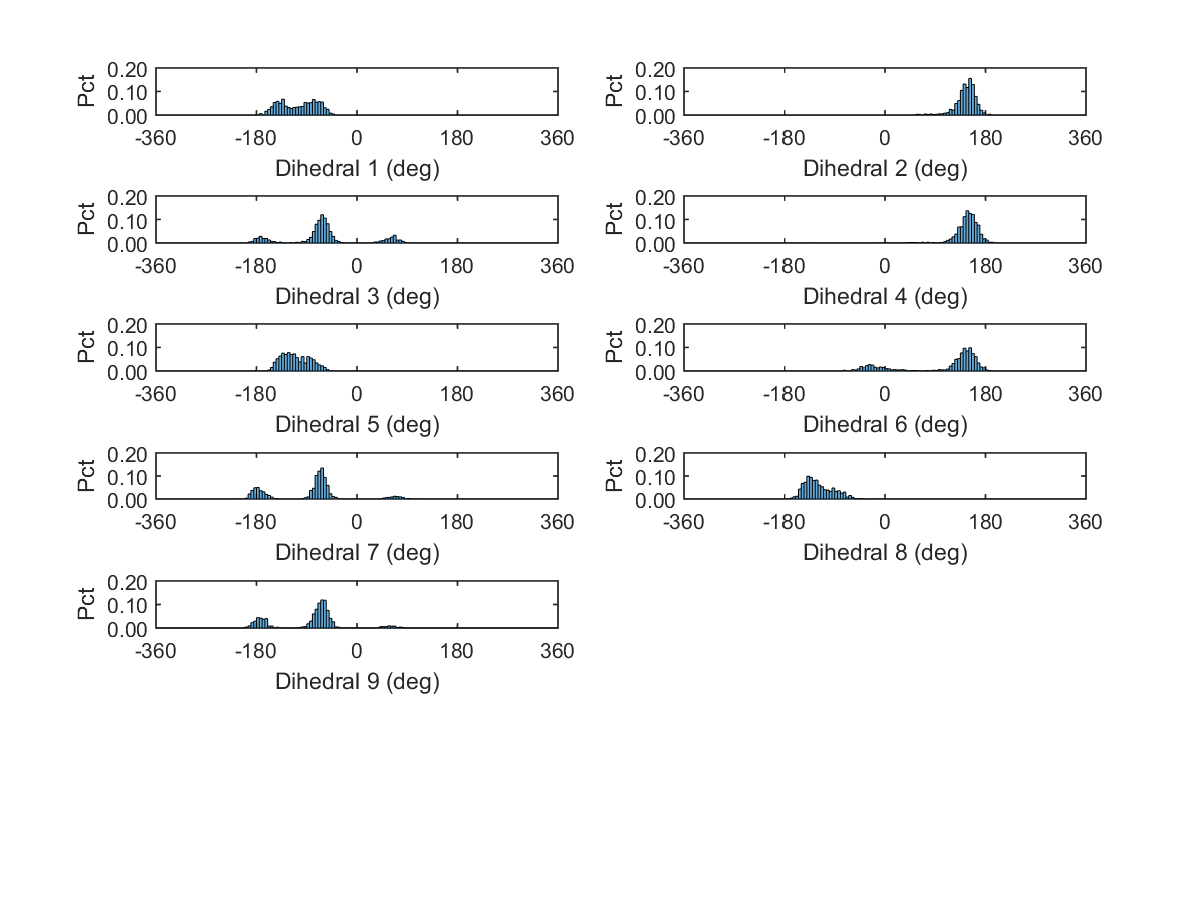

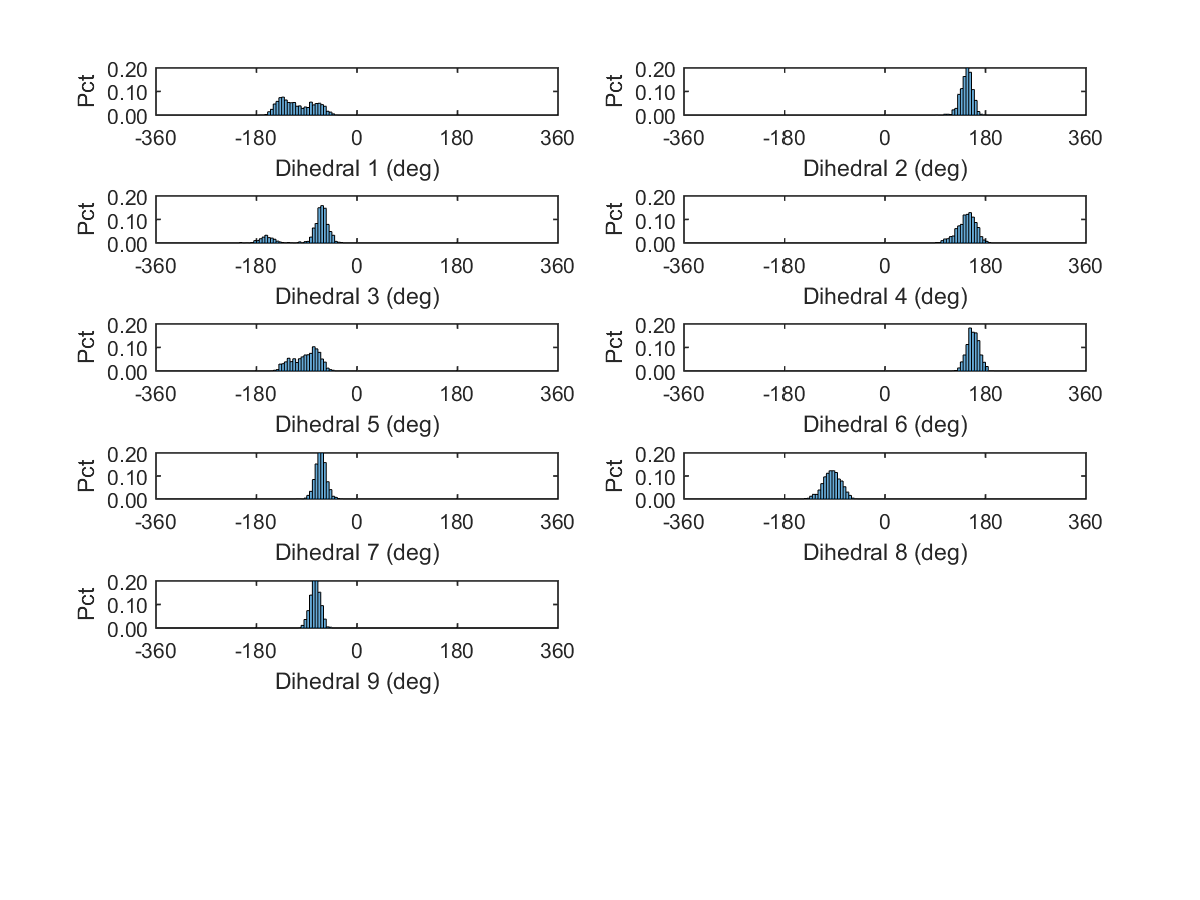


free state from M2 bound state from M2


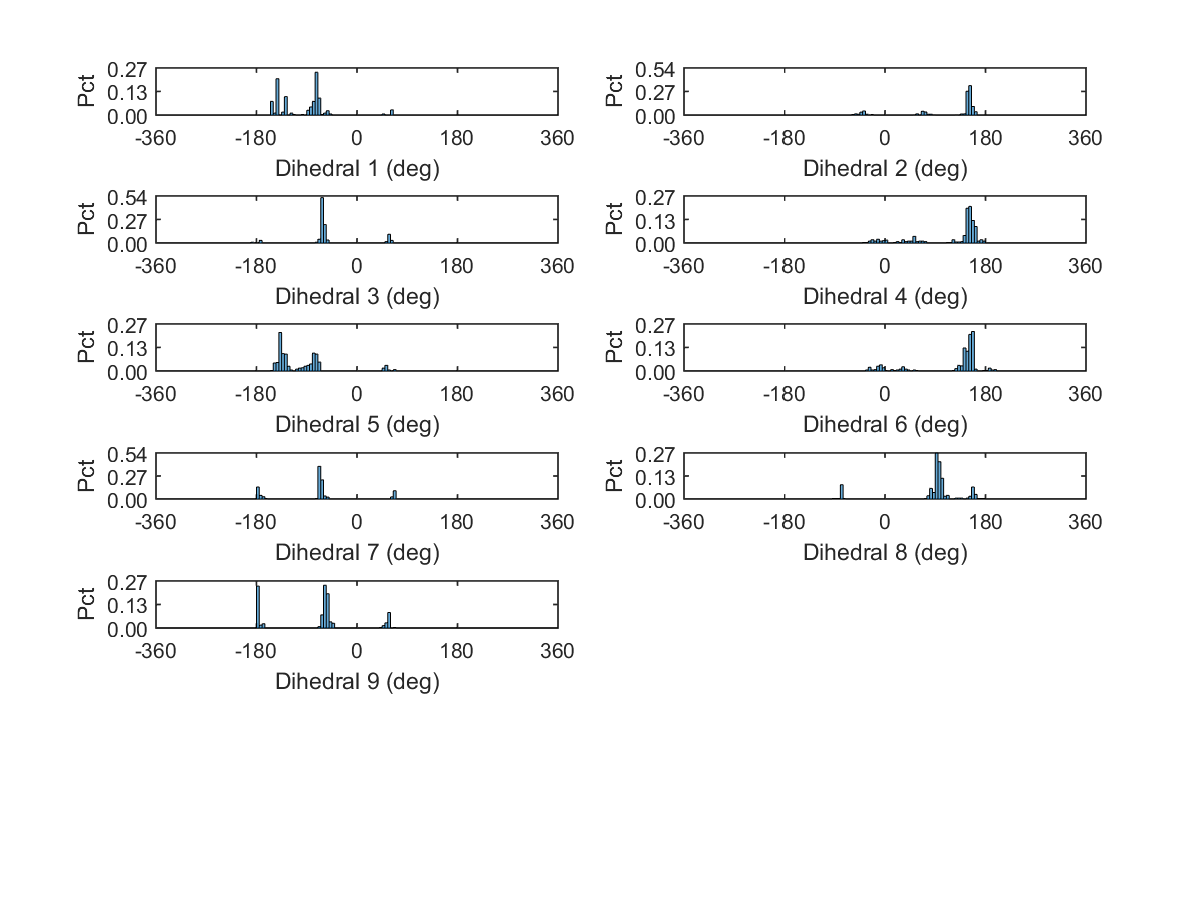

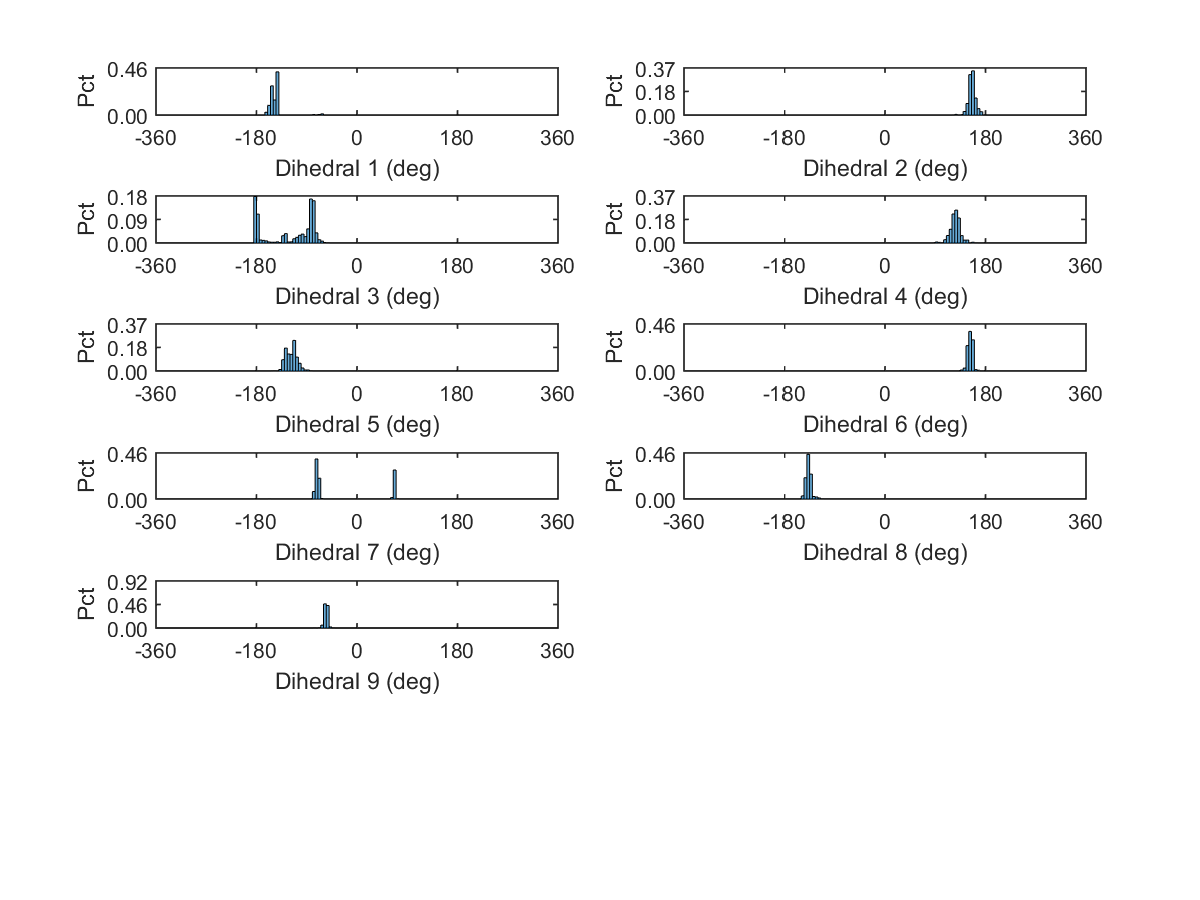


P3

free state from MD bound state from MD


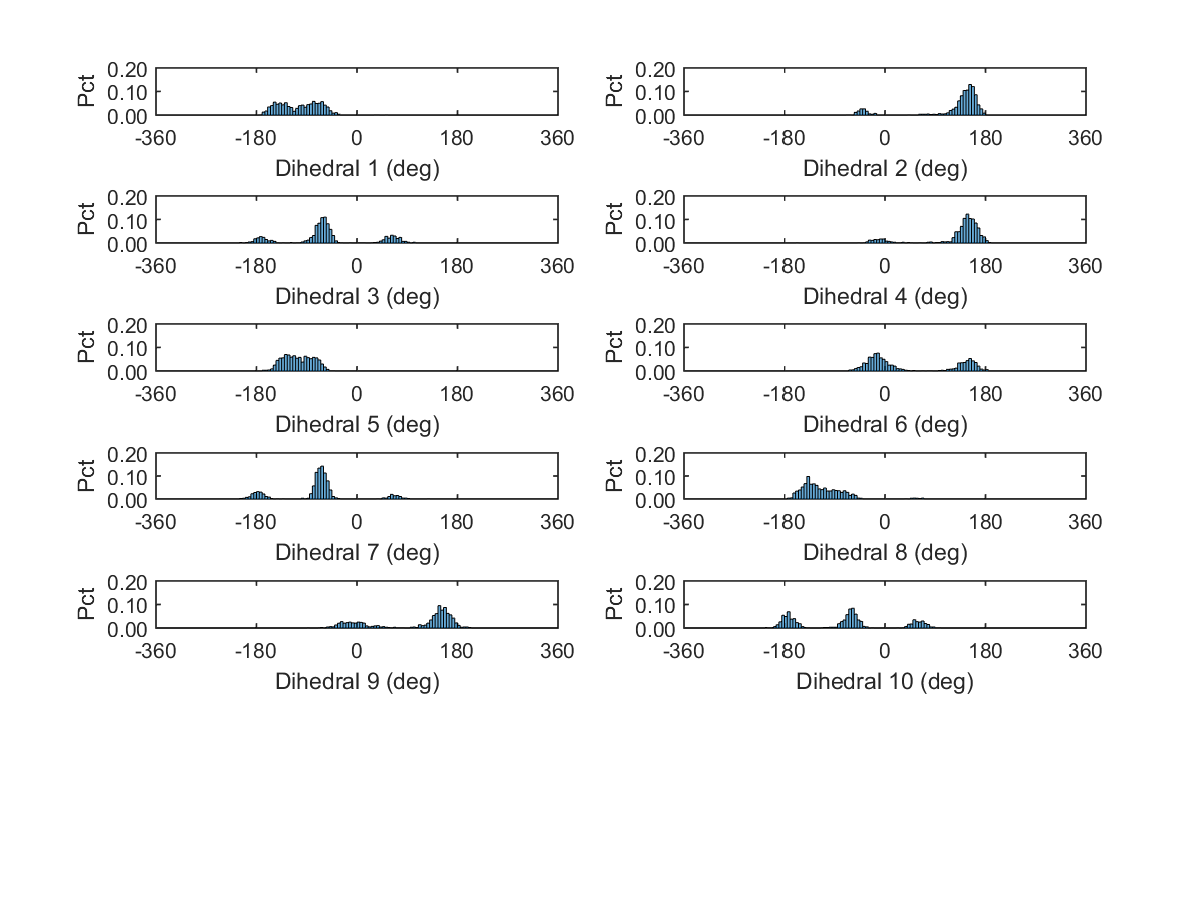

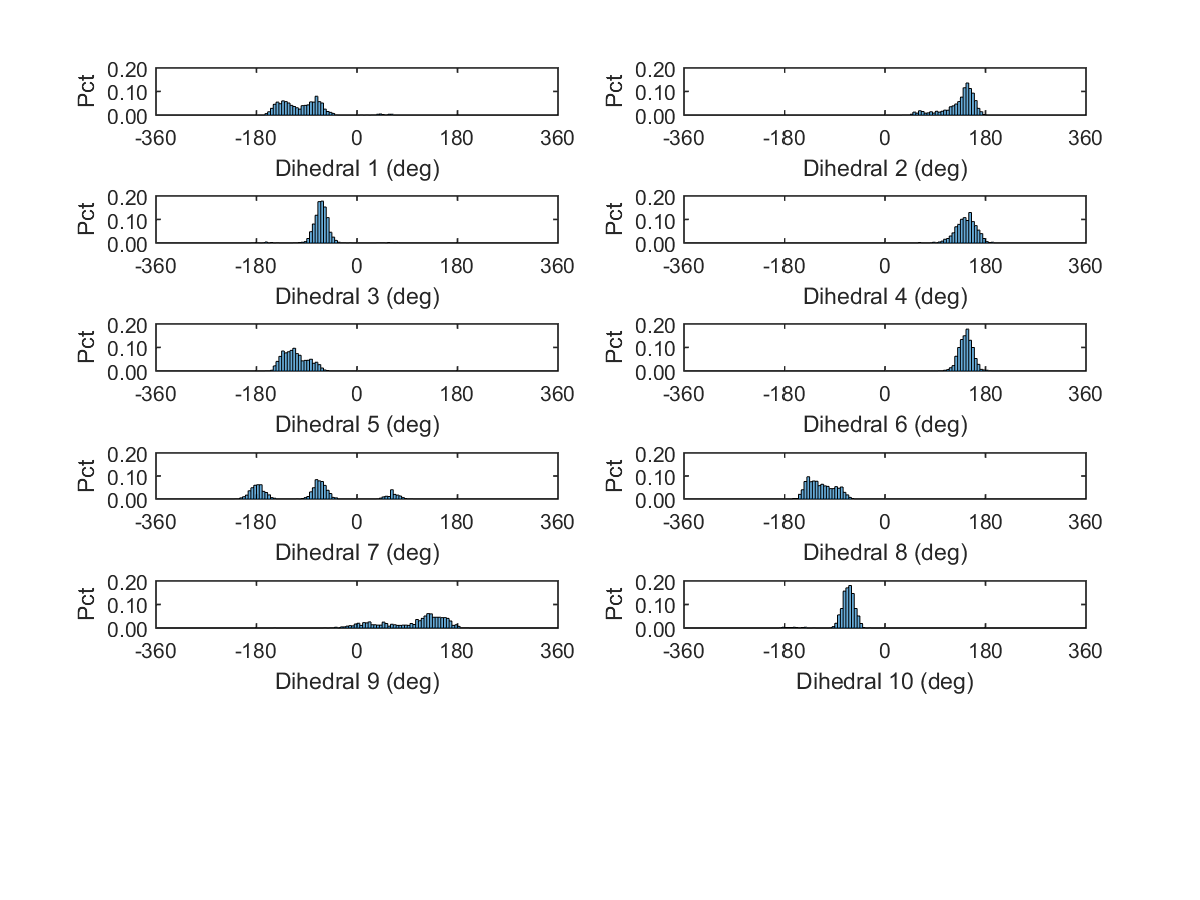


free state from M2 bound state from M2


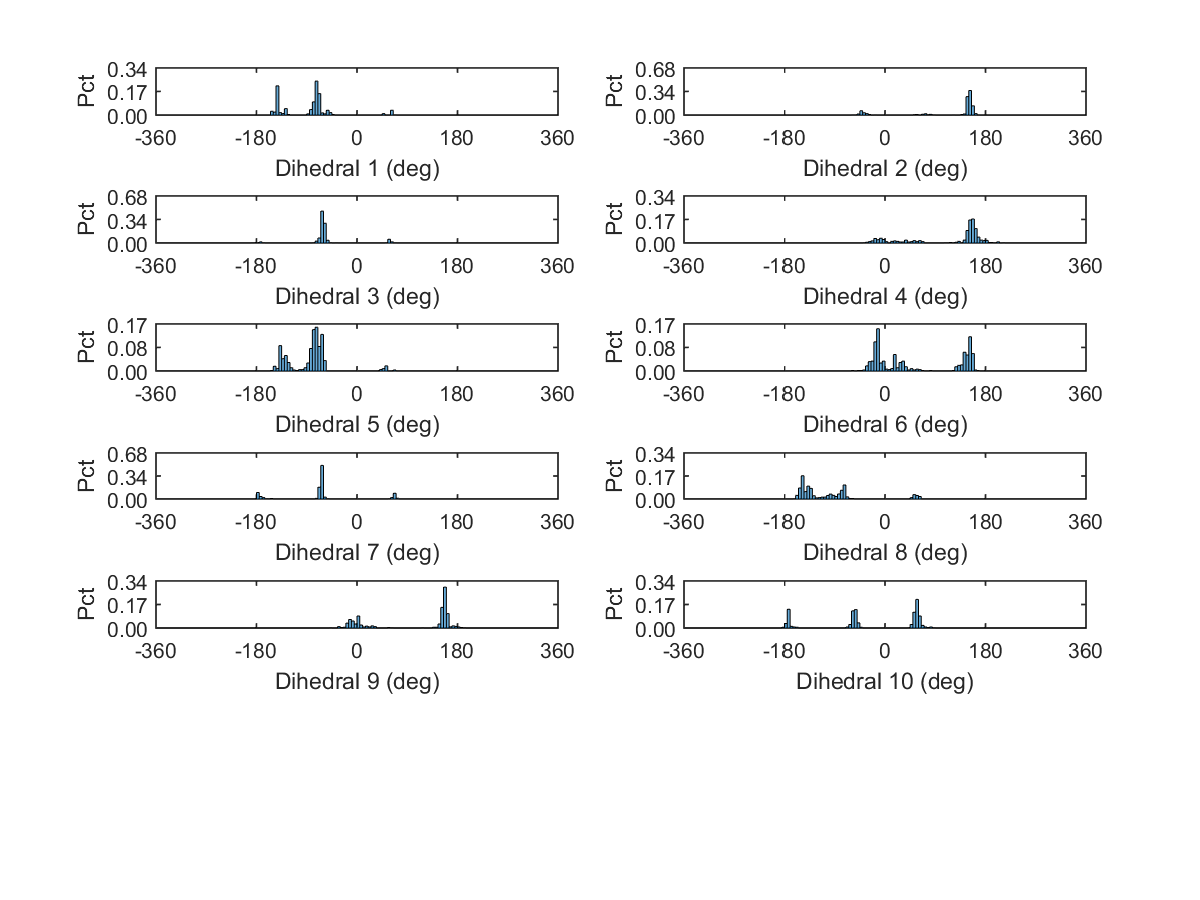

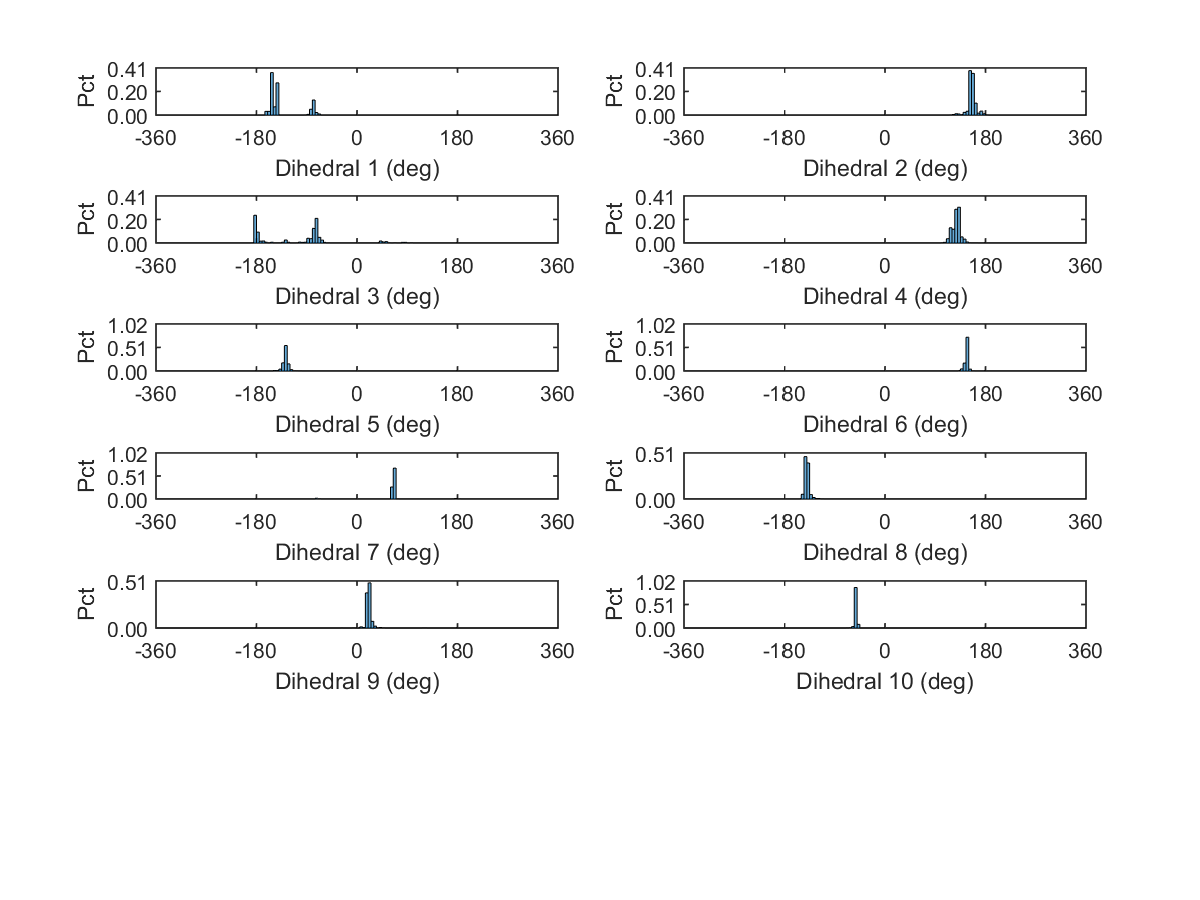


P5

free state from MD bound state from MD


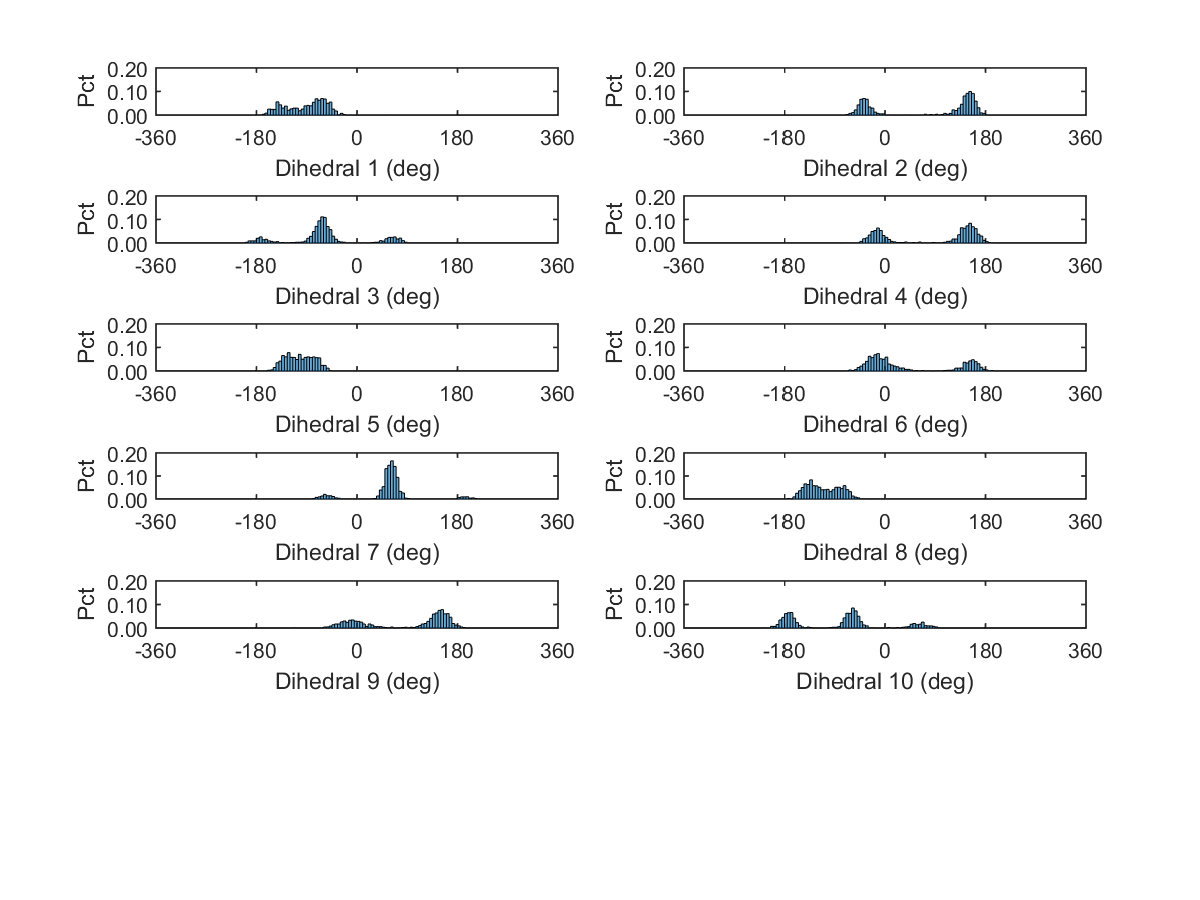

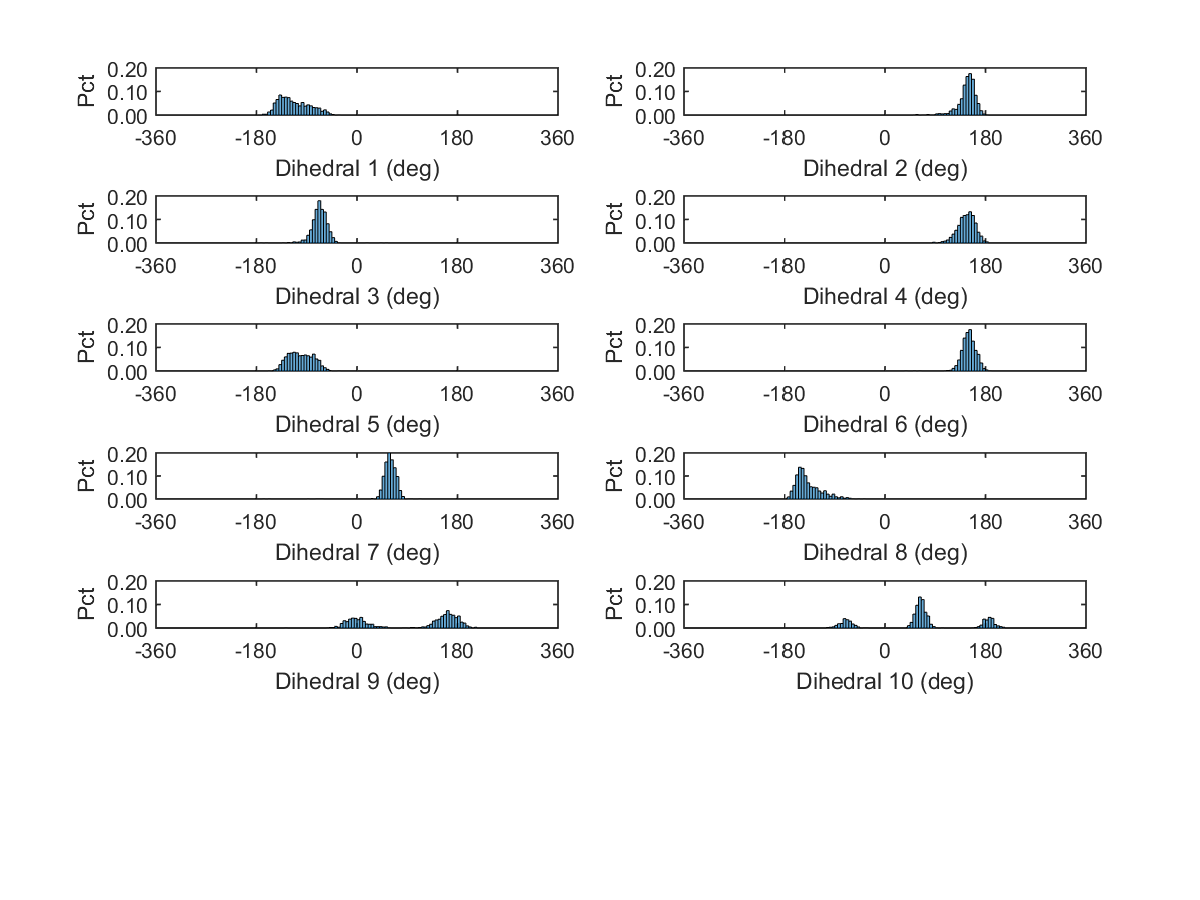


free state from M2 bound state from M2


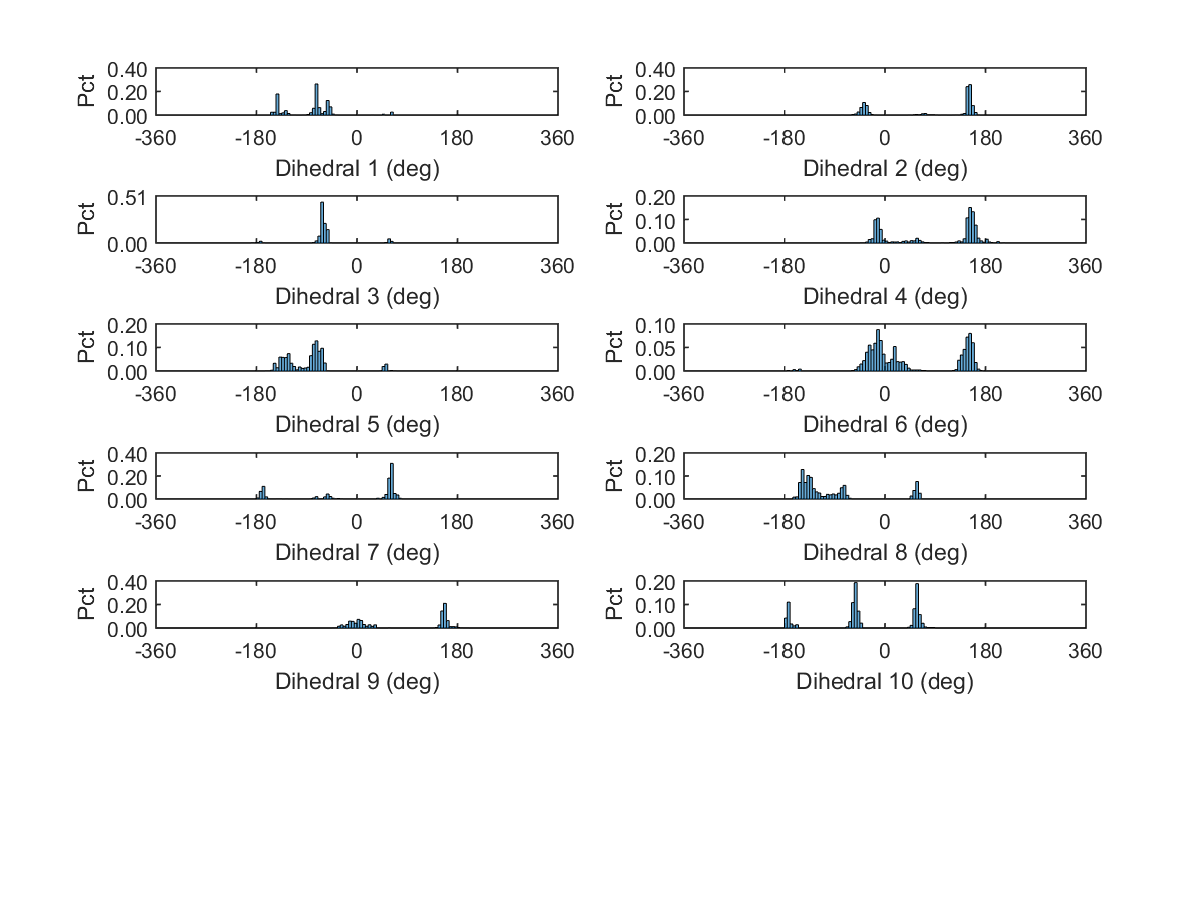

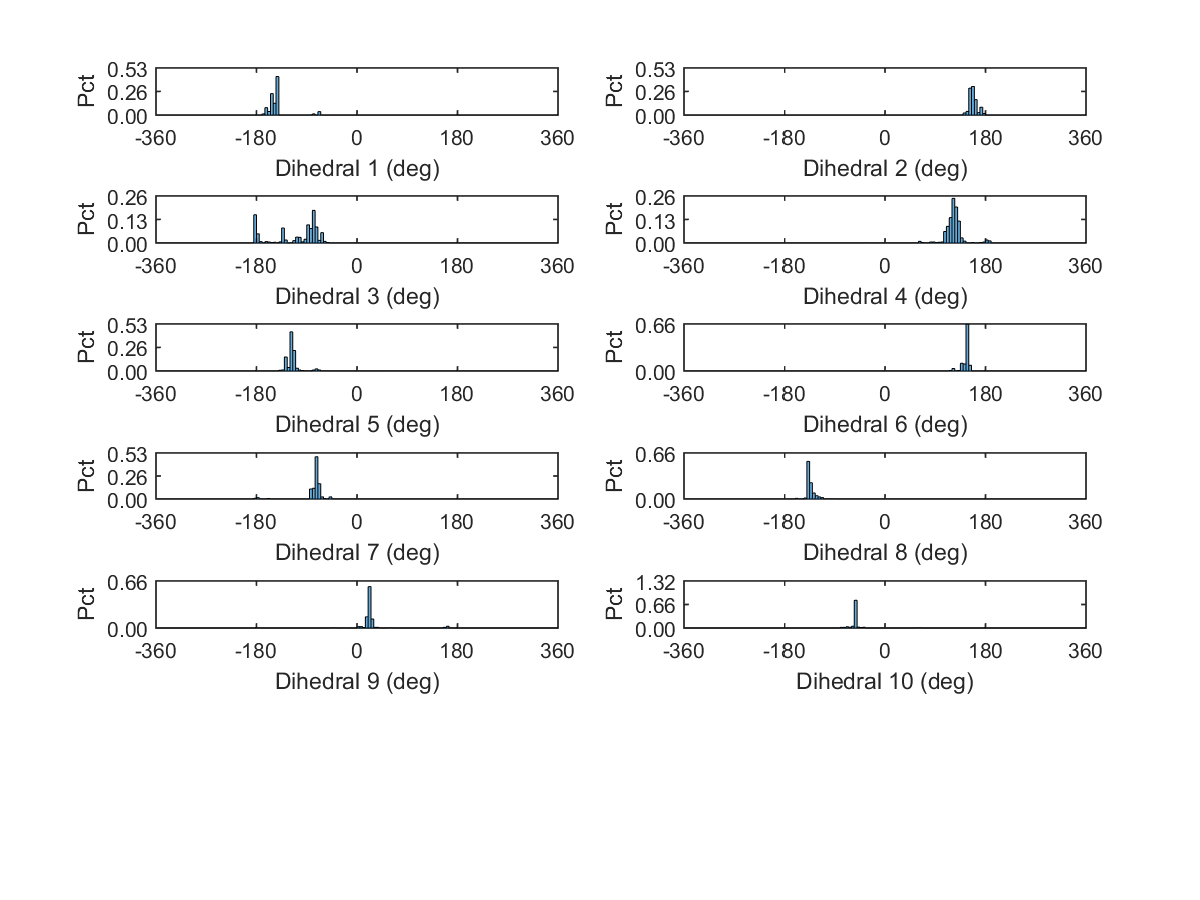


P6

free state from MD bound state from MD


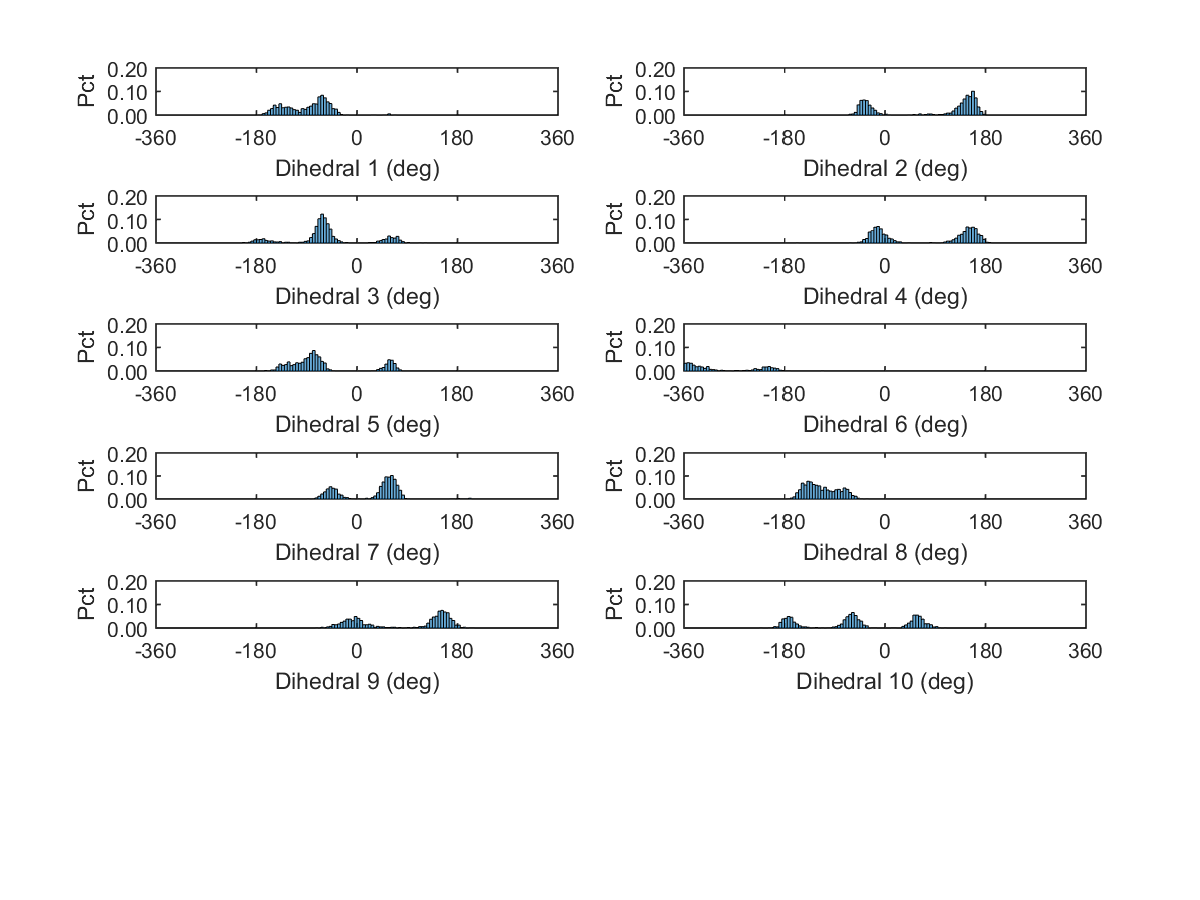

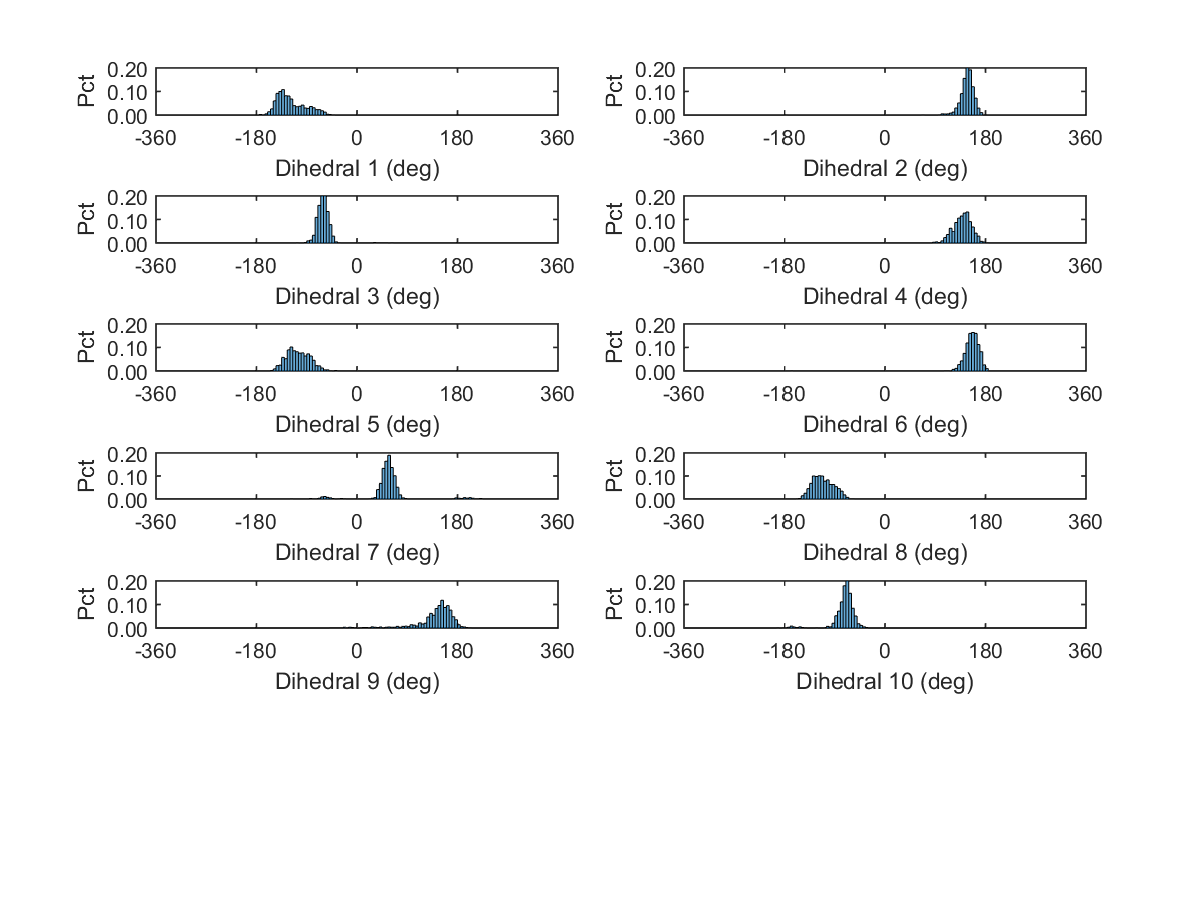


free state from M2 bound state from M2


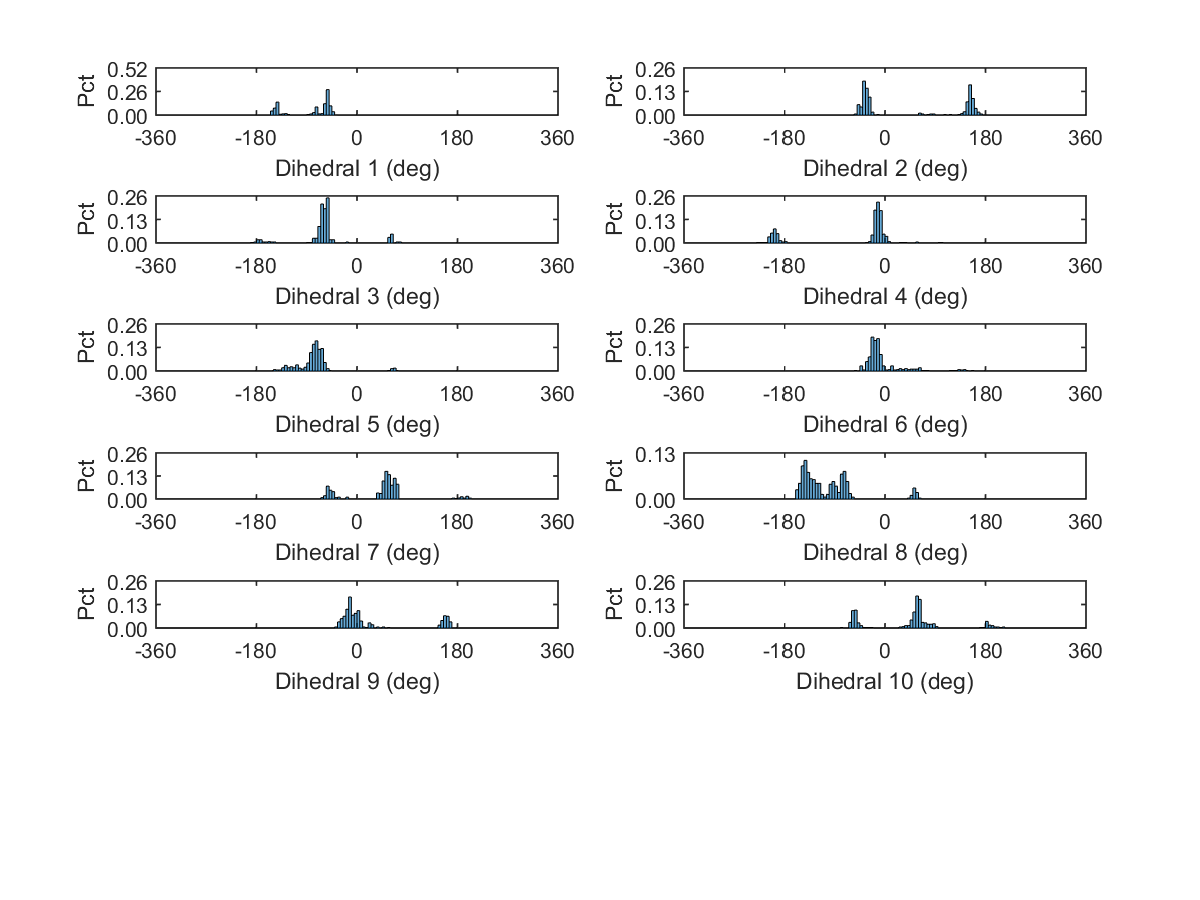

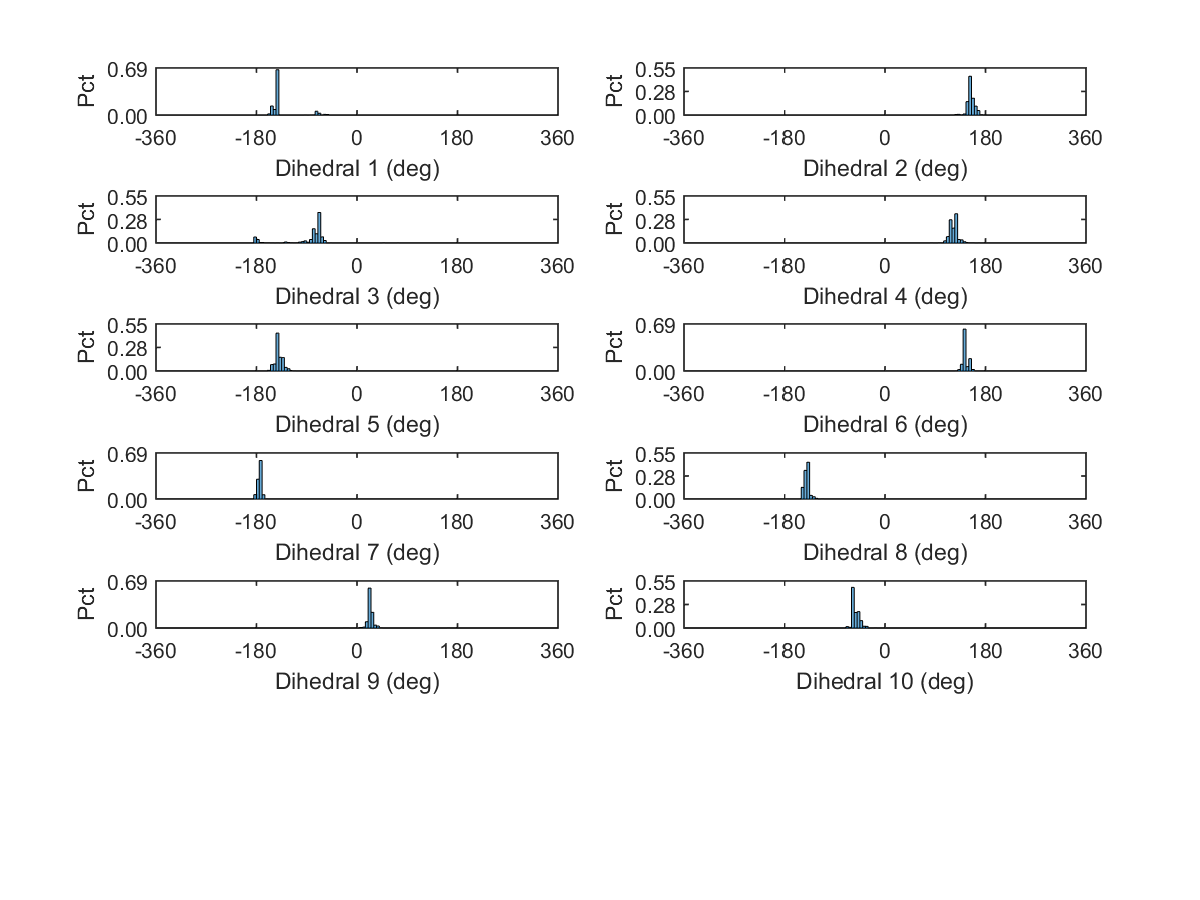


P7

free state from MD bound state from MD


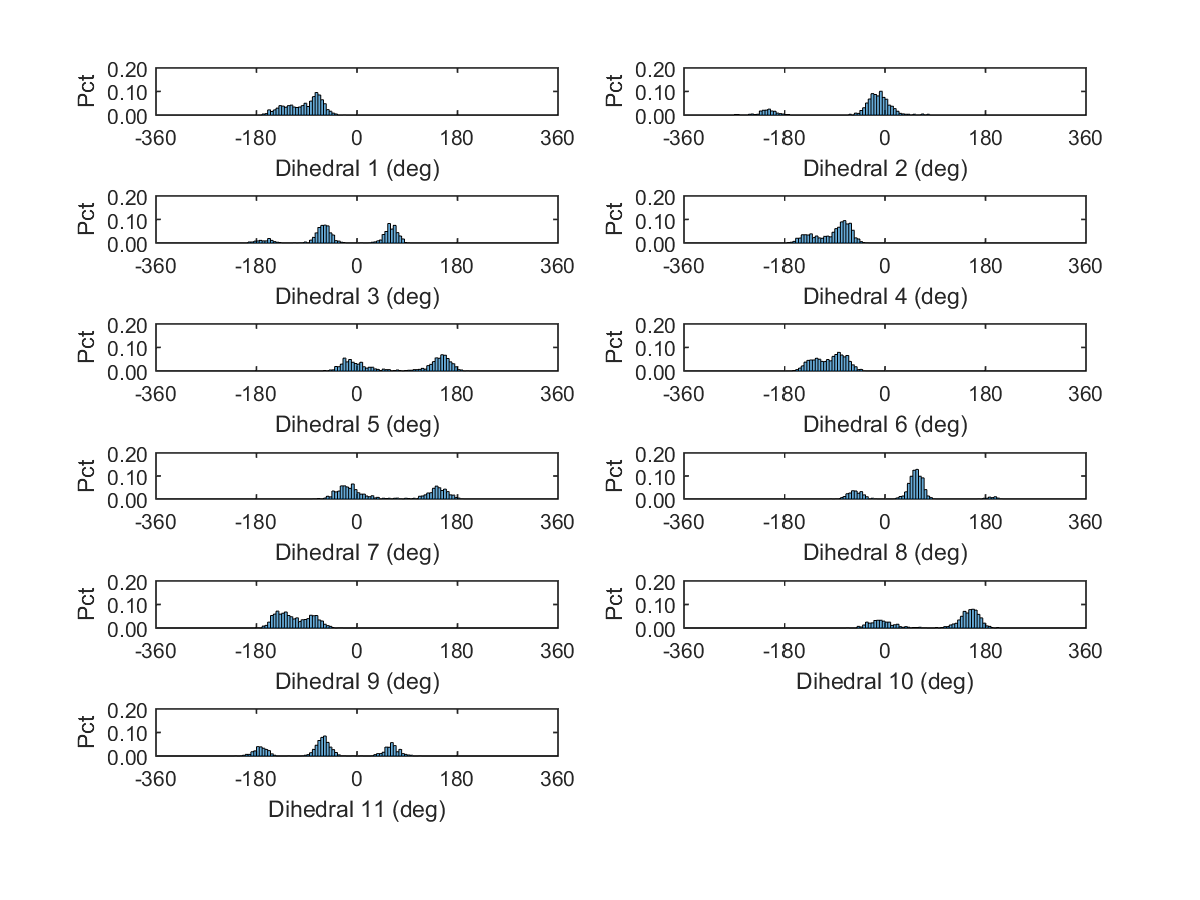

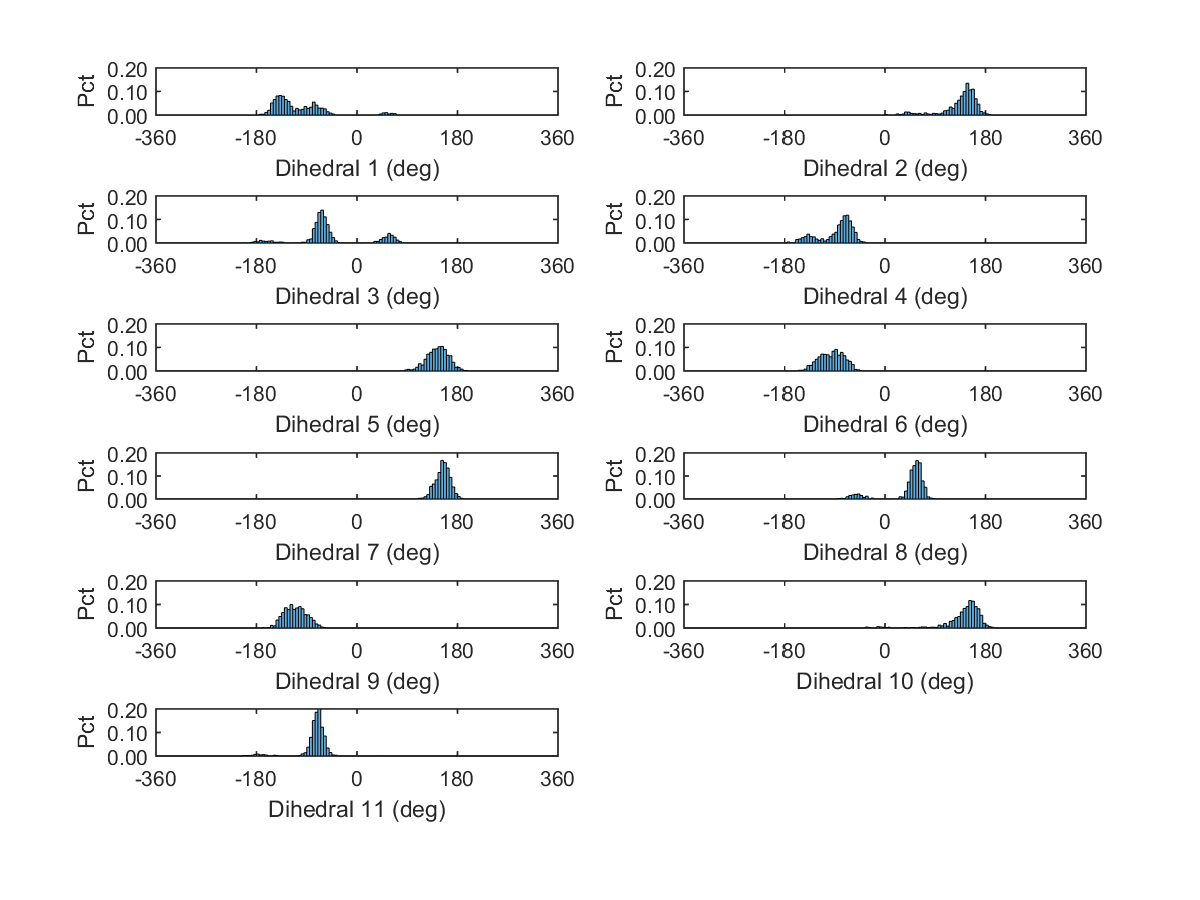


free state from M2 bound state from M2


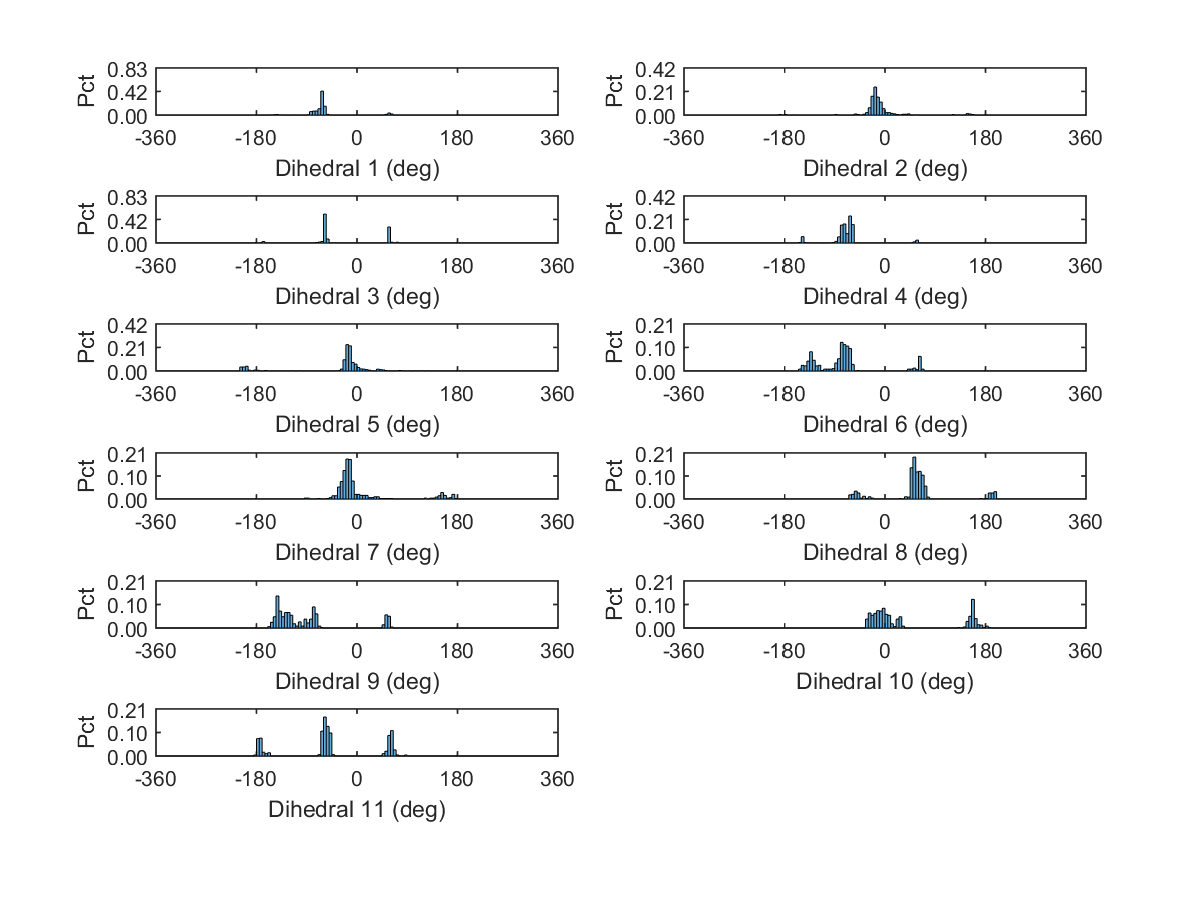

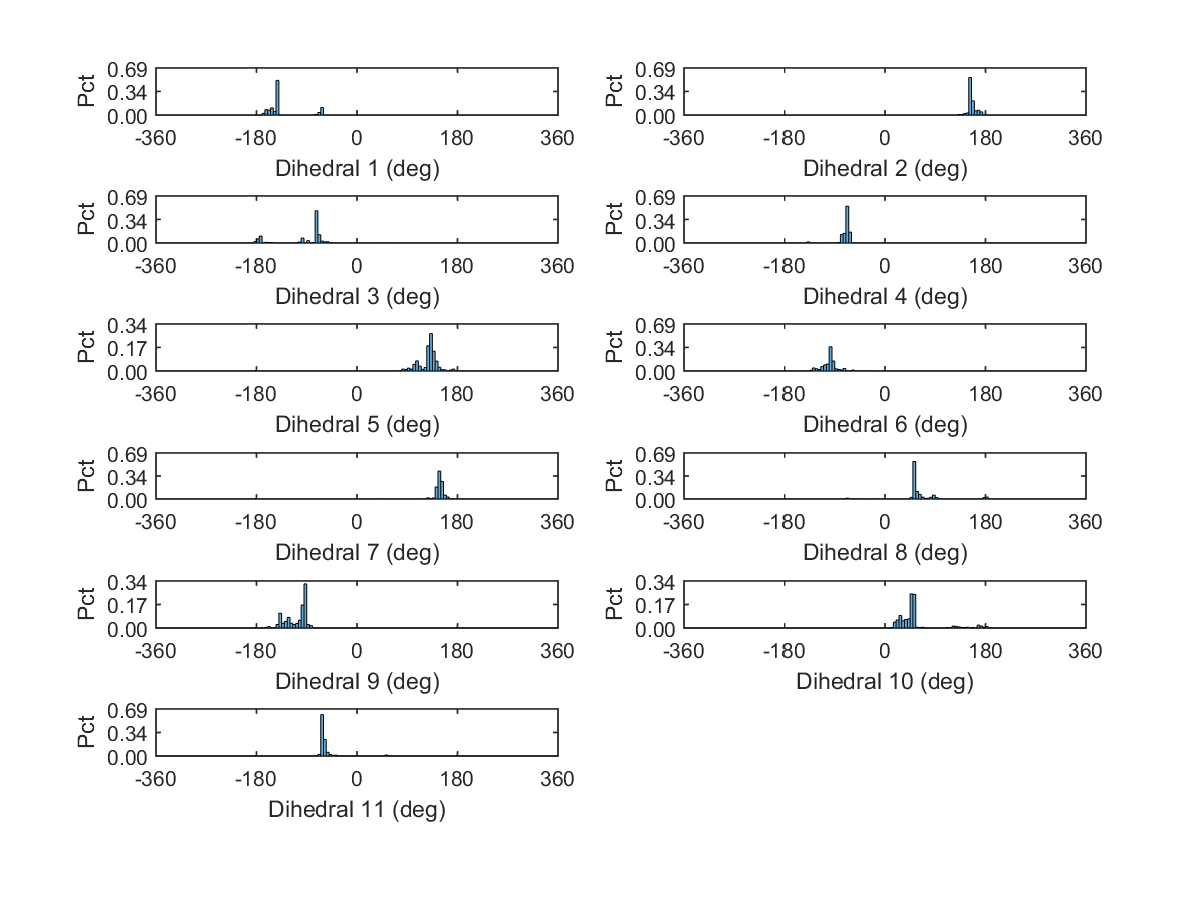


P8

free state from MD bound state from MD


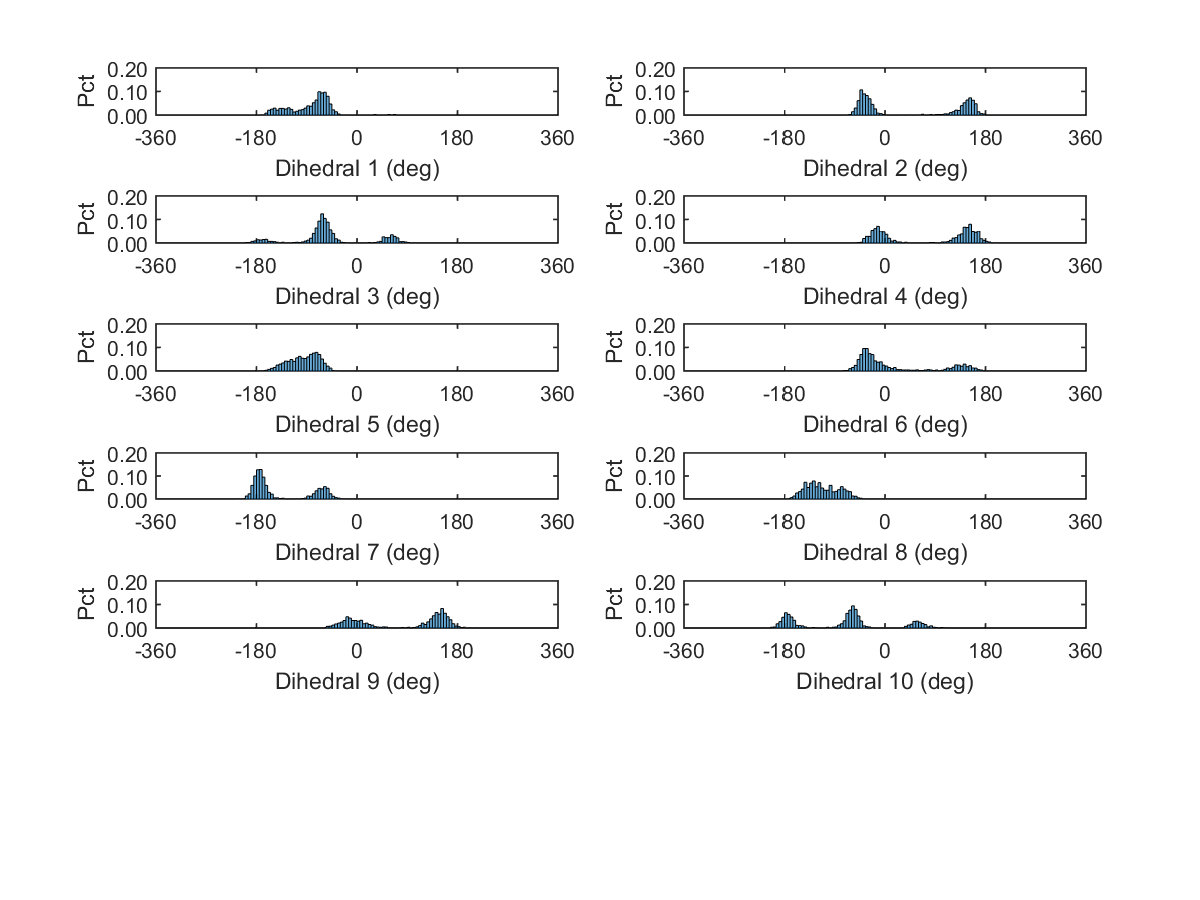

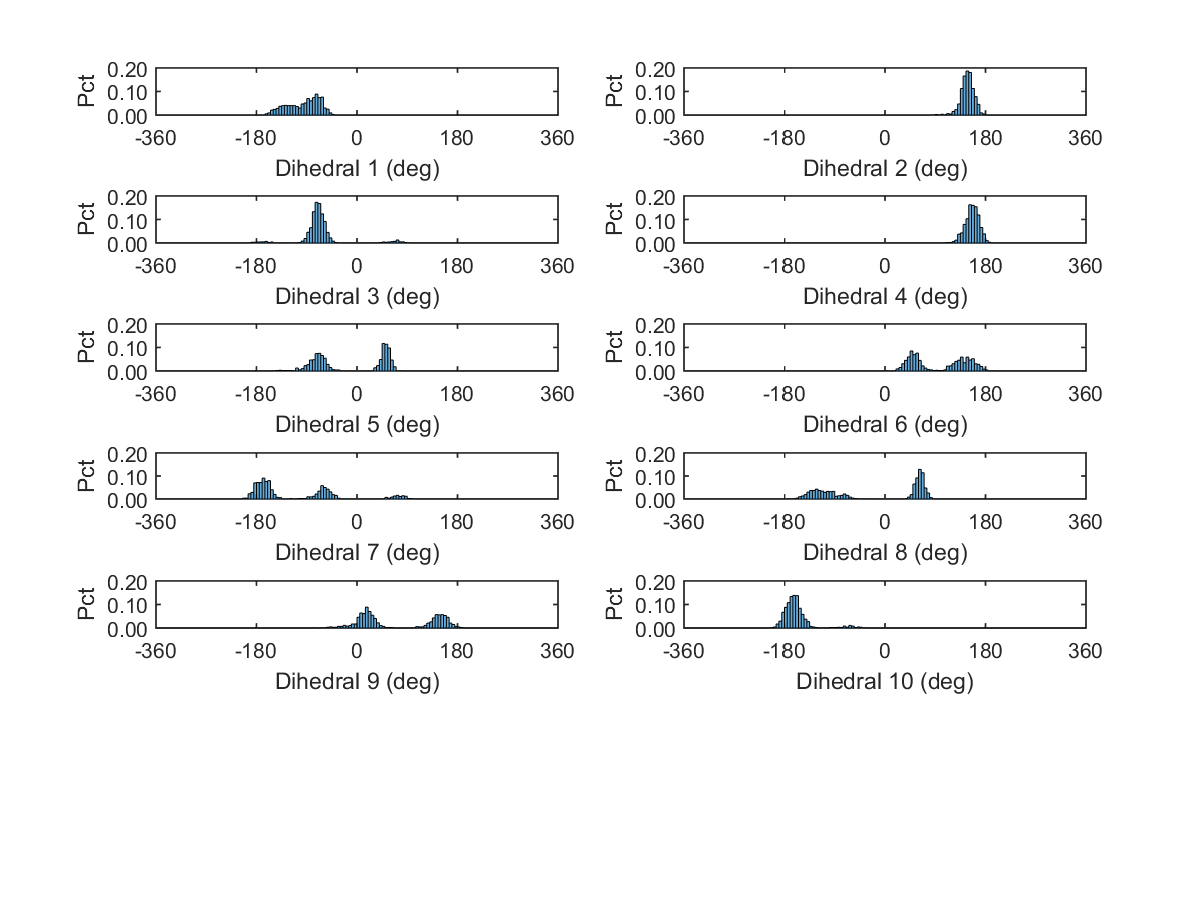


free state from M2 bound state from M2


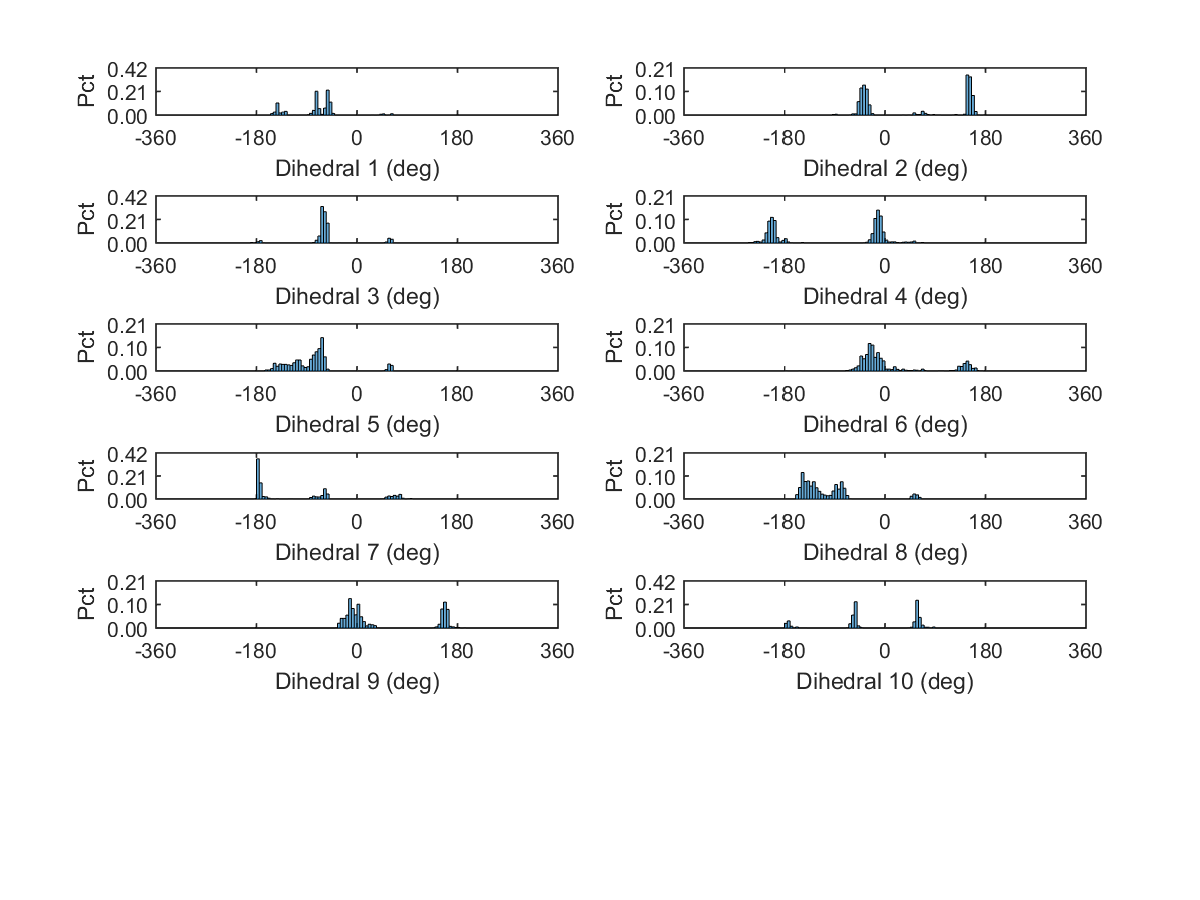

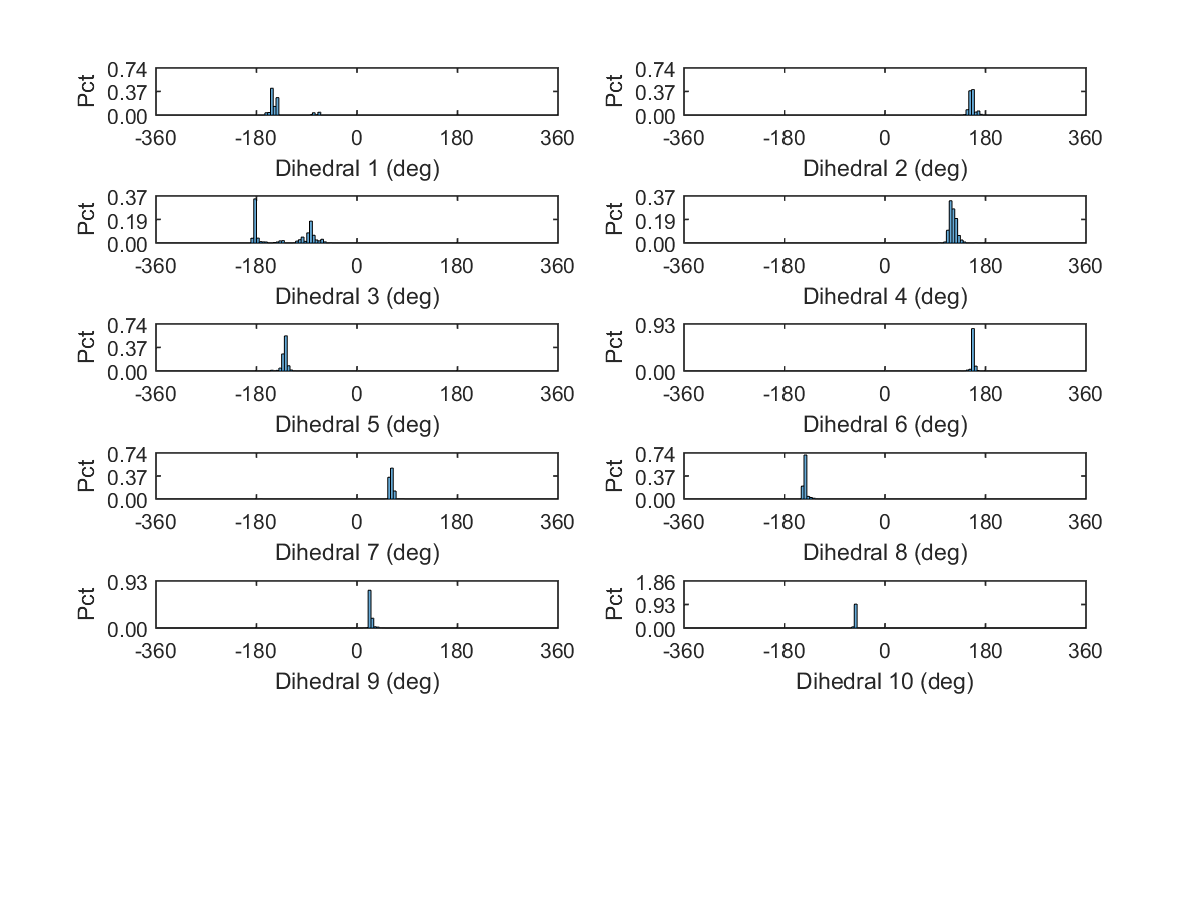


P9

free state from MD bound state from MD


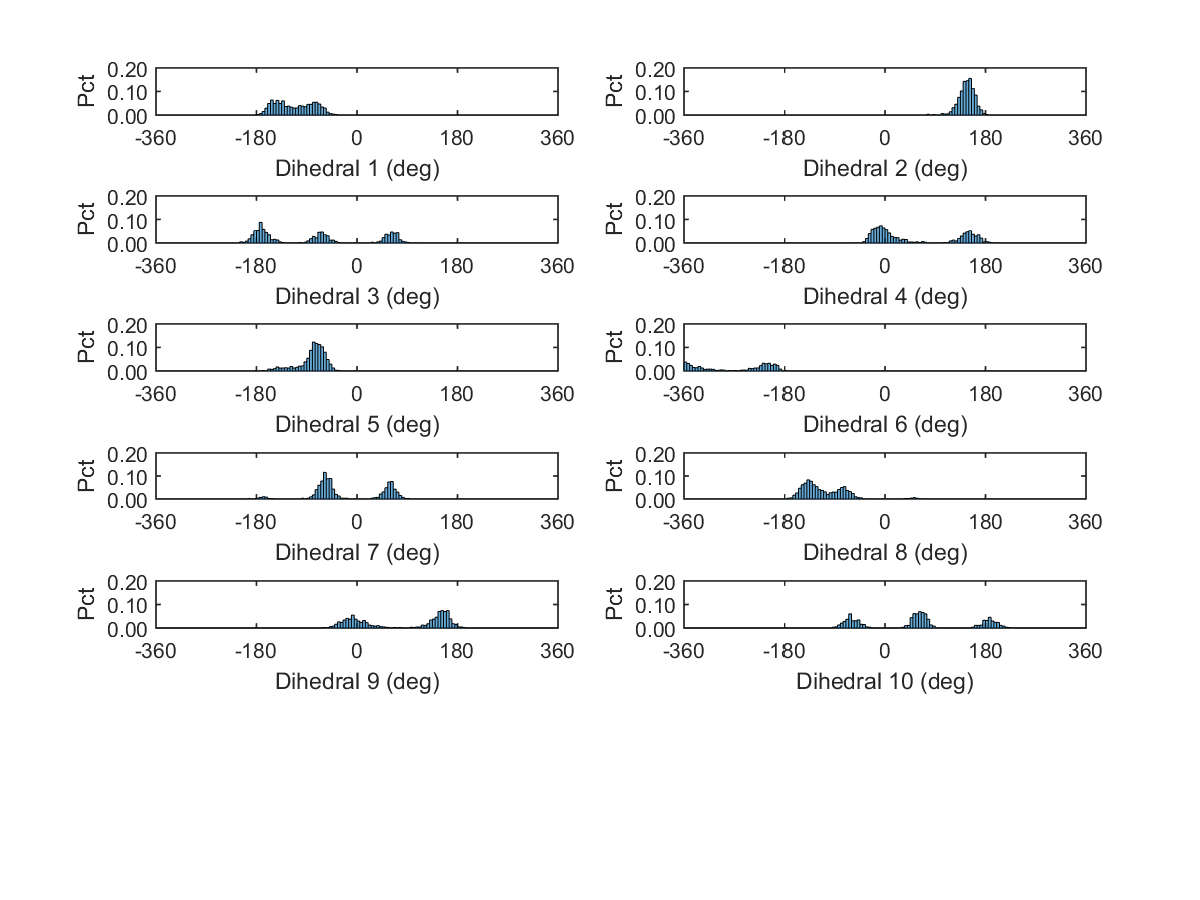

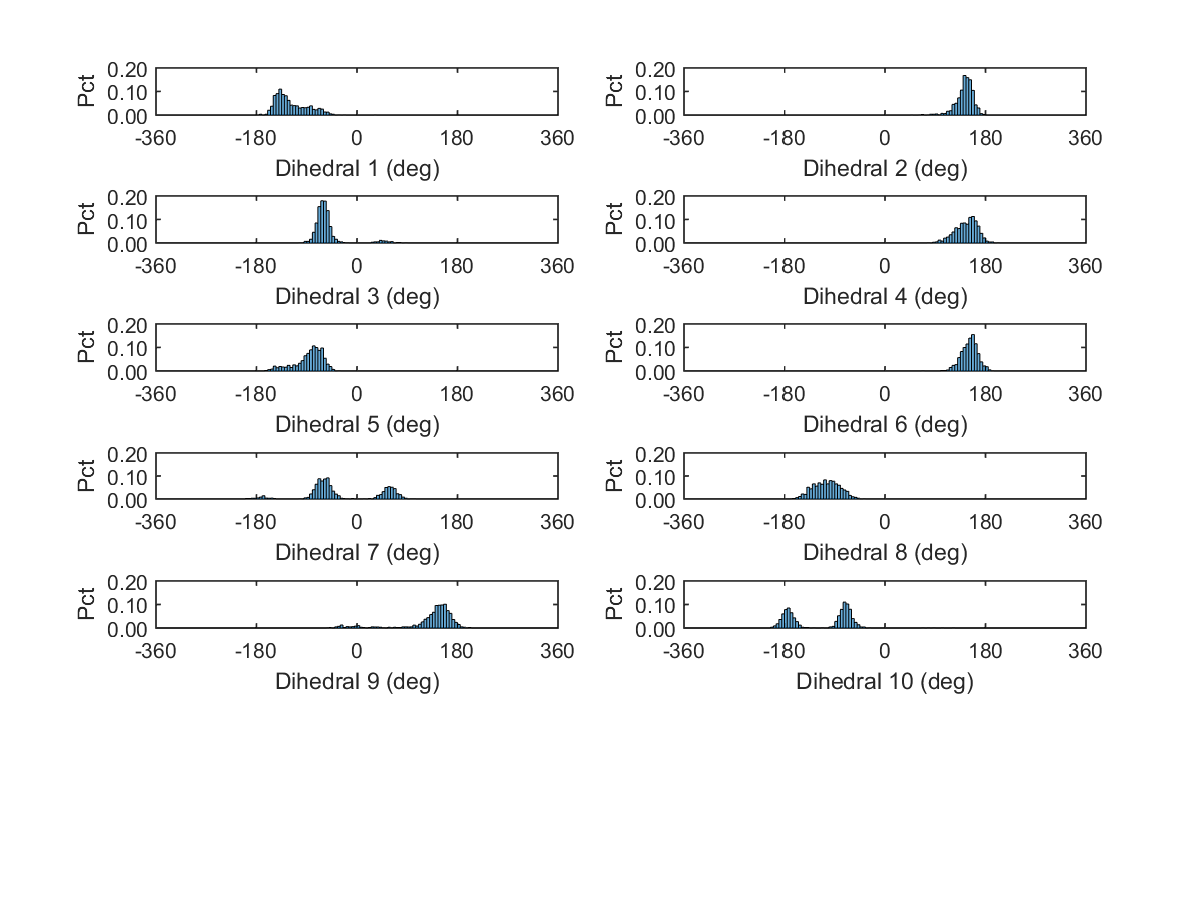


free state from M2 bound state from M2


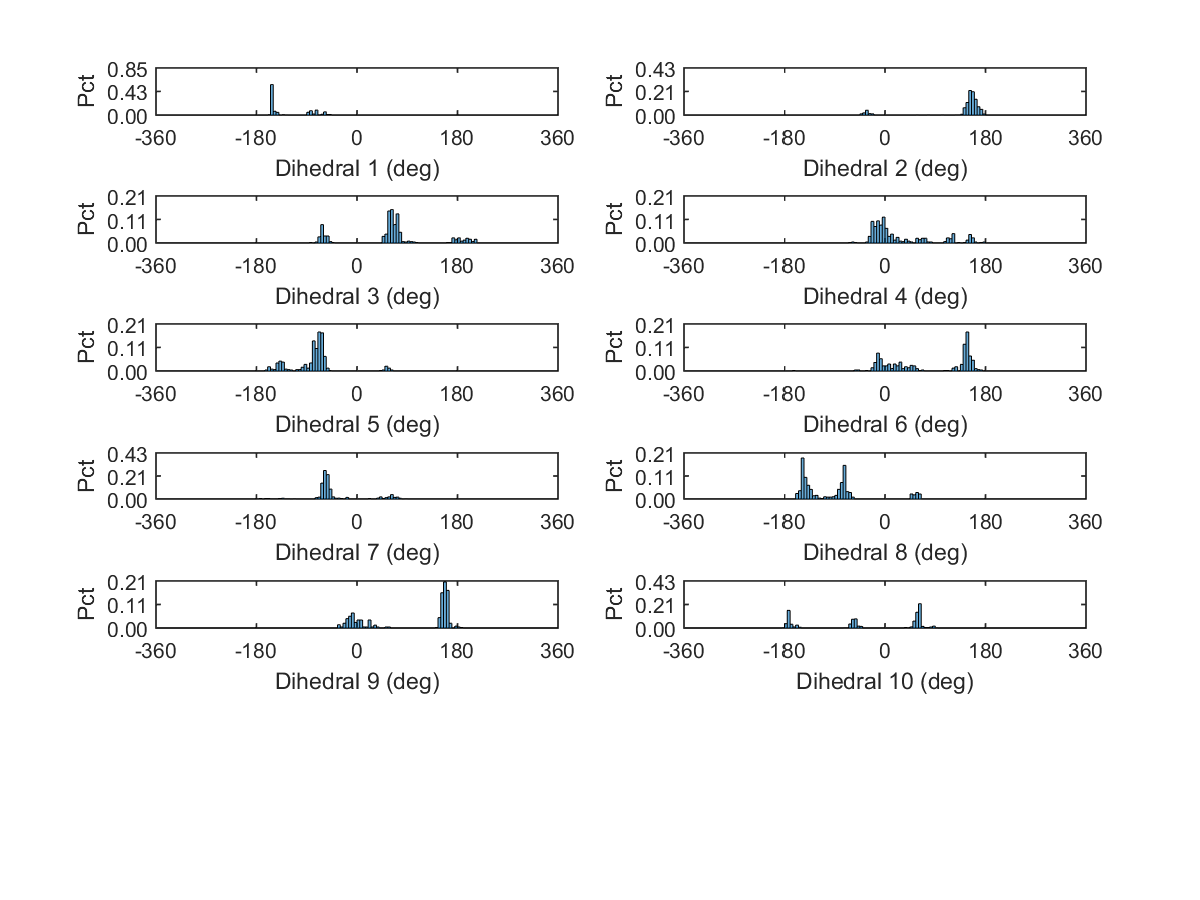

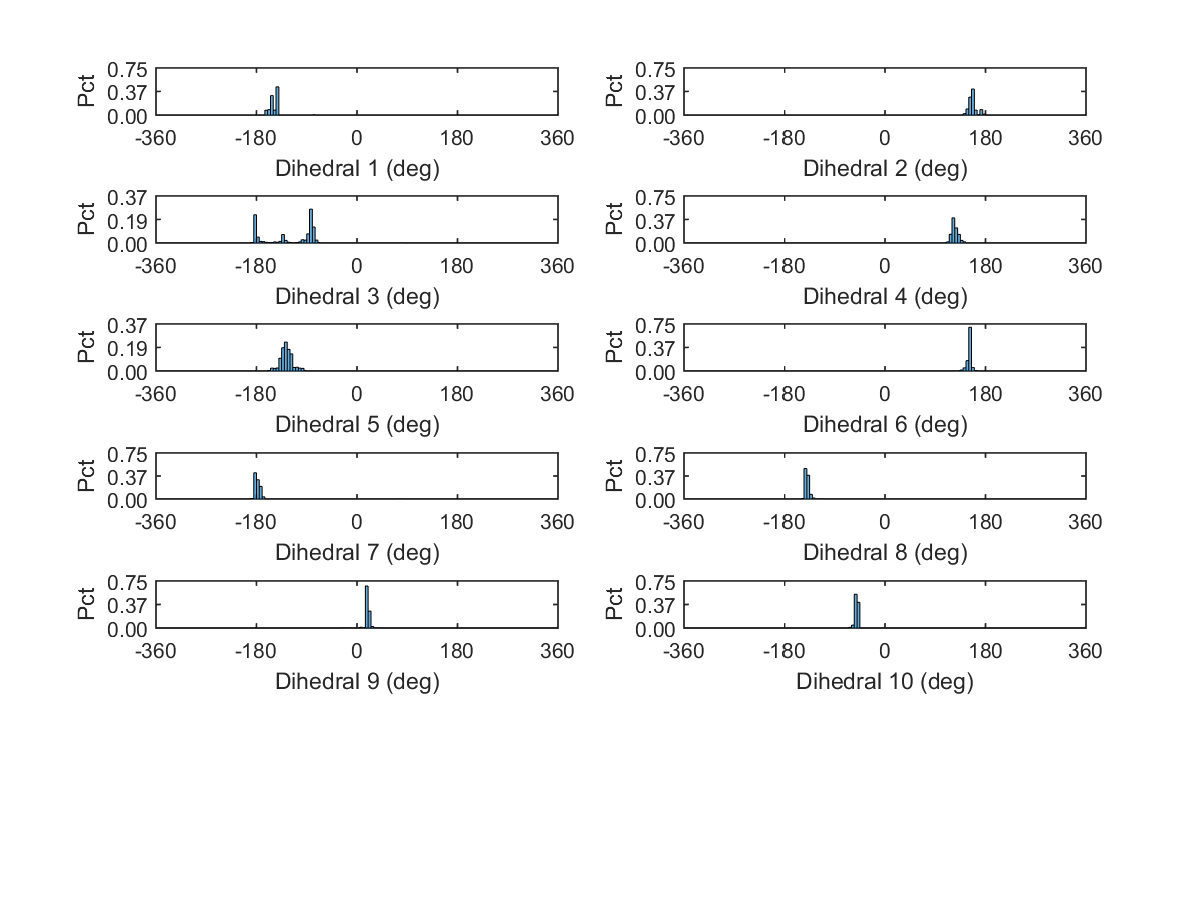


P10

free state from MD bound state from MD


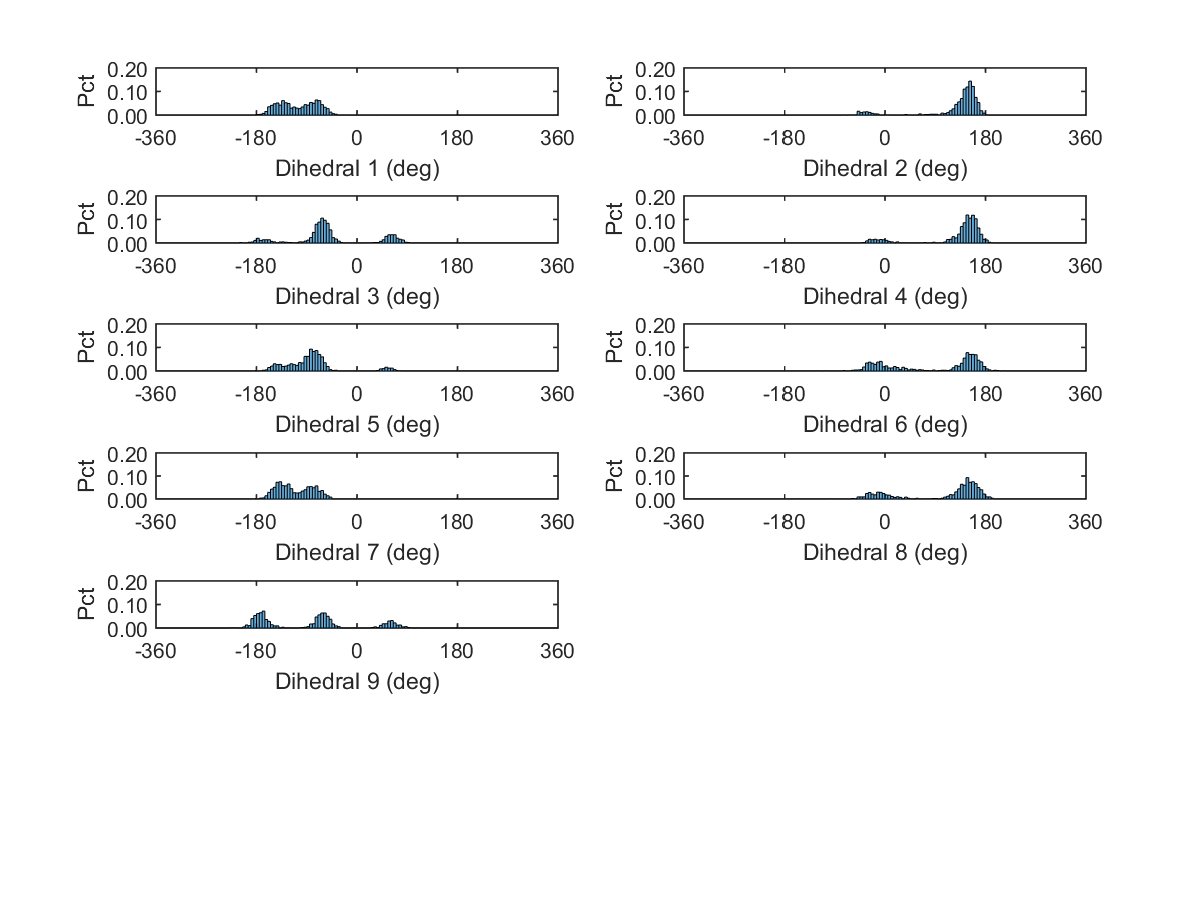

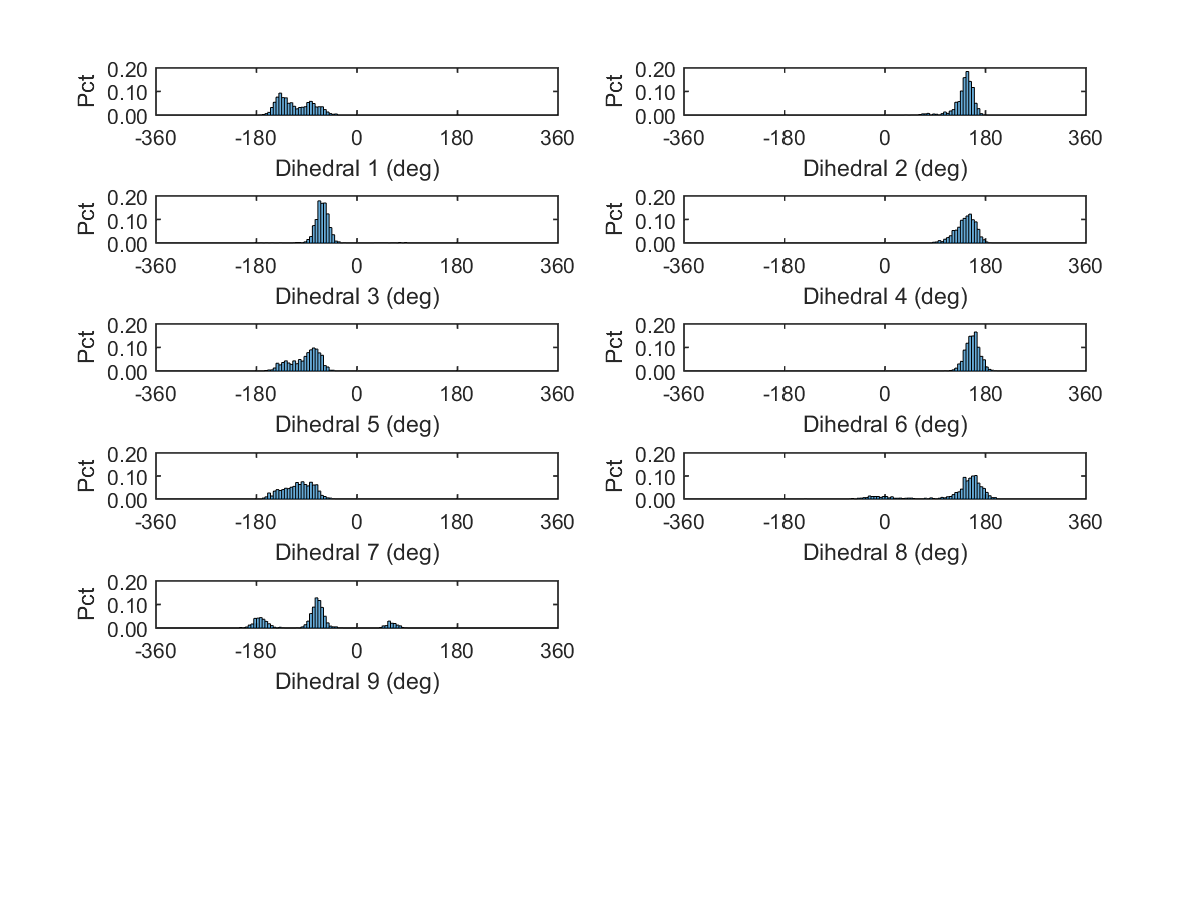


free state from M2 bound state from M2


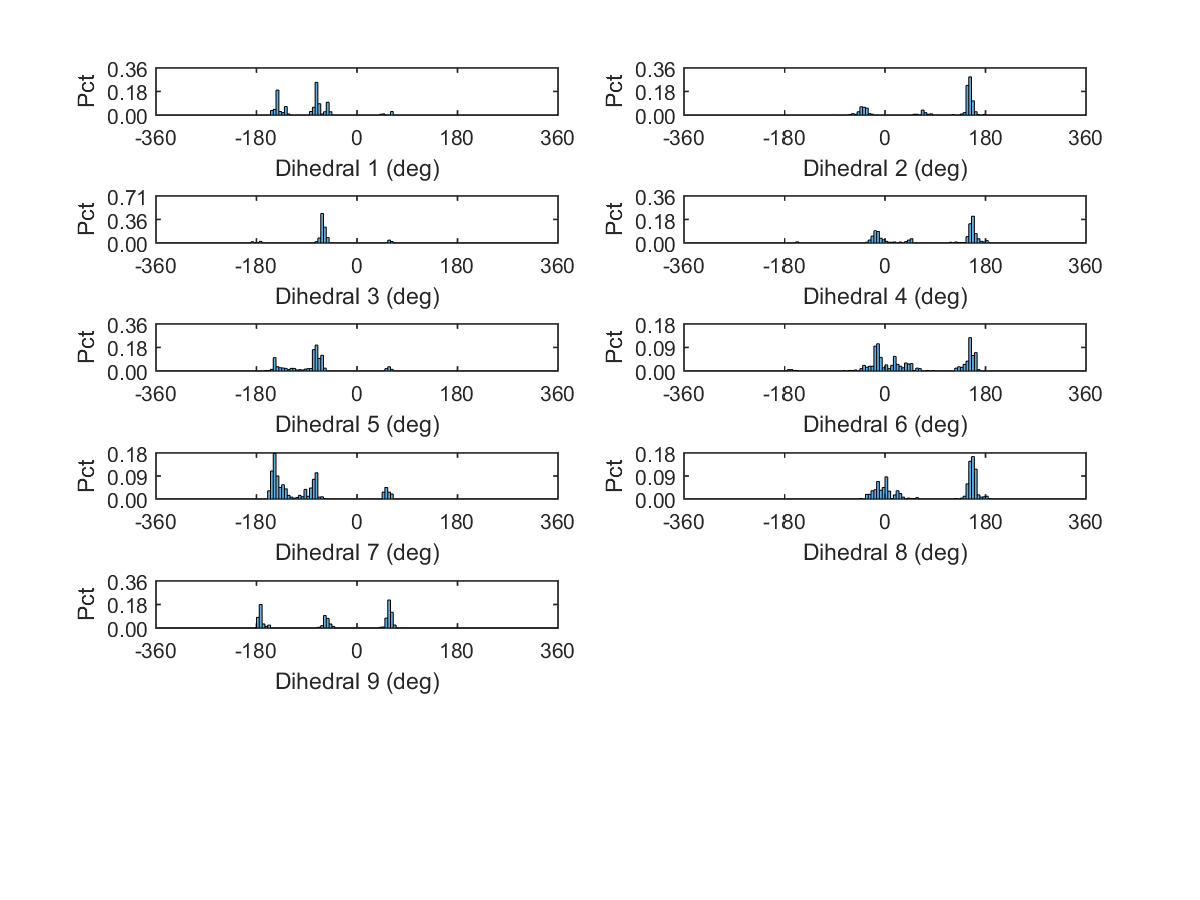

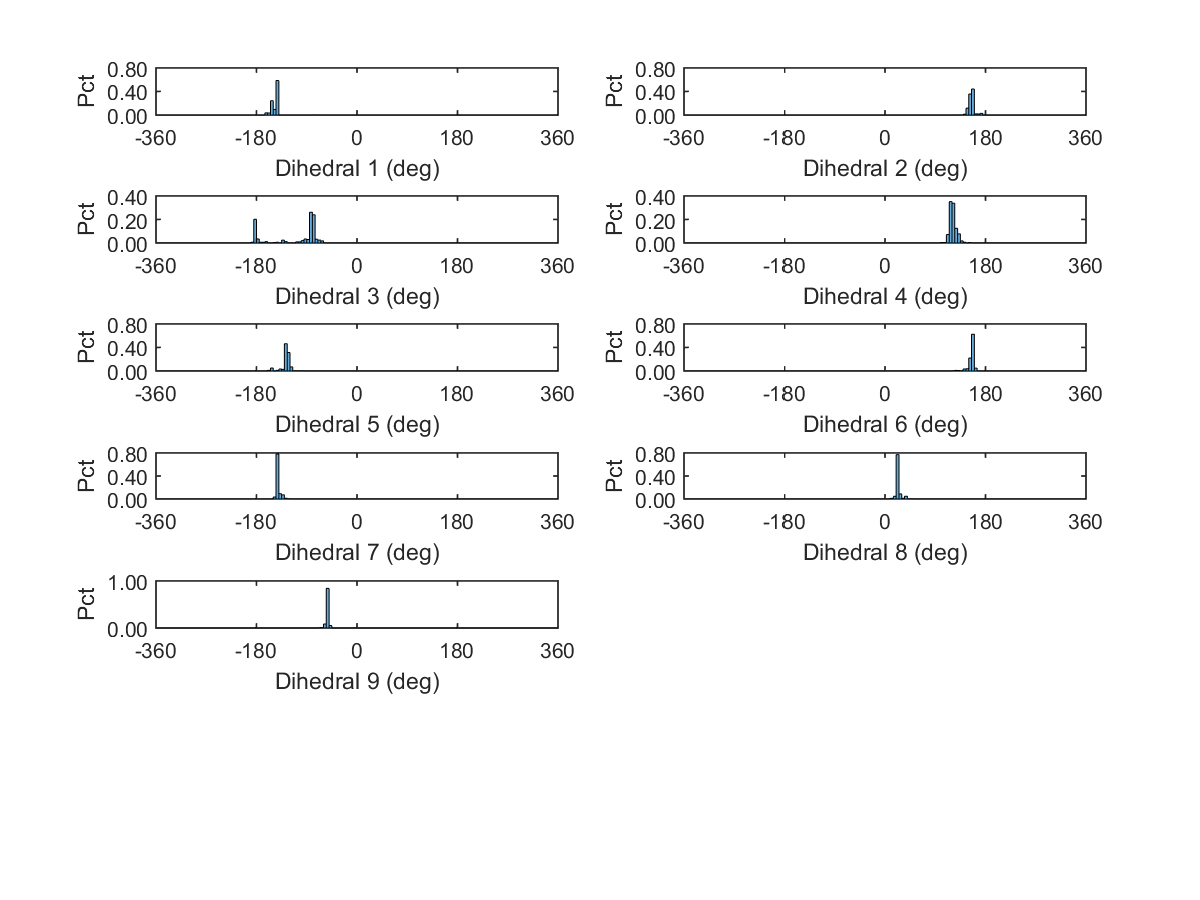


P11

free state from MD bound state from MD


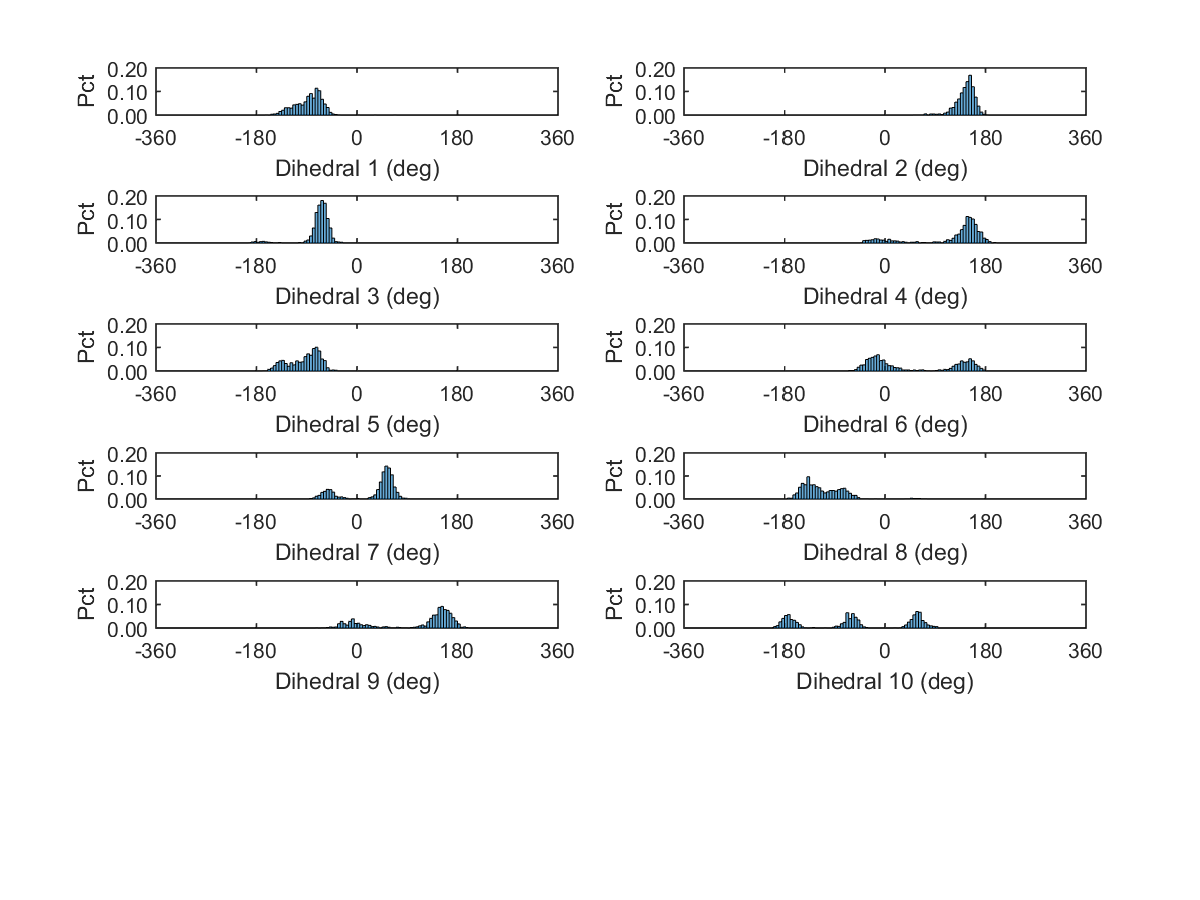

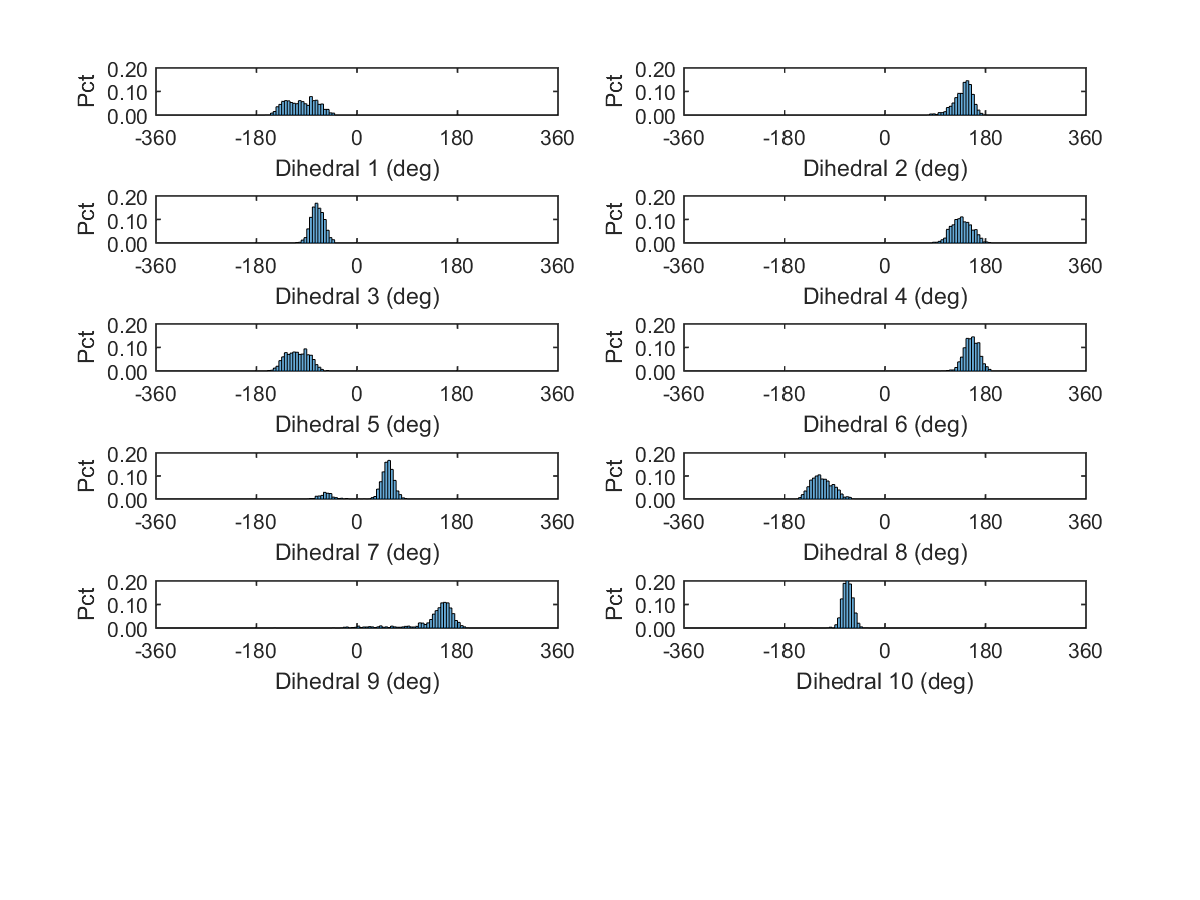


free state from M2 bound state from M2


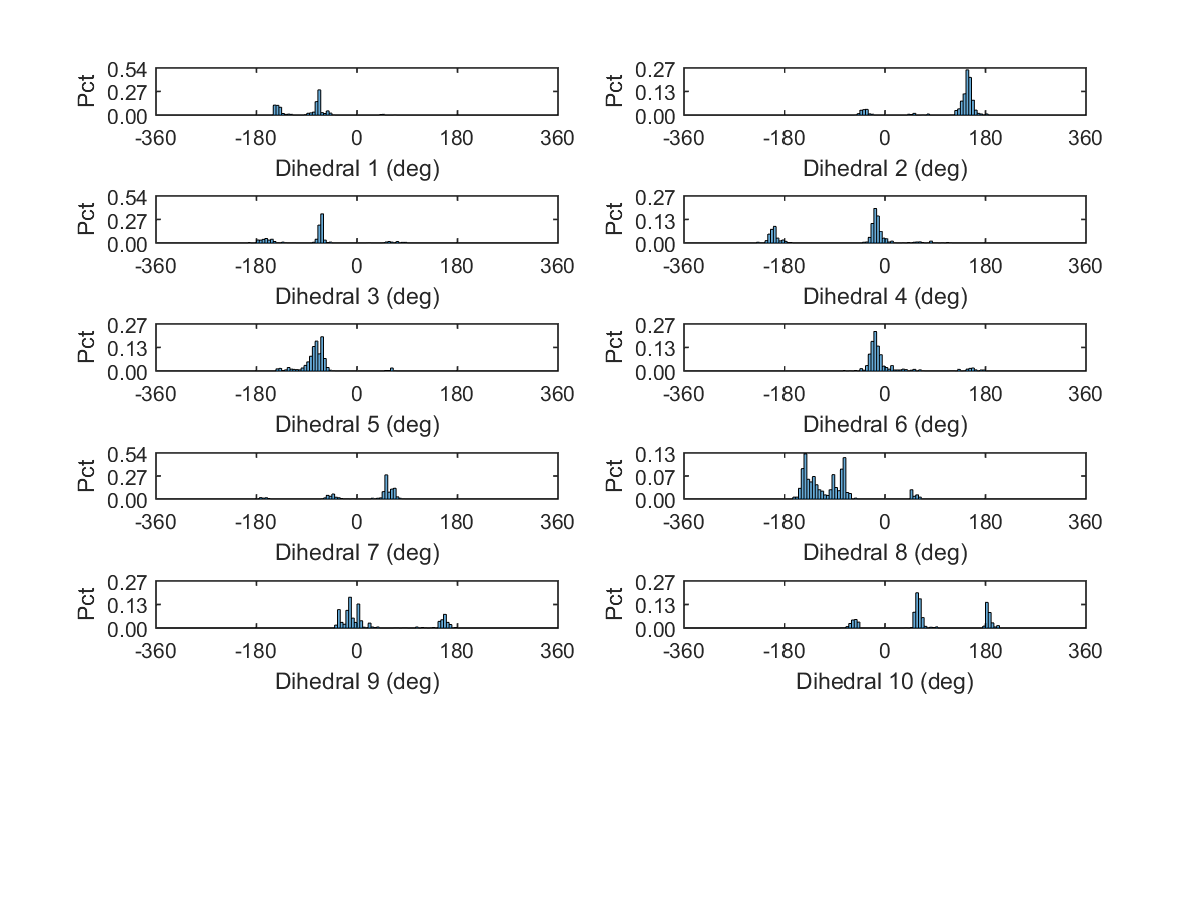

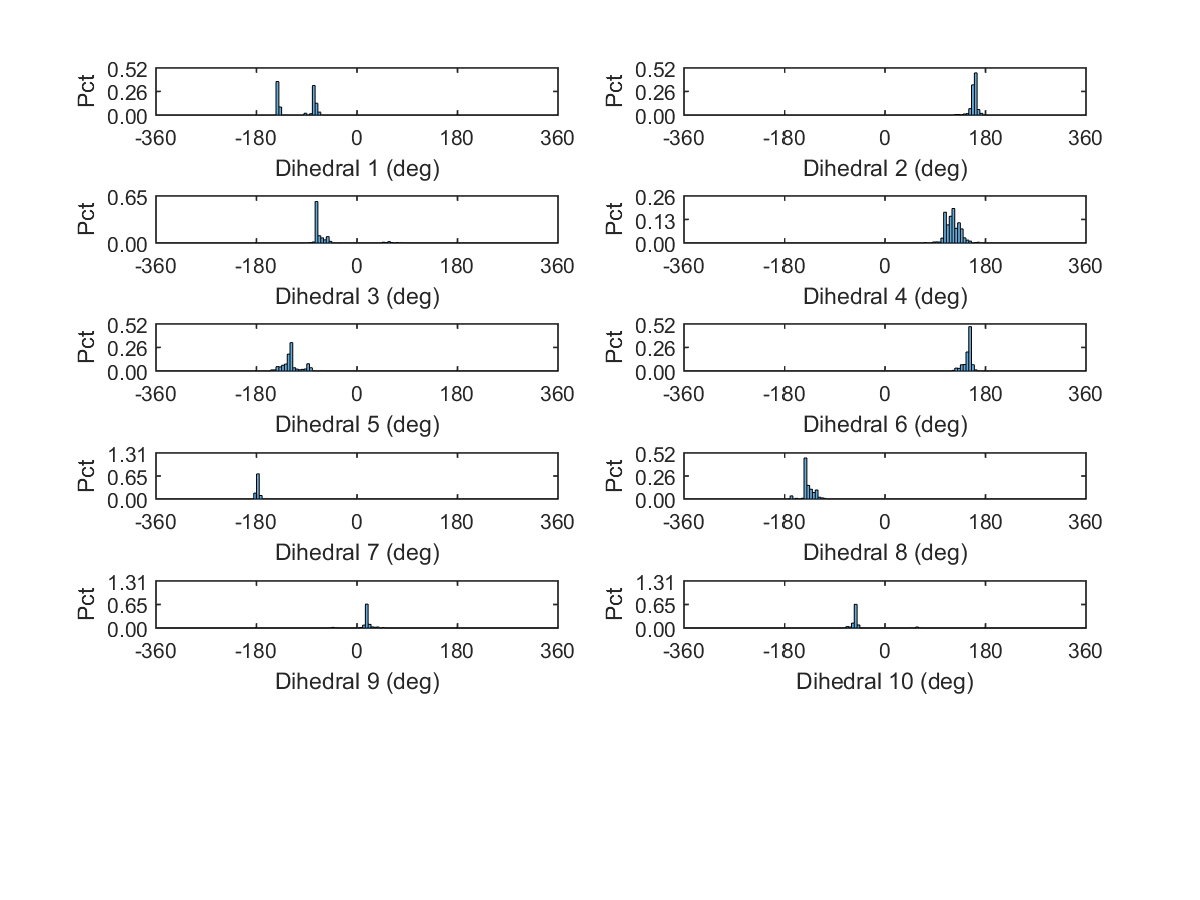


P12

free state from MD bound state from MD


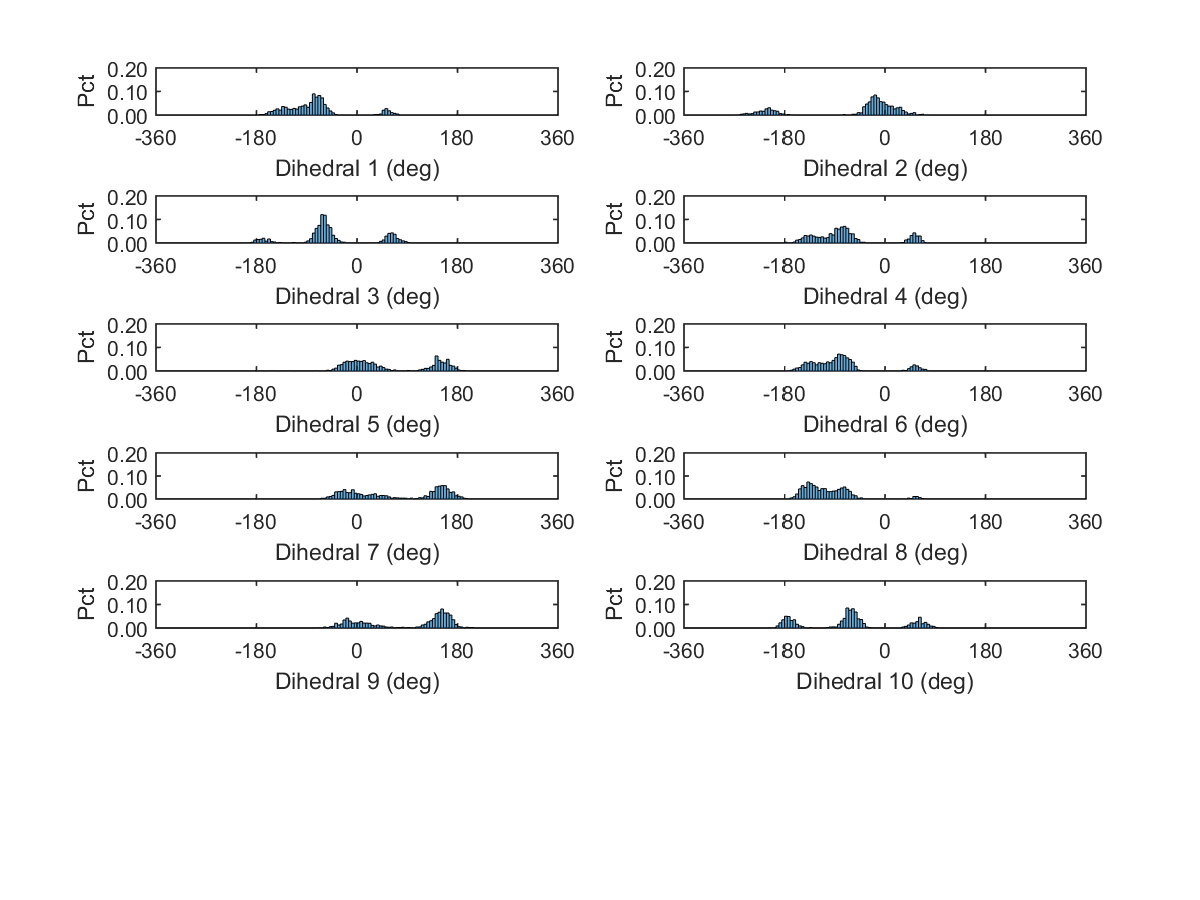

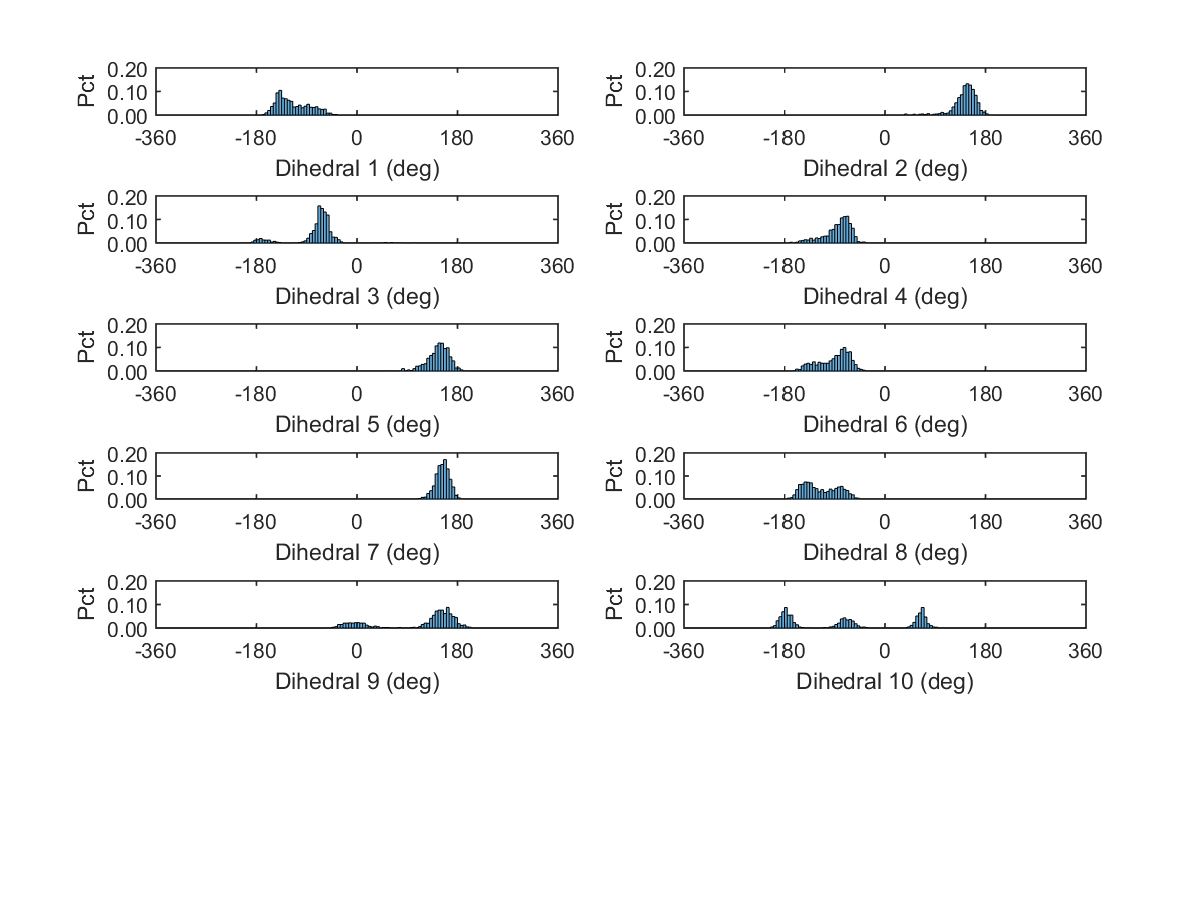


free state from M2 bound state from M2


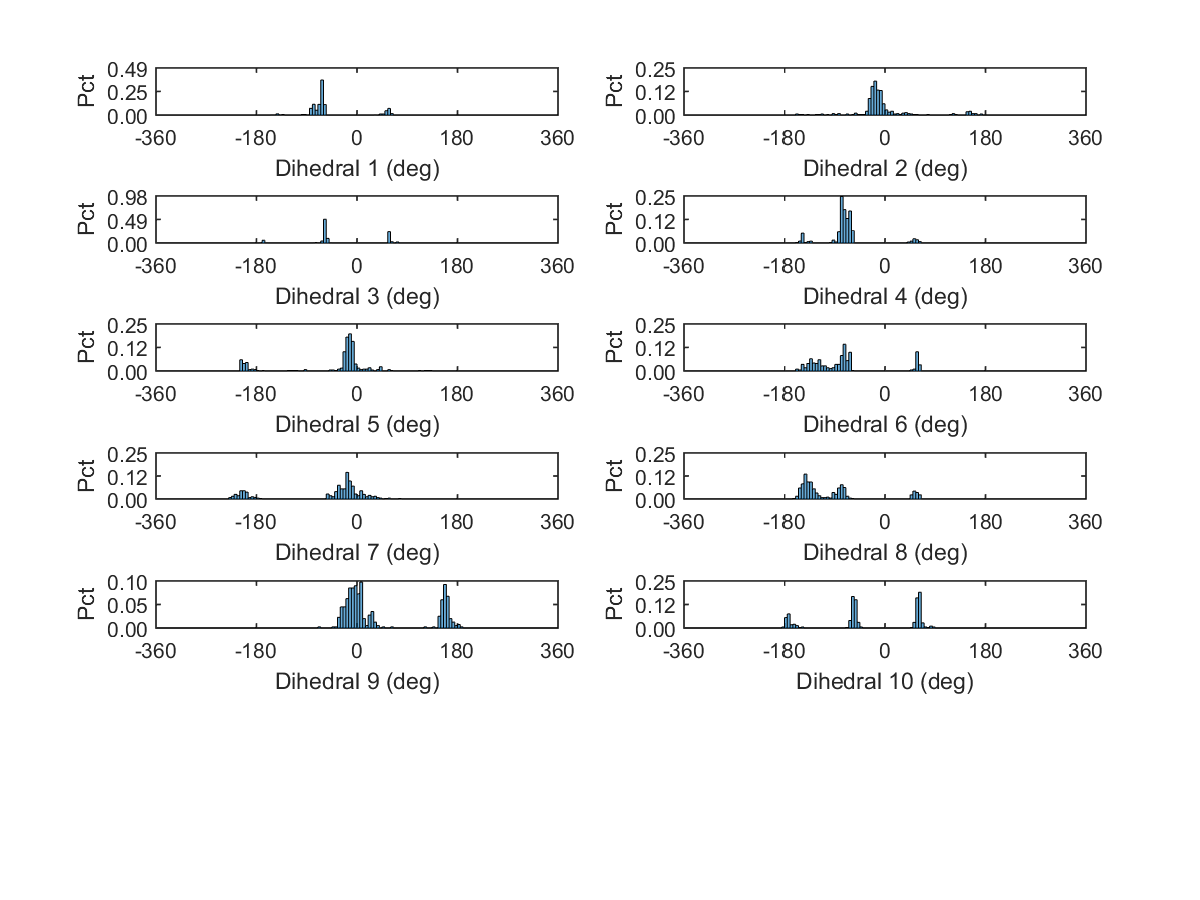

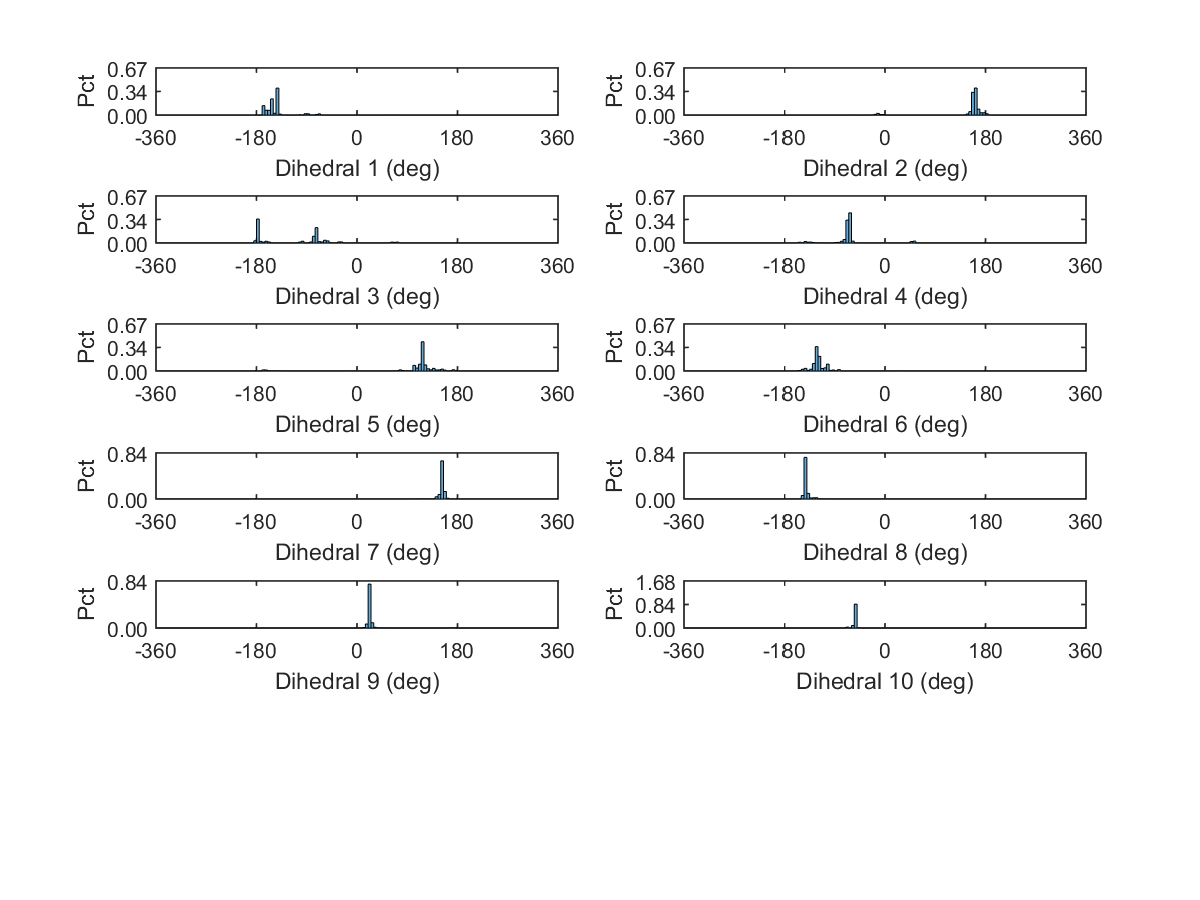


P13

free state from MD bound state from MD


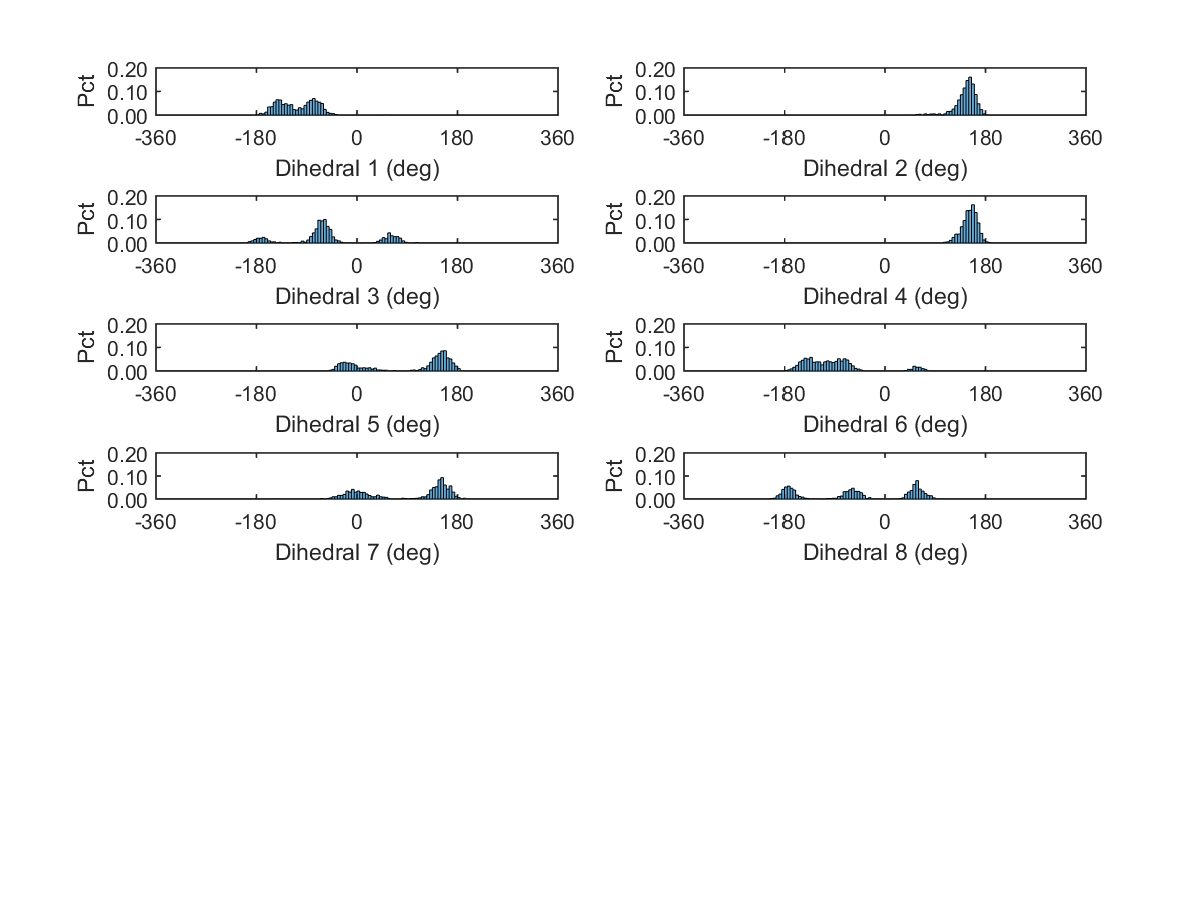

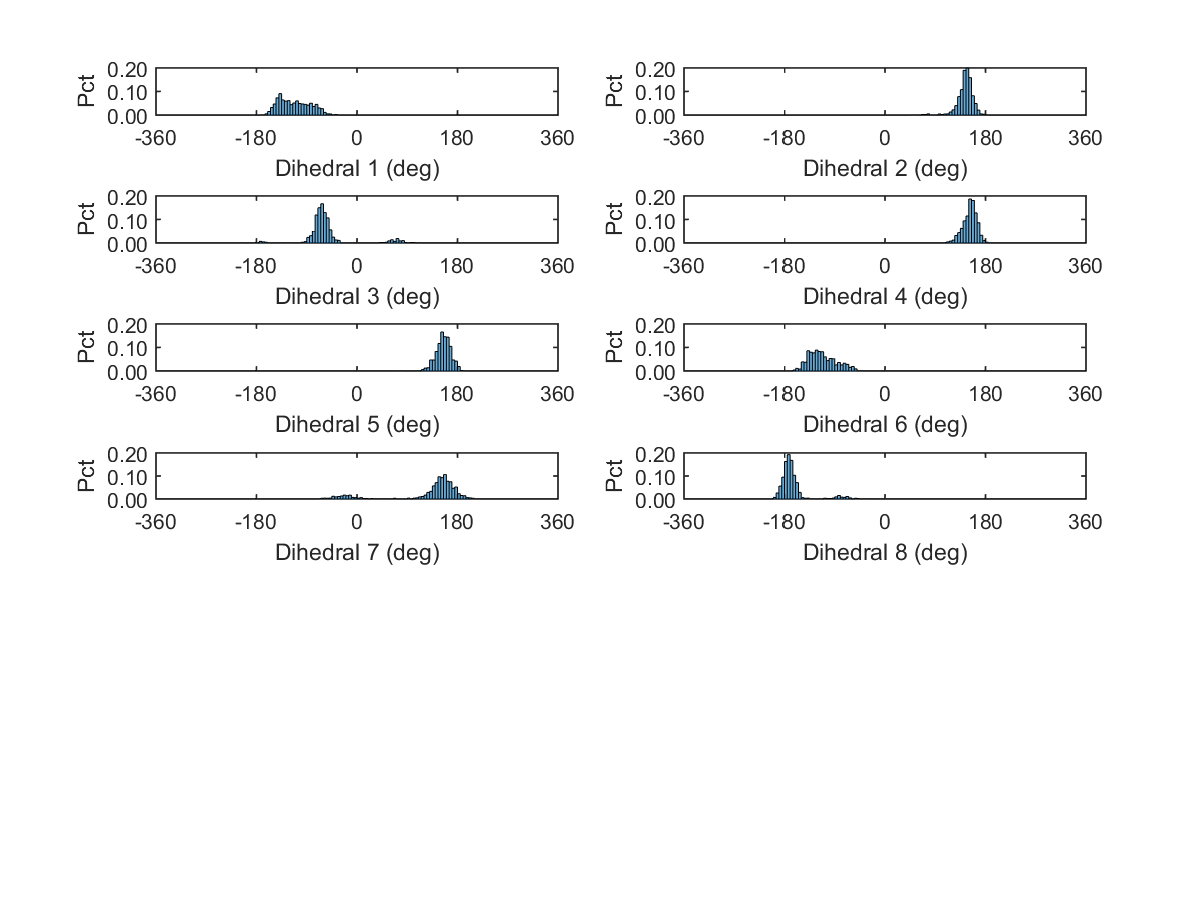


free state from M2 bound state from M2


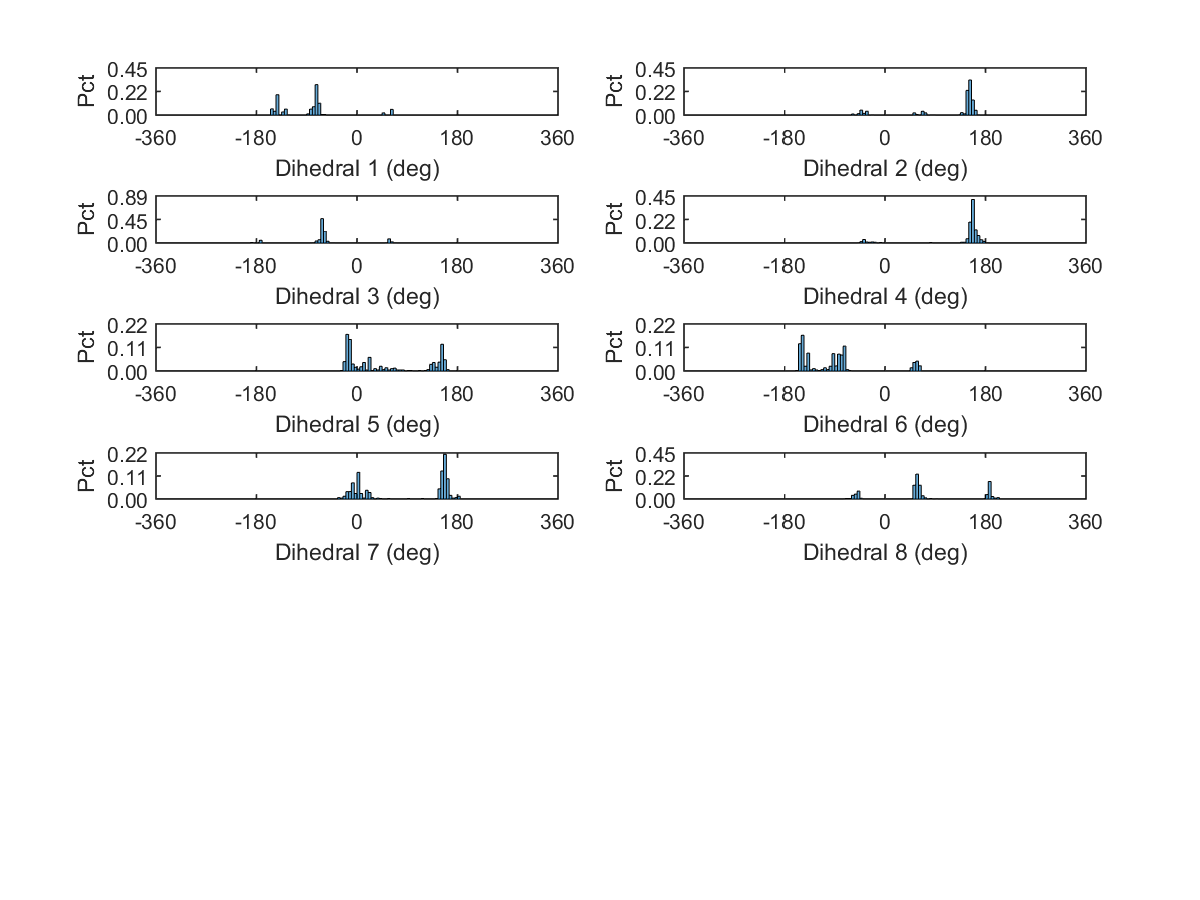

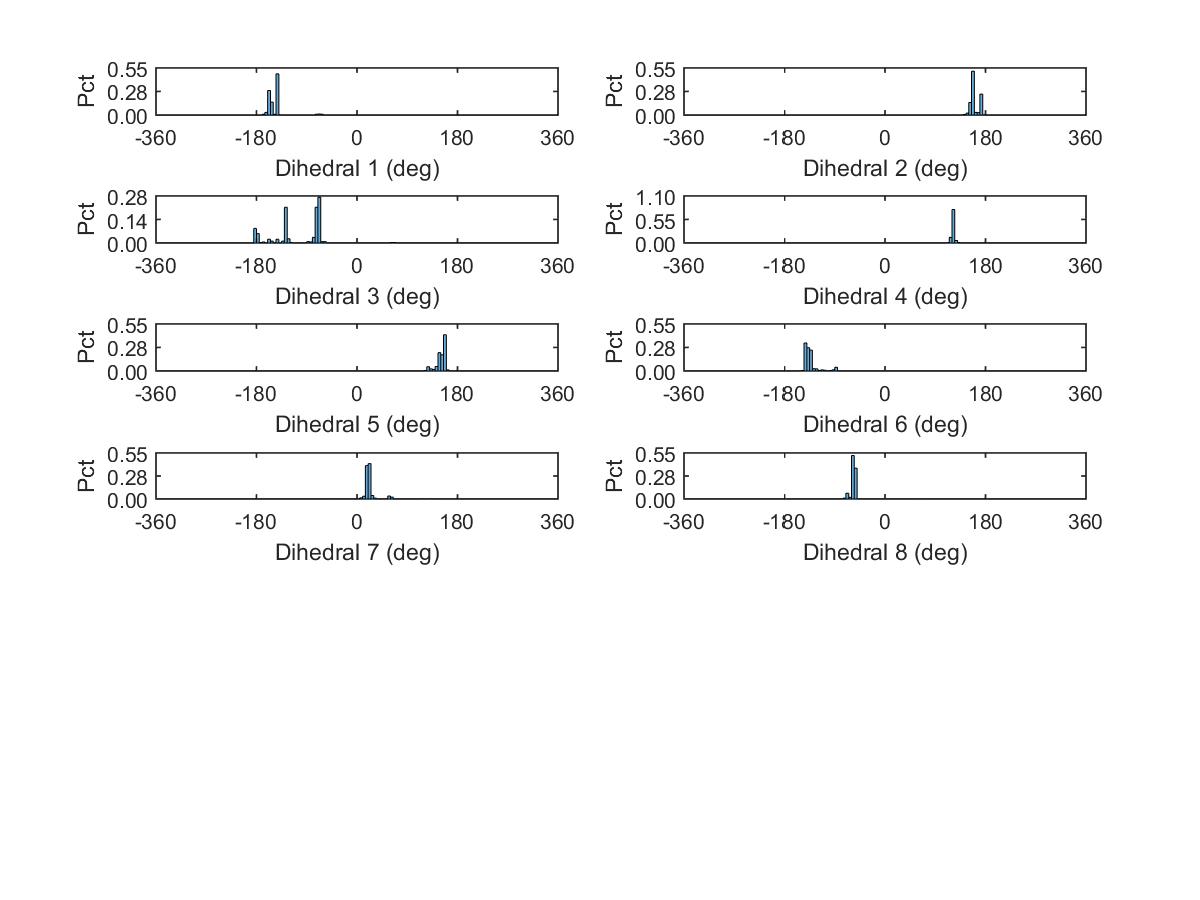


P14

free state from MD bound state from MD


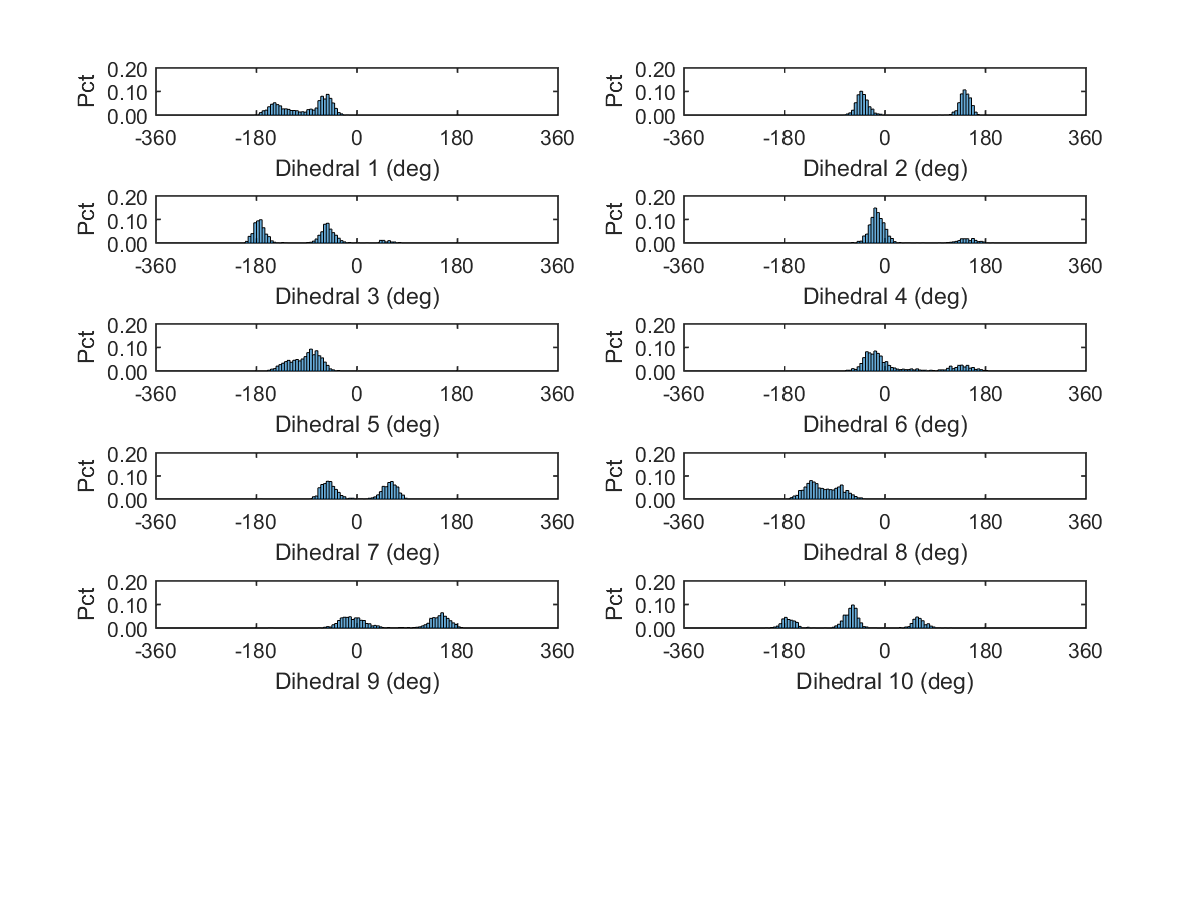

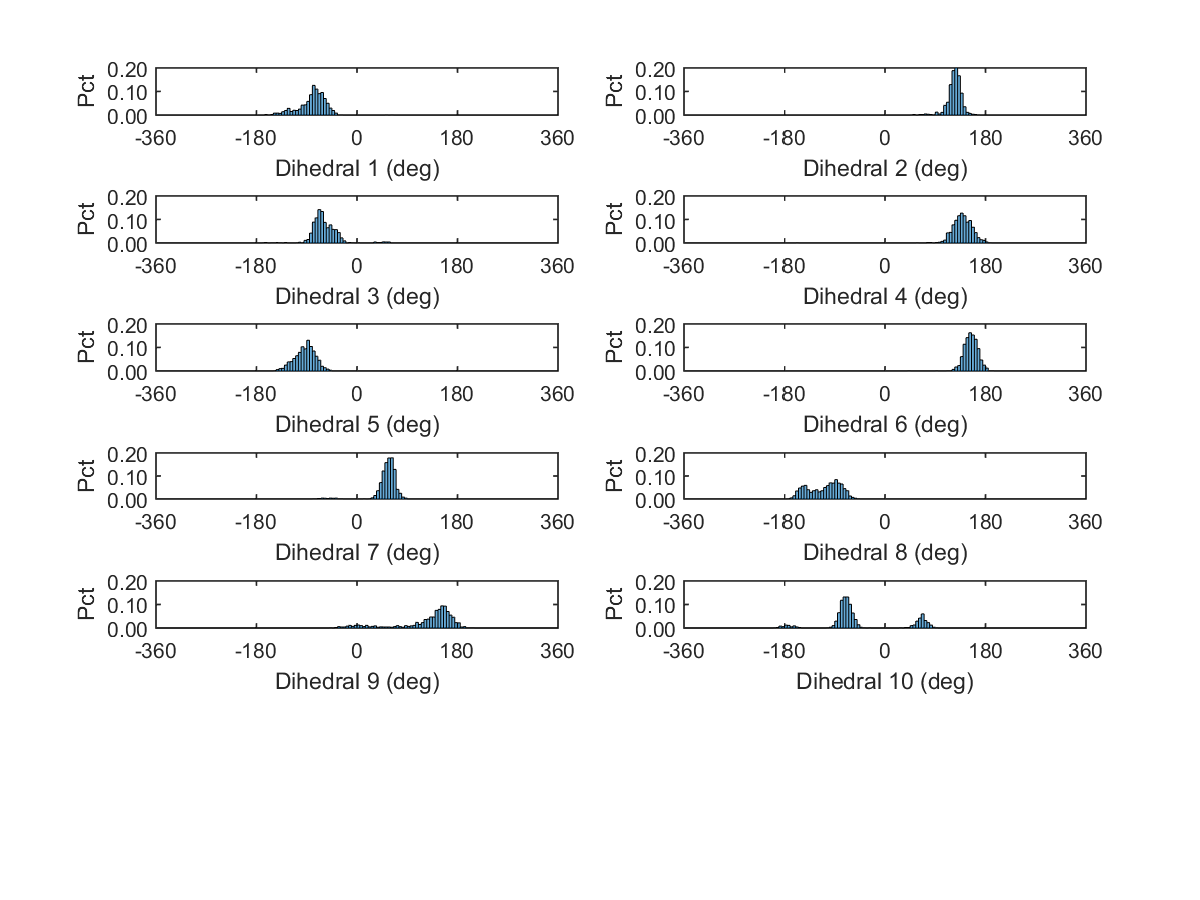


free state from M2 bound state from M2


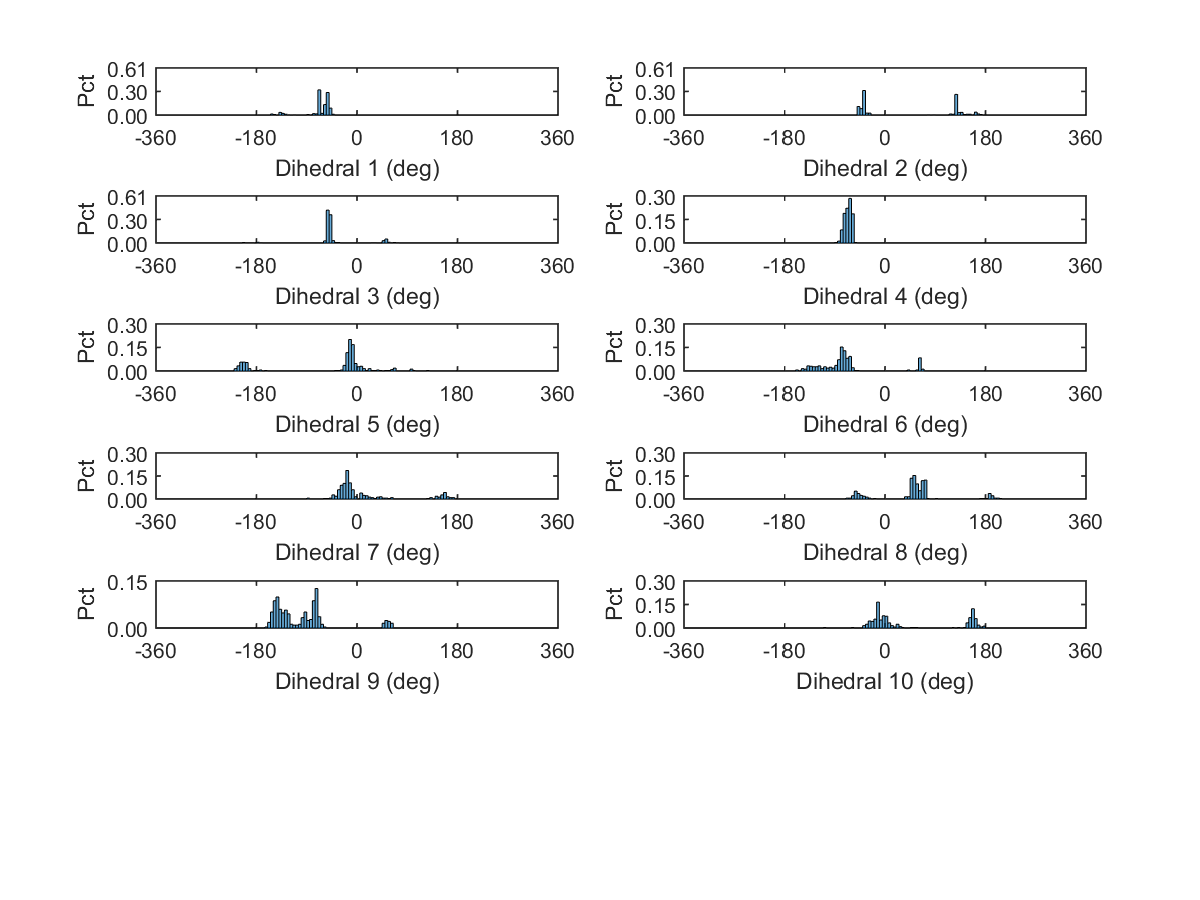

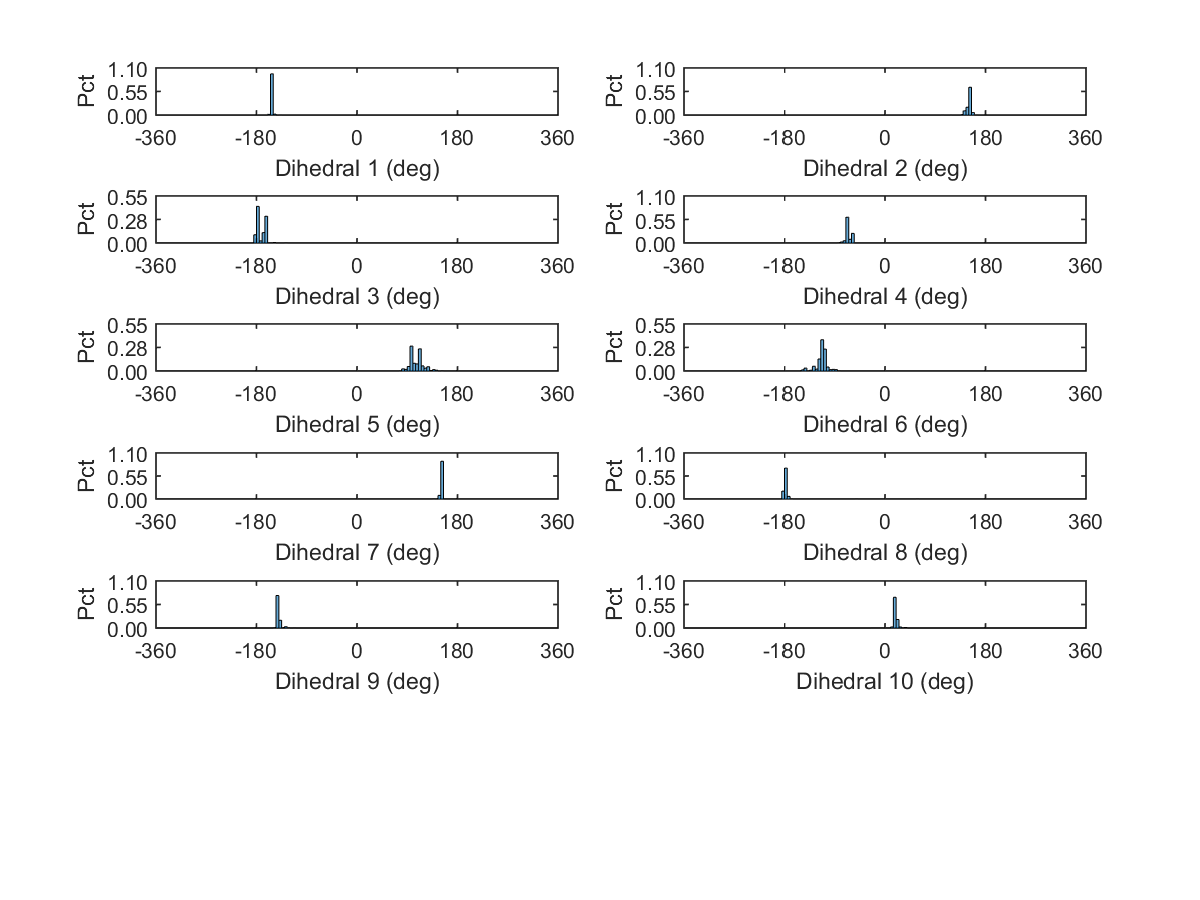


N1

free state from MD bound state from MD


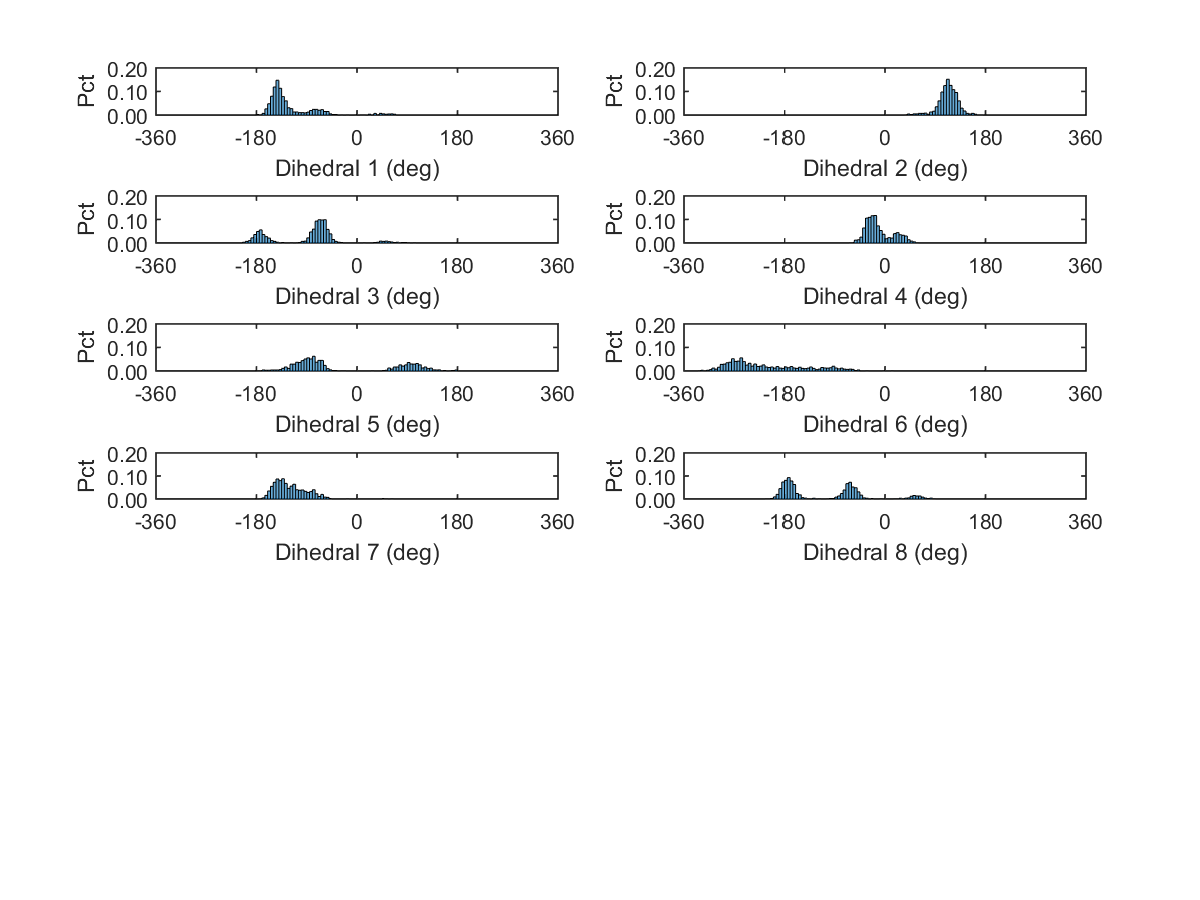

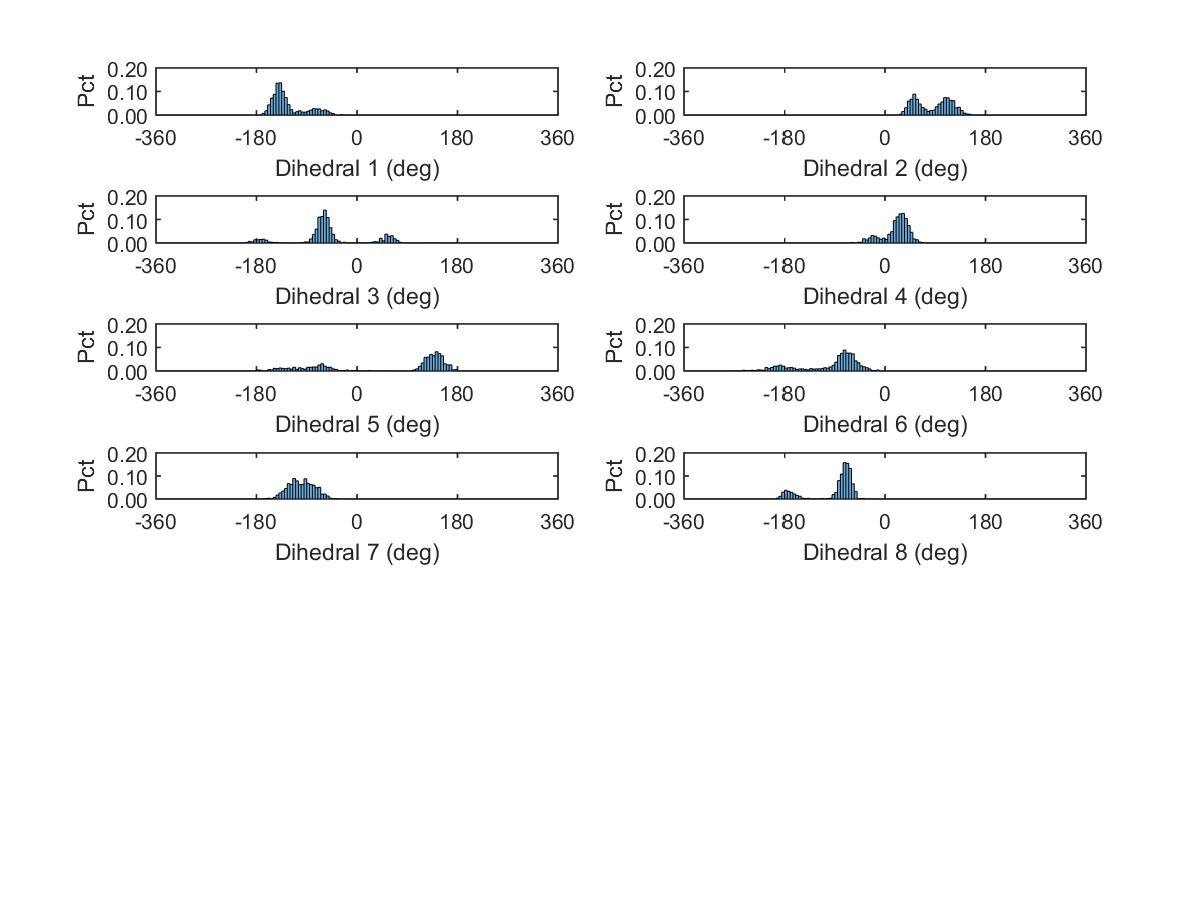


free state from M2 bound state from M2


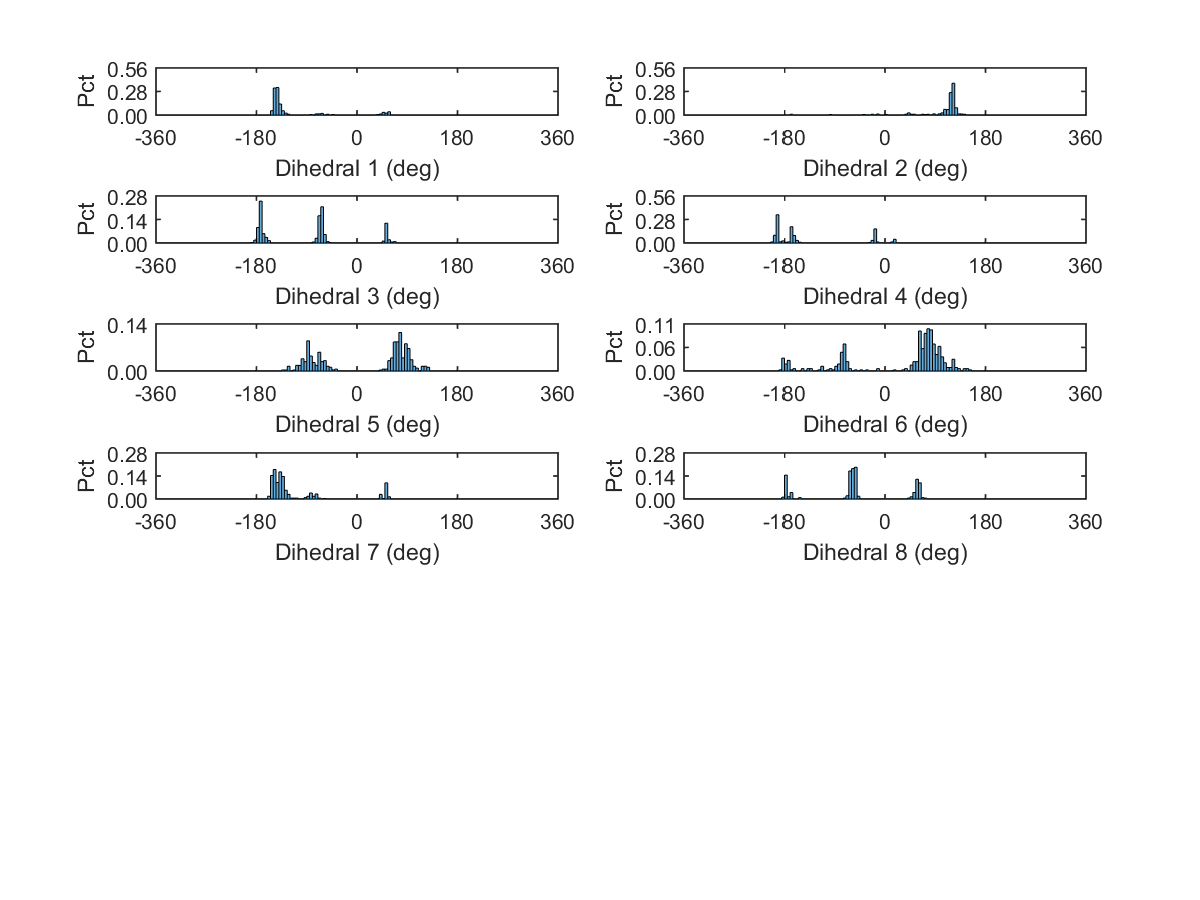

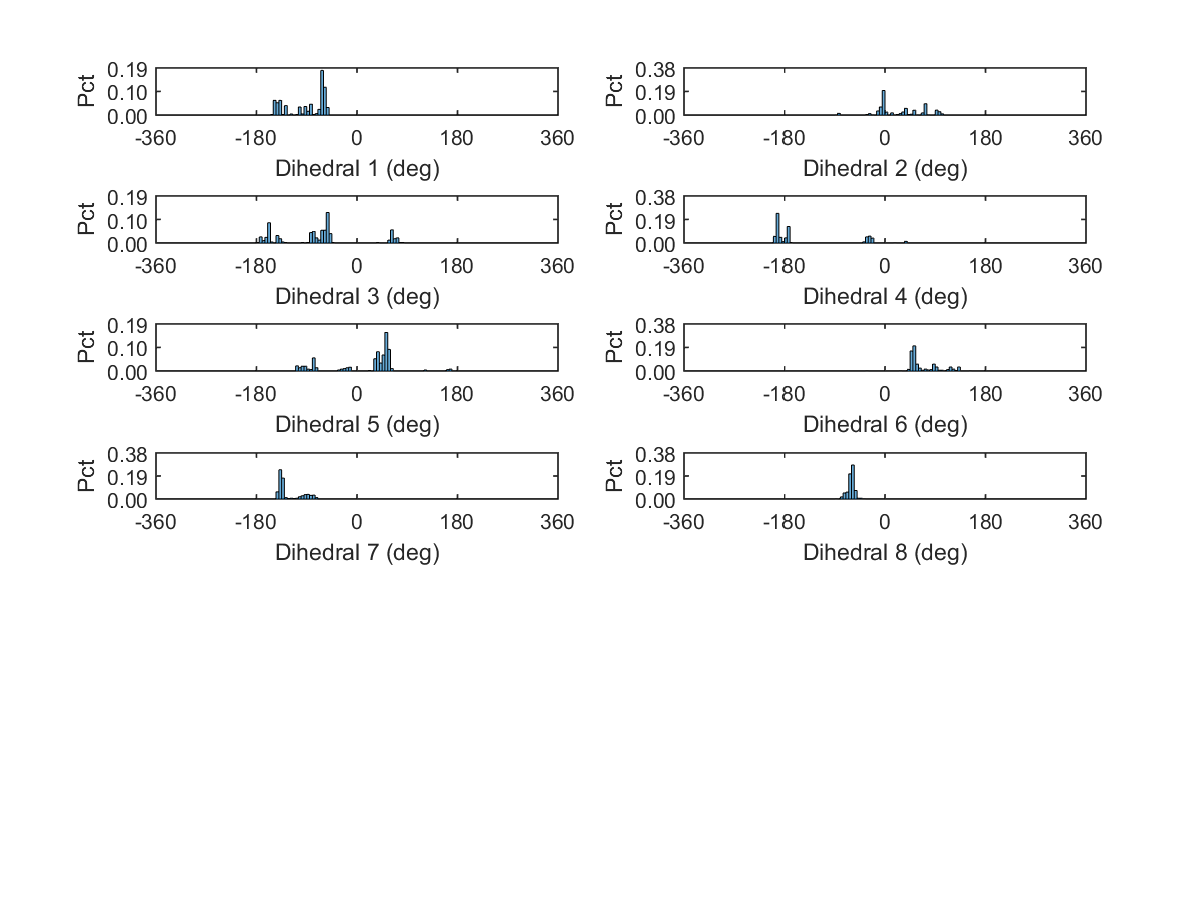


D1

free state from MD bound state from MD


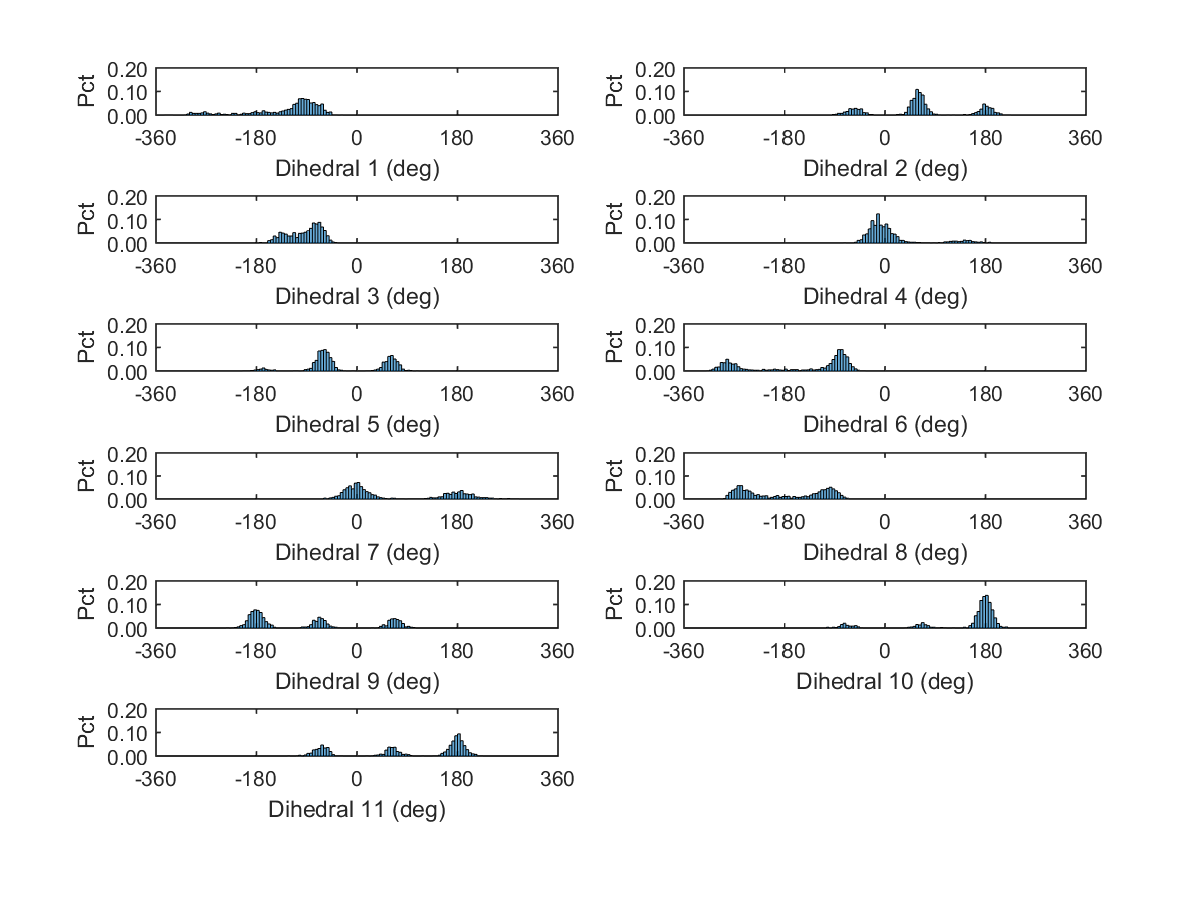

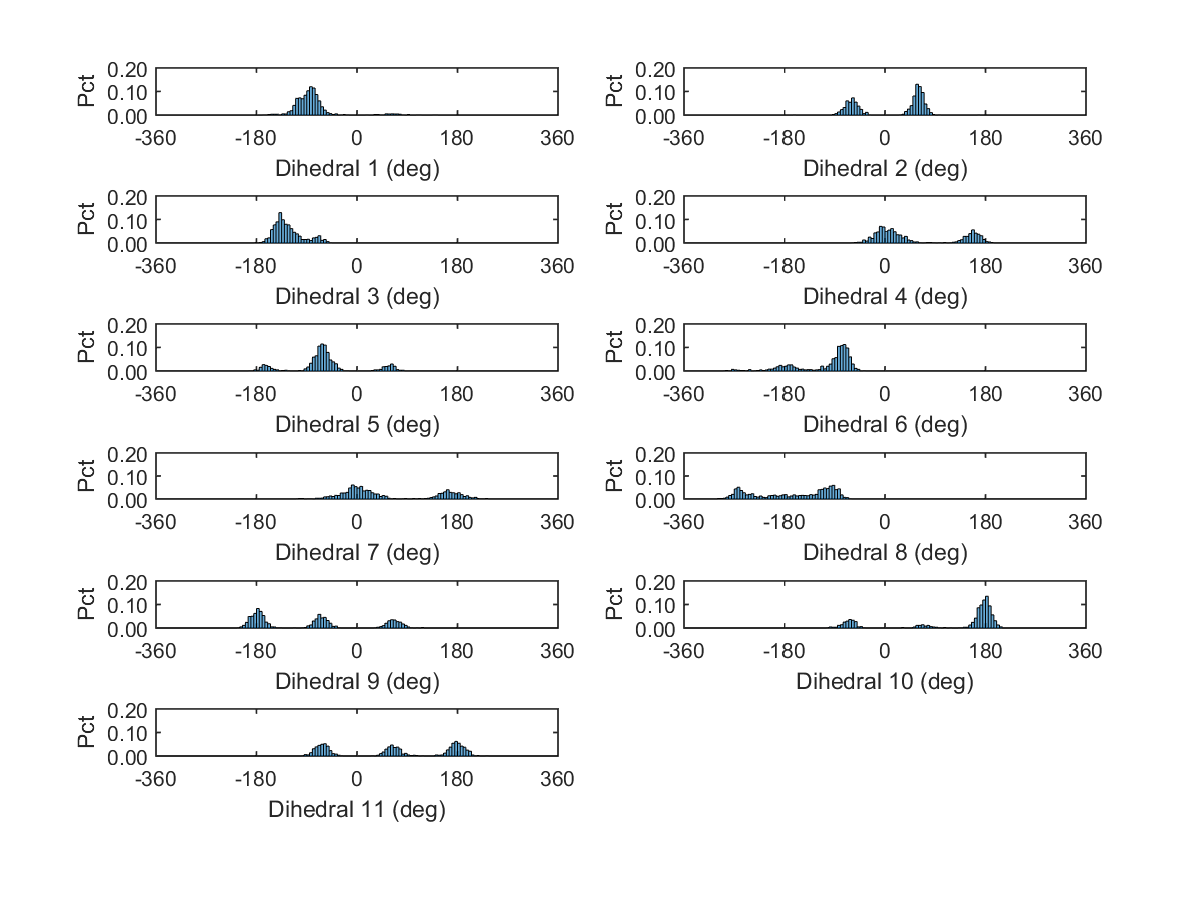


free state from M2 bound state from M2


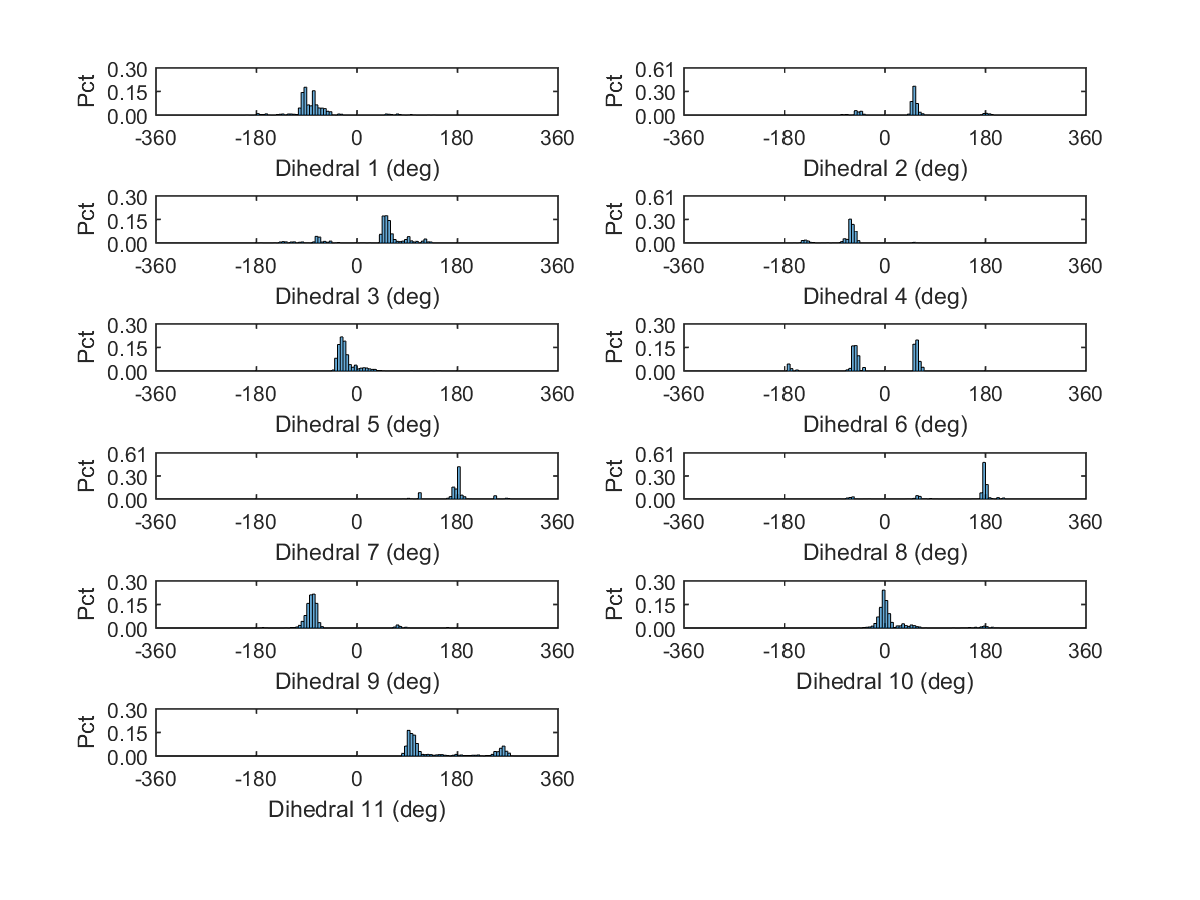

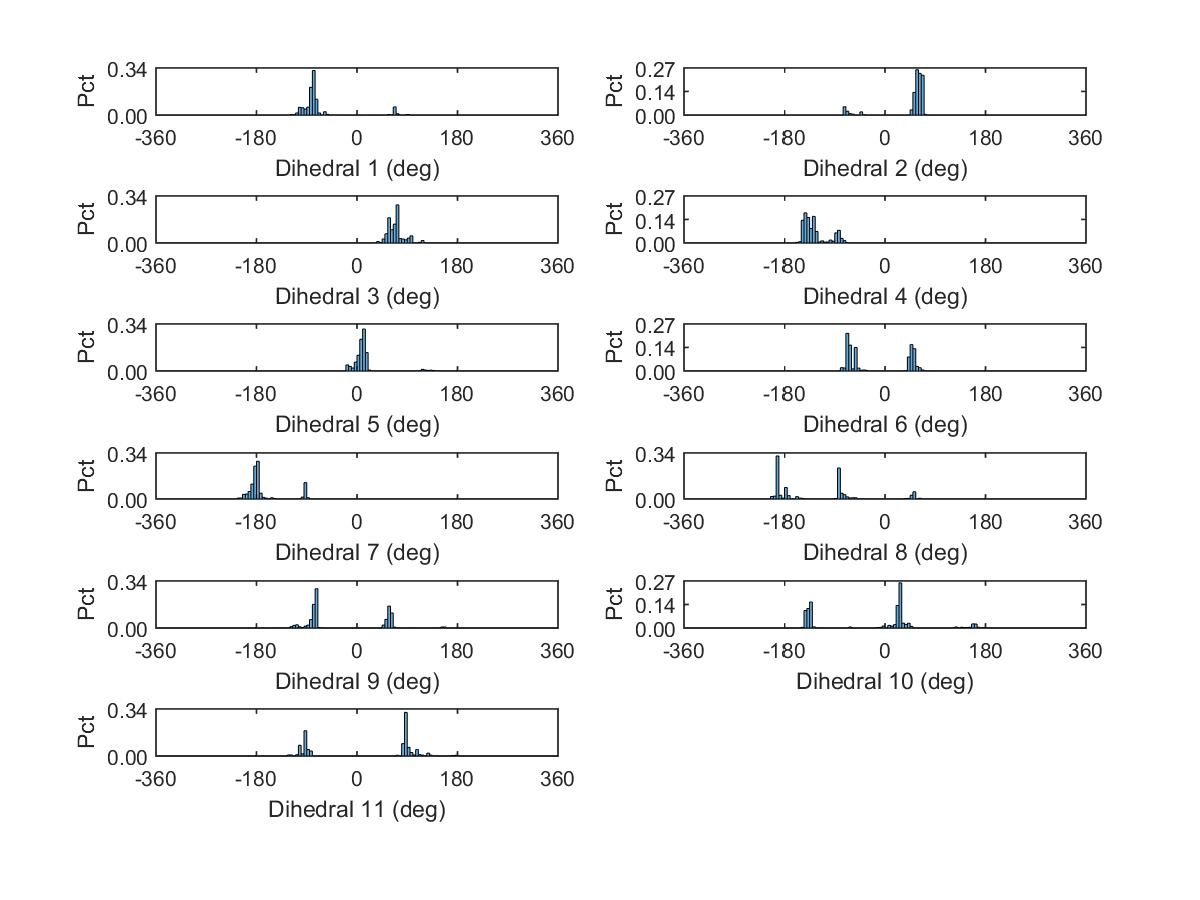

Supplement: S7 Fig — (DOCX) [file pcbi.1005057.s010.docx]
