# Supplementary material for: Characterization of Promiscuous Binding of Phosphor Ligands to Breast-Cancer-Gene 1 (BRCA1) C-Terminal (BRCT): Molecular Dynamics, Free Energy, Entropy and Inhibitor Design
Source: PLoS Comput Biol. 2016 Aug 25;12(8):e1005057. doi: 10.1371/journal.pcbi.1005057 (PMC4999267; doi:10.1371/journal.pcbi.1005057)

**S8 Fig. Comparison of the first side-chain dihedral angles of part of live set residues of C1 and P4-BRCT complex from MD and M2, respectively.** (A1), (A2). The first side-chain dihedral angles of part of live set residues of C1 and P4-BRCT complexes from MD, respectively. (B1), (B2). The first side-chain dihedral angles of part of live set residues of C1 and P4-BRCT complexes from M2, respectively. The difference is highlighted by red circle.

(A1)

The first side-chain dihedral angles of part of live set residues of C1-BRCT complex from MD


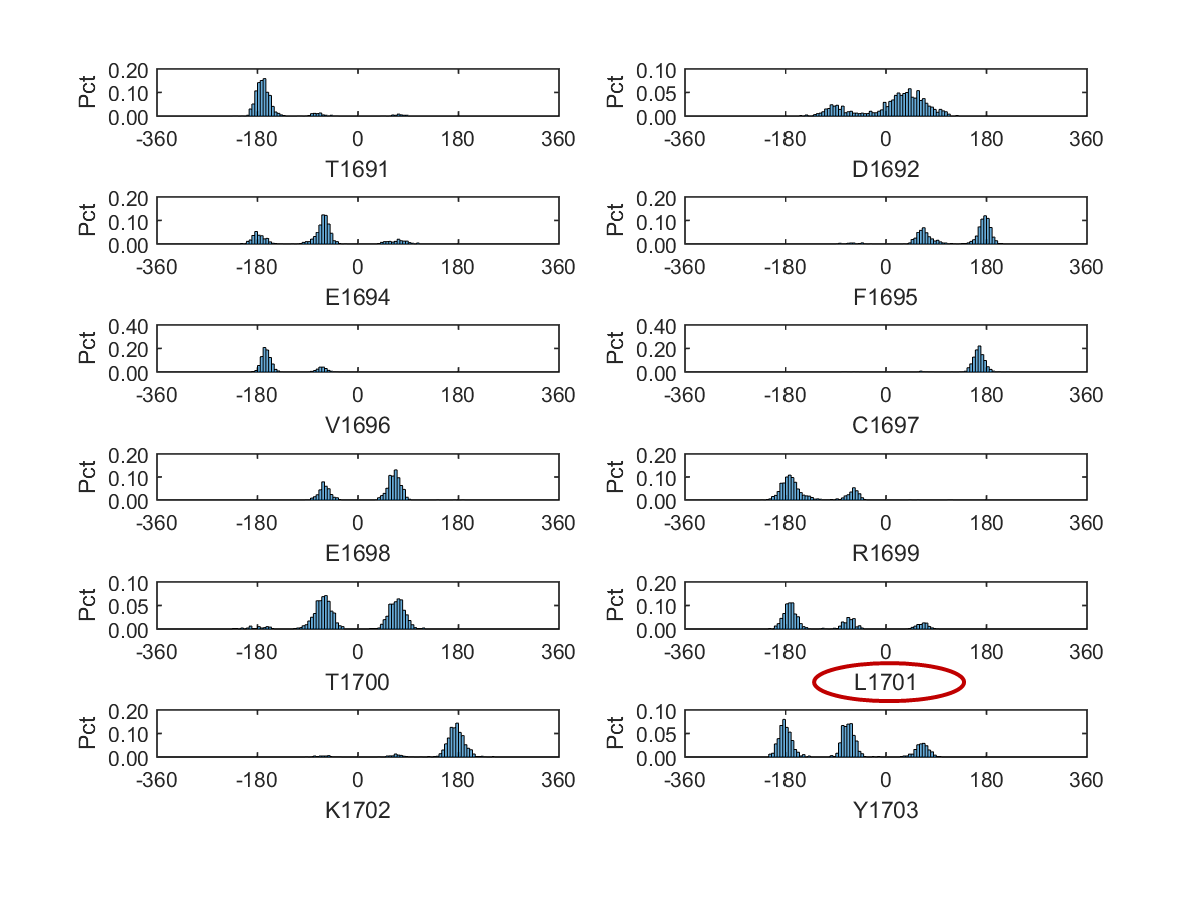

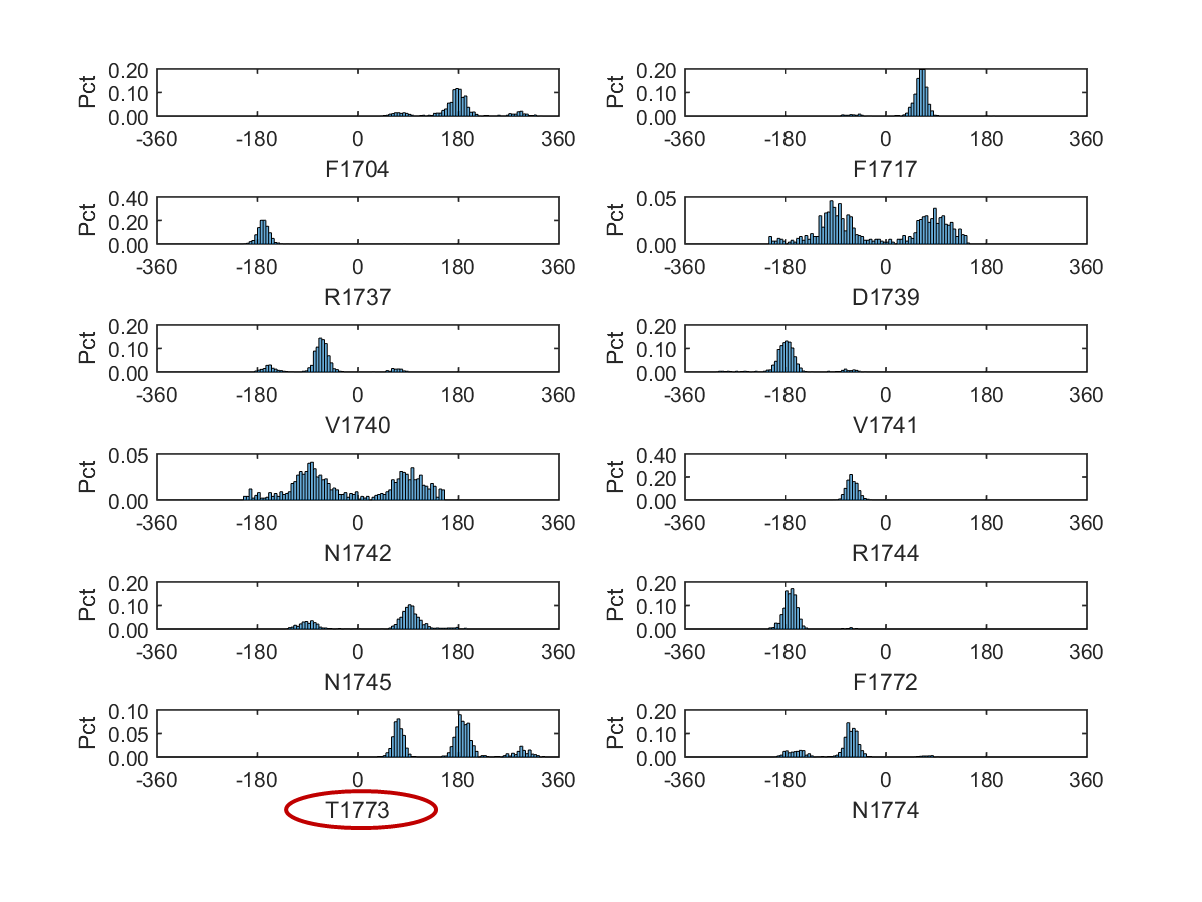


(A2)

The first side-chain dihedral angles of part of live set residues of P4-BRCT complex from MD


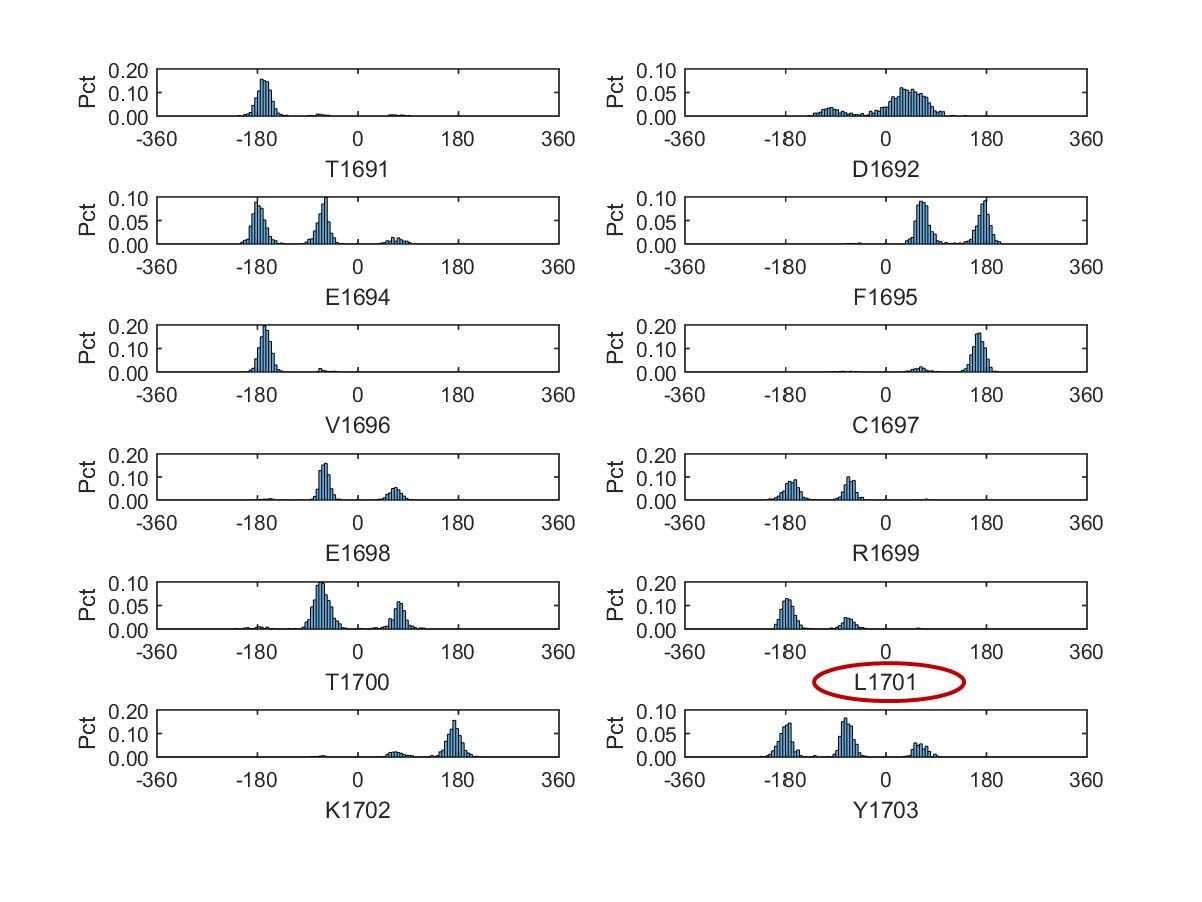

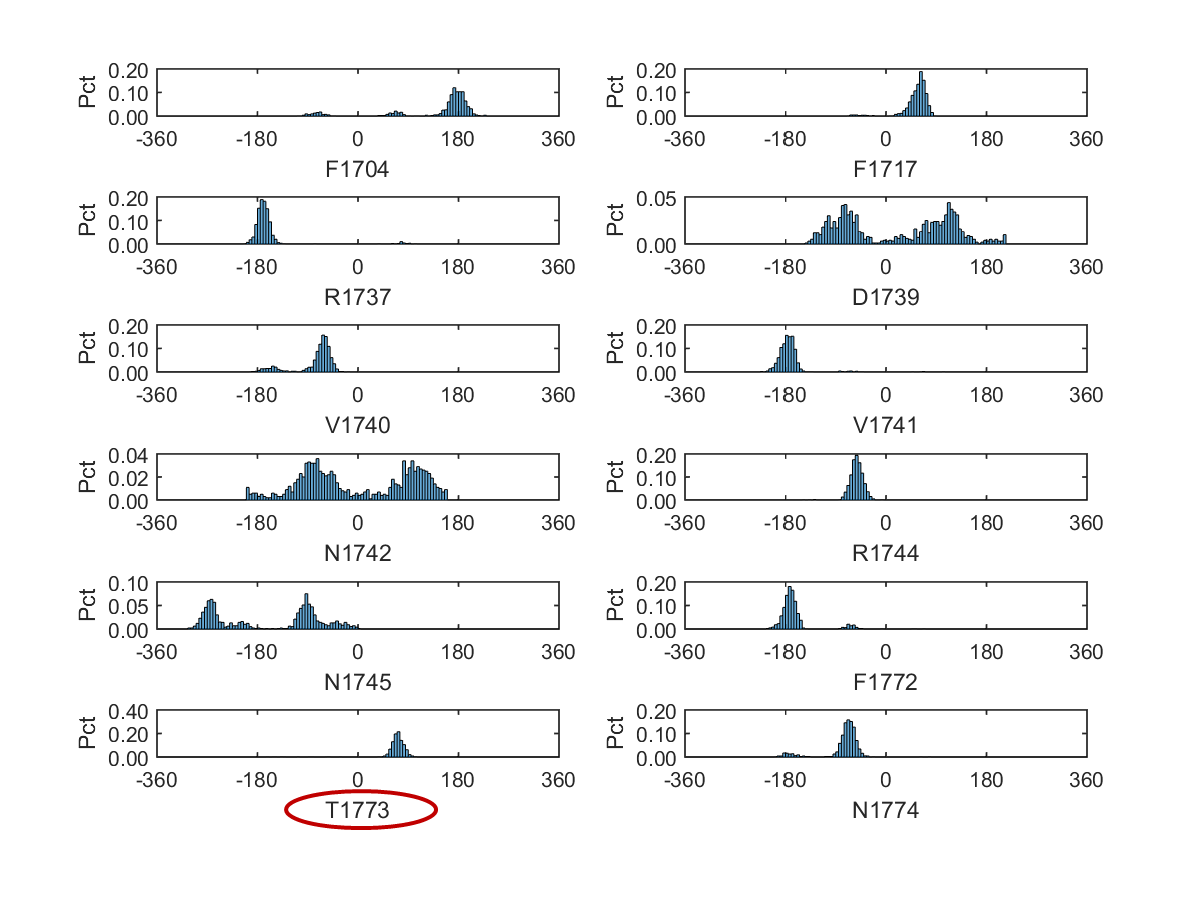


(B1)

The first side-chain dihedral angles of part of live set residues of C1-BRCT complex from M2


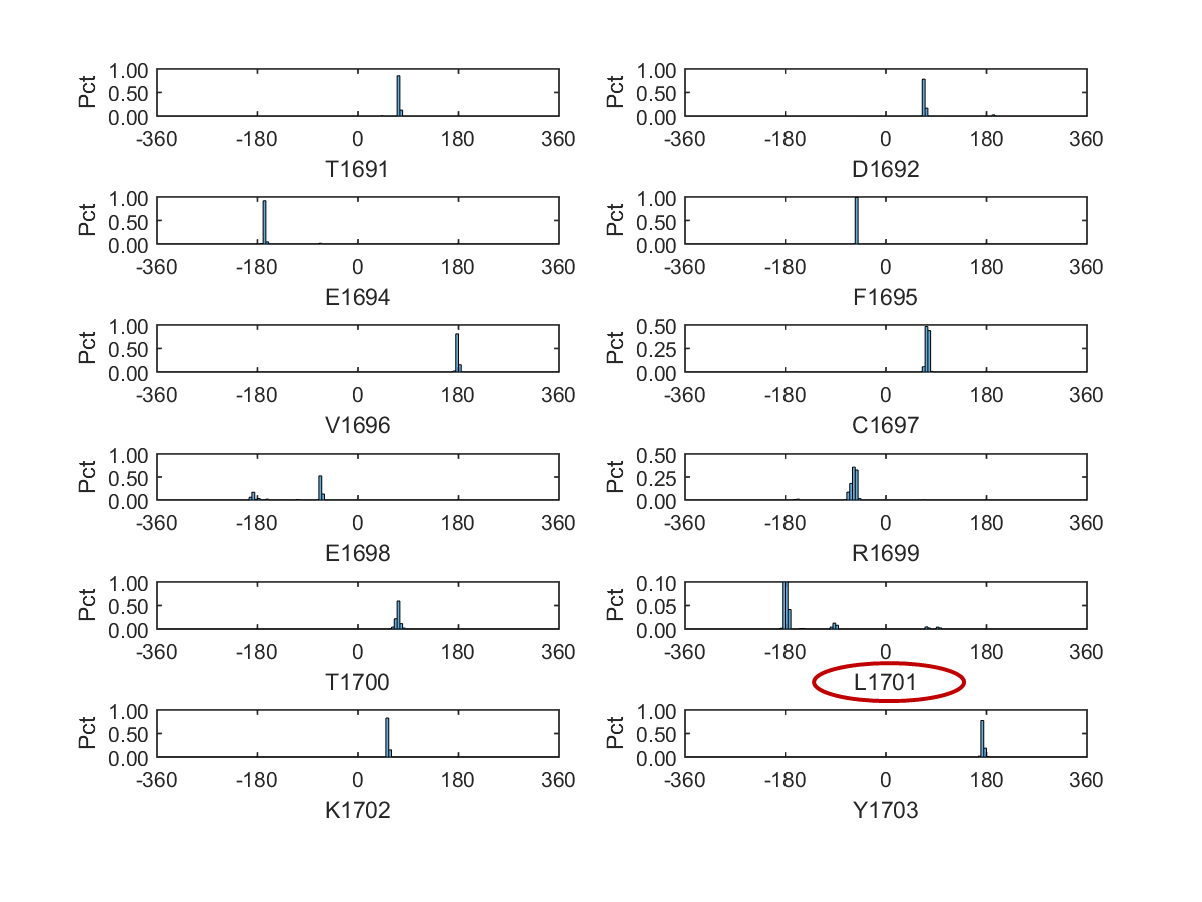

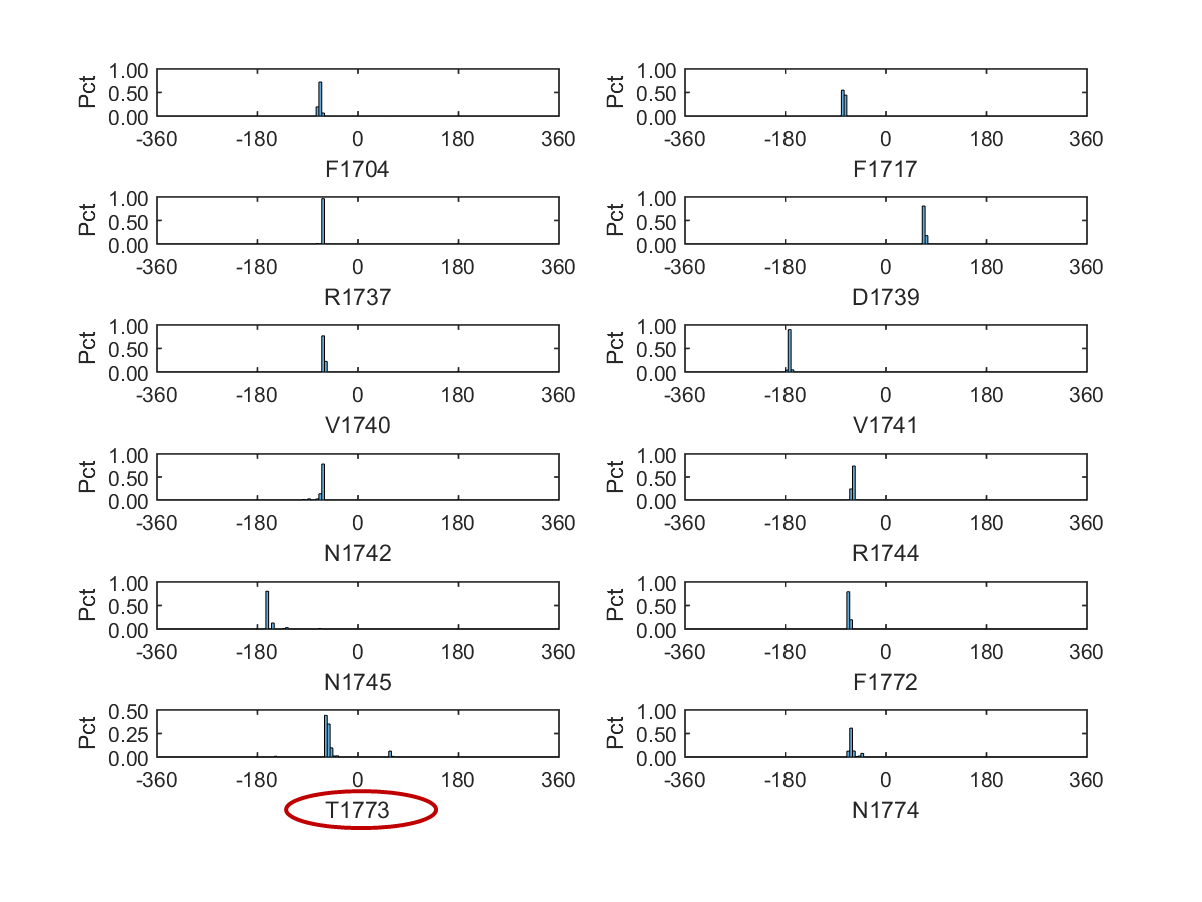


(B2)

The first side-chain dihedral angles of part of live set residues of P4-BRCT complex from M2


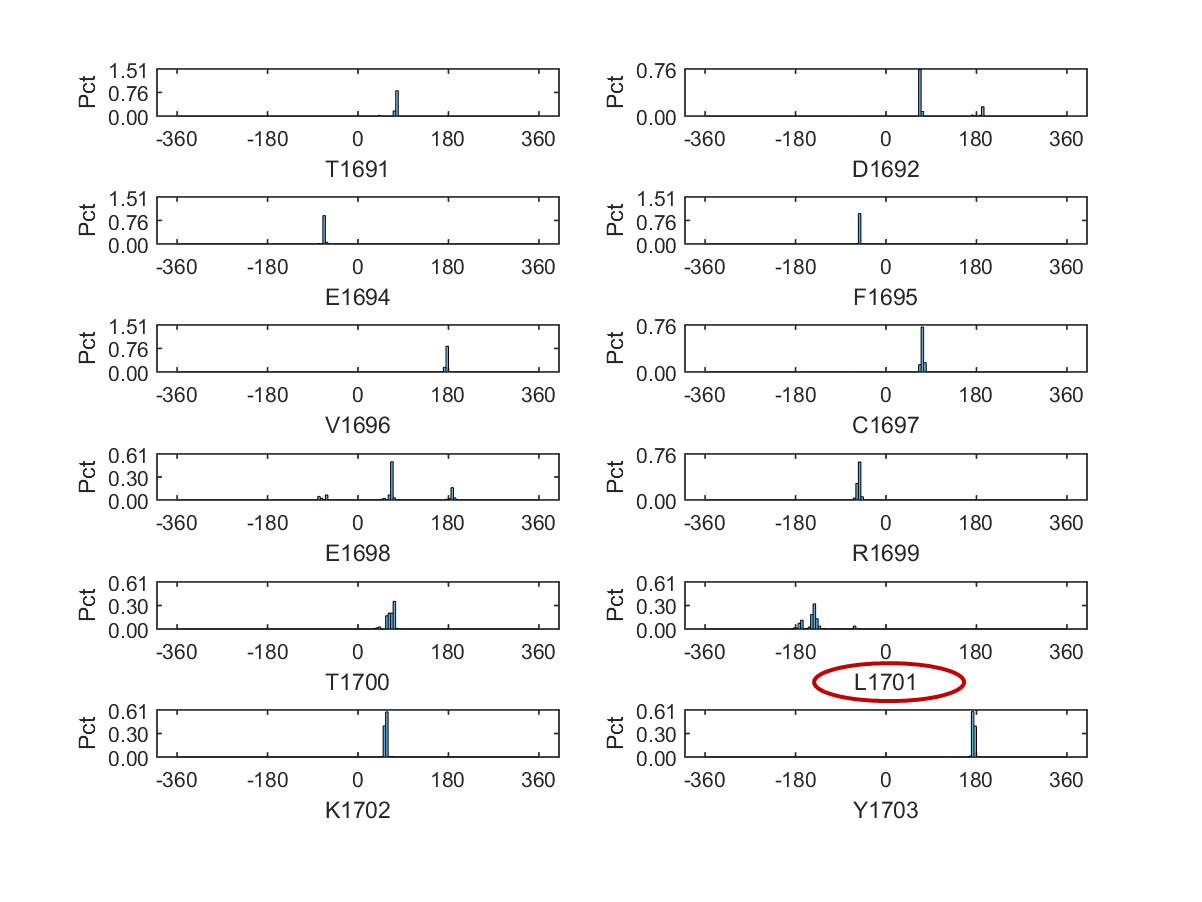

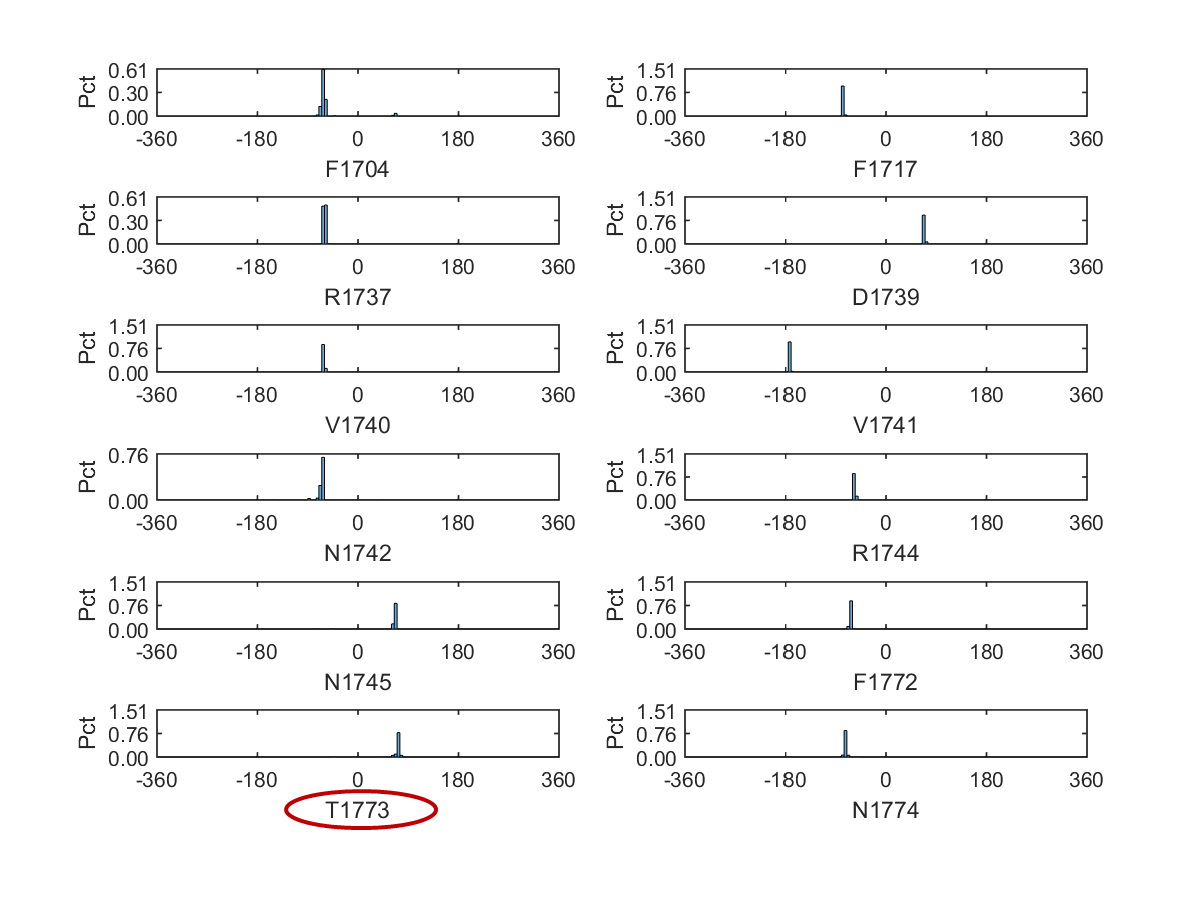

Supplement: S8 Fig — (A1), (A2). The first side-chain dihedral angles of part of live set residues of C1 and P4-BRCT complexes from MD, respectively. (B1), (B2). The first side-chain dihedral angles of part of live set residues of C1 and P4-BRCT complexes from M2, respectively. The difference is highlighted by red circle. (DOCX) [file pcbi.1005057.s011.docx]
